# The role of allotropy on phase formation in high entropy alloys

**DOI:** 10.1038/s41598-025-17217-5

**Published:** 2025-08-26

**Authors:** Kevin Kaufmann, Haoren Wang, Jaskaran Saini, Kenneth S. Vecchio

**Affiliations:** 1 Department of NanoEngineering, UC San Diego, La Jolla, CA 92093 USA; 2 Materials R&D, Oerlikon Metco (US) Inc., San Diego, CA 92127 USA

**Keywords:** High entropy alloys, Material informatics, Solid solution, Allotropism, Computational model, Crystal structure, Materials science, Structural materials, Metals and alloys

## Abstract

Identifying single phase, high-entropy systems has been a prominent research focus of materials engineering over the past decade. The considerable effort in computational modeling and experimental verification has yielded several methods and descriptors for predicting if a single phase will form; however, the details surrounding the resulting crystal structure have largely remained a mystery. Here, we present a compelling argument for the role of allotropy in determining the crystal structure of a single-phase, high-entropy alloy. High entropy alloys can contain 5 or more elements and must achieve a configurational entropy greater than 1.5R. This study shows that when these high entropy material conditions are met, the majority crystal structure of the non-allotrope forming element plays a dominant role in crystal structure determination. The theory is demonstrated via several approaches, including analysis of 434 unique known single-phase compositions from the literature, thermodynamic modeling of more than 1400 compositions, and experimental synthesis of nine specific alloys that test this hypothesis. The results demonstrate allotropy can identify a subset of compositions unlikely to form a single phase and predict the crystal structure with a high degree of accuracy for a wide range of simple (e.g., 5 equiatomic elements) and more complex (e.g., Al_0.3_B_0.6_CoCrFeNiCu_0.7_Si_0.1_) high entropy alloys. Allotropy provides new insight into the underlying physics governing the resultant crystal structure in materials without a principal element. As high entropy materials continue to be an area of focus for developing materials with unique properties, this study is expected to serve as a significant tool in the screening of materials for specific crystal structures.

**Supplementary Information:**

The online version contains supplementary material available at 10.1038/s41598-025-17217-5.

## Introduction

Since Yeh et al.^1^ and Cantor et al.^2^ independently described multicomponent alloys without a principal element in 2004, considerable research efforts have been directed toward this vast and largely unexplored composition space^3^. This region of chemical space is commonly referred to as complex concentrated alloys, multi-principal element alloys, or high-entropy alloys (HEAs). However, high entropy alloys must contain five or more elements with the goal of increasing the total entropy change (i.e., entropy metric^4^) toward a negative Gibbs free energy (ΔG) thus resulting in a single phase^5,6^. To achieve ‘high entropy’, the configurational entropy must be greater than 1.5R, where R is the gas constant^4^. The impact of this strategy is perhaps best exemplified by the number of material classes where high entropy materials have been discovered, often with unique and desirable properties^1,2,5,7–27^. Unfortunately, the original hypothesis that significantly more multi-component systems would be entropically stabilized into a single-phase solid solution has proven untrue^28^. The most common single phase microstructures for high entropy alloys to form are face-centered cubic (FCC), body-centered cubic (BCC), and occasionally hexagonal (HEX), while other crystal structures occur very rarely^7^. Presently, the scientific community considers the BCC phase as most likely to form, owing to its ability to accommodate larger ranges of atomic size in the same lattice^29^. However, recent reviews of HEAs reveal the FCC crystal structure to be more common than anticipated, accounting for nearly 50% of the aggregated results^7,22,29^. Furthermore, materials such as the Cantor alloy (CrMnFeCoNi) require further explanation as only Ni is FCC (20 at%), Cr is BCC, and Fe, Mn, and Co are allotrope elements with room temperature and atmospheric pressure structures of BCC, BCC, and HEX, respectively; and yet FCC is the resultant structure^2^. Miracle et al. performed an overview statistical analysis of HEAs called ‘structure in – structure out’ in which they concluded that most known HEAs are comprised primarily of FCC, BCC, and HEX elements, and thus, it should not be surprising that more complex structures are rarely reported (see Sect. 4.3.1.3 in^7^. However, the work does not individually assess HEA compositions to determine why they formed a particular structure, nor does it explain why compositions with relatively small amounts of a particular crystal structure still adopt that phase, both of which are addressed herein. An example of the new insight provided by this more in-depth analysis through the lens of allotropy is the perceived relative scarcity of FCC HEAs compared to BCC. There are only 11 non-allotrope FCC elements on the periodic table and excluding the six precious metals and actinium (none of which are generally commercially viable) leaves only Al, Cu, Ni, and Pb. Of those four elements, Pb is another element not typically used for commercial applications and Cu exhibits a miscibility gap with most other elements. Thus, materials engineers are reliant primarily on Al and Ni to form FCC HEAs. The need for more insight into crystal structure formation in HEAs is imperative given the large, complex composition space^30^ that has been unlocked by the idea of searching for the next impactful material near the largely unexplored center of phase diagrams.

Despite the intense effort to identify new high entropy alloys, the challenge of determining a priori, (which compositions are likely to form a single phase and which crystal structure the phase will adopt) remains non-trivial. This work is primarily focused on the latter part of the HEA design process; assuming a single phase will form, what crystal structure should be expected? A fundamental understanding of this phenomena is expected to serve as an important tool in the screening of materials for specific applications, since the intrinsic properties of alloys are highly dependent on the resultant phase(s)^7,19,31^. For example, FCC and BCC structured HEAs generally exhibit a tradeoff between good ductility and higher strength^32,33^. Existing in silico strategies generally employ some combination of first-principles density functional theory (DFT), thermodynamics, machine learning, and compositional descriptors as screening techniques. DFT-based strategies are a useful technique in the search for new alloys; however, the computational expense becomes impractical for dealing with the large simulation cells required to assess HEAs with 5 + elements and can require hundreds of hours of computation per composition^10,34–37^. Thermodynamic-based strategies, namely the CALPHAD method^38–41^, rely on thermodynamic databases of assessed systems, and therefore, perform best in regions of chemical space where significant data are available. Given the infancy of the high entropy materials field and the current resistance to publishing negative results (i.e., multiphase compositions), the reliability of this method is expected to be reduced for the vast majority of HEA candidates^11^. Machine learning-based methods are a relatively new approach to searching for HEAs and often leverage data from DFT, CALPHAD, and/or physicochemical descriptors^42–48^. The set of attributes most used to predict phase formation of alloys include a combination of: (1) the mixing enthalpy ($\:\varDelta\:{H}_{mix}$), (2) valence electron configuration (VEC), (3) atomic size ratios (δ), (4) configurational entropy (S), and (5) Pauling electronegativity (χ)^49–51^. There are a plethora of other descriptors tested during model fitting; however, these five attributes are the most common across multiple HEA-design campaigns and are typically found to be the most important to machine learning models’ decision making process^42,43^. Despite efforts in the material informatics field to link these descriptors with phase formation, there remains significant overlap of the different predicted phases by application of the derived models (refer to Chap. 2 in Reference^36^. Additionally, the presence of specific elements (i.e., Al, Cu, Li, Mg, Sn, and Zn) are known to limit the effectiveness of these phase formation rules^52^. Since existing descriptors are largely derived from the Hume-Rothery rules, their predictive capabilities in the HEA composition space are expectedly limited by their origination from binary alloys^53^.

In stark contrast to the approaches described in the above discussion, allotropism provides a simple method to reliably predict and explain the physical phenomena underlying the resultant crystal structure. Allotropism is the property of some elements to exist in two or more different structures in the same physical state of matter (e.g., liquid or solid). For example, some allotropic forms of solid carbon are diamond, graphite, lonsdaleite, and fullerenes. In the context of this work, elements are considered to be “allotrope forming” if more than one crystal structure exists for the solid state; no other states of matter will be considered. For instance, solid iron changes from a body-centered cubic structure (ferrite) to a face-centered cubic structure (austenite) above 906 °C. On the other hand, the “non-allotrope” elements (e.g., Ni or V) are only known to exist in one crystal structure as solids. The role of this property on HEA phase formation, to the best of these authors’ knowledge, has not been discussed previously in the literature, and yet, it will be demonstrated that the majority crystal structure of the non-allotrope elements present in a given composition is a dependable predictor of the final structure. While not predictive of whether or not a single-phase will form, allotropy can assist with defining the compositional search space for an HEA with a desired crystal structure. In addition, thermodynamic modeling of compositions containing equal parts FCC and BCC non-allotrope elements will be compared to the modeling of known HEAs to demonstrate that the absence of a dominant (in atom percent) non-allotrope structure (and absence of allotrope forming elements to provide crystal structure “flexibility”) prevents the formation of a single phase. These principles are an effective tool to assist in the development of new HEAs and predict their structure a priori.

## Methods

### Known single phase HEA materials

The set of materials known to form a single phase is obtained from the 2019 book on HEAs by Murty et al.^29^ and the 2018 database of HEAs compiled by Gorsse et al.^22^. The tables of materials in each source were converted to Excel documents using the AWS Textract API and subsequent compositional analyses performed using Python and the Pandas software package^54^. The datasets are presented separately herein. Between the two datasets, there are 434 unique single phase HEA compositions comprising a diverse region of chemical space.

### Selection of new alloys

The new HEA candidates for testing a null hypothesis of the allotropy model (i.e., equal atom percent of FCC and BCC elements will form a single phase) were selected for modeling and potential fabrication using three rules: (i) all of the elements in the composition must be BCC or FCC and not exhibit allotropism; (ii) all of the elements must be available in the ThermoCalc TCHEA5 (high entropy alloys) database; and (iii) the BCC elements must sum to 50 atom percent, and the FCC elements must sum to 50 atom percent. The FCC elements that satisfy these criteria are Al, Cu, Ni, Ir, and Rh; and the BCC elements are Cr, Mo, Nb, Ta, V, and W. All possible 5 + element combinations of these FCC and BCC non-allotrope elements are then input to ThermoCalc. The combinations with only FCC or BCC elements (e.g., CrMoNbTaV) were excluded from the calculation. The intent of these compositions is to demonstrate the importance of allotropism on single versus multi-phase formation.

### Thermodynamic modeling

Thermodynamic modeling was performed using the ThermoCalc Software TCHEA5 database^55^. Each composition is modeled from 2500 °C down to 500 °C in 100 °C steps, and the number of solid phases at each temperature recorded. The presence of a liquid phase is also recorded, and the composition not considered single phase if either a liquid or more than one solid phase is predicted.

### Experimental synthesis and characterization

Nine alloys from those described in Sect. 2.2 are selected for fabrication based on the thermodynamic modeling results. Ingots are fabricated via arc melting > 99.9% purity slugs of the individual elements (Thermo-Fisher) under a Ti-gettered argon atmosphere. High and low melting temperature elements were initially melted separately and subsequently combined to ensure all elements were present in the final compositions at the correct atomic percentages. Samples were flipped and remelted ten times to maximize homogeneity. The sample predicted to be single-phase is then annealed for 24 h in a Red Devil™ vacuum furnace (RD WEBB, USA) at an appropriate temperature as determined by thermodynamic modeling. Target chemistry is verified, and chemistry maps are collected using a Thermo-Fisher Apreo scanning electron microscope (SEM) equipped with an Oxford X-Max^N^ EDS detector. Phase analysis is performed using an Anton Paar XRDynamic 500 X-ray diffraction (XRD) unit equipped with a one-dimensional detector. XRD data are collected from 20° to 120° (2θ angles) with a 0.02° step size and scan rate of 5°/minute. Copper K_α_ radiation is used for all x-ray diffraction measurements.

## Results

### Analysis of known single phase HEAs

**Fig. 1:**
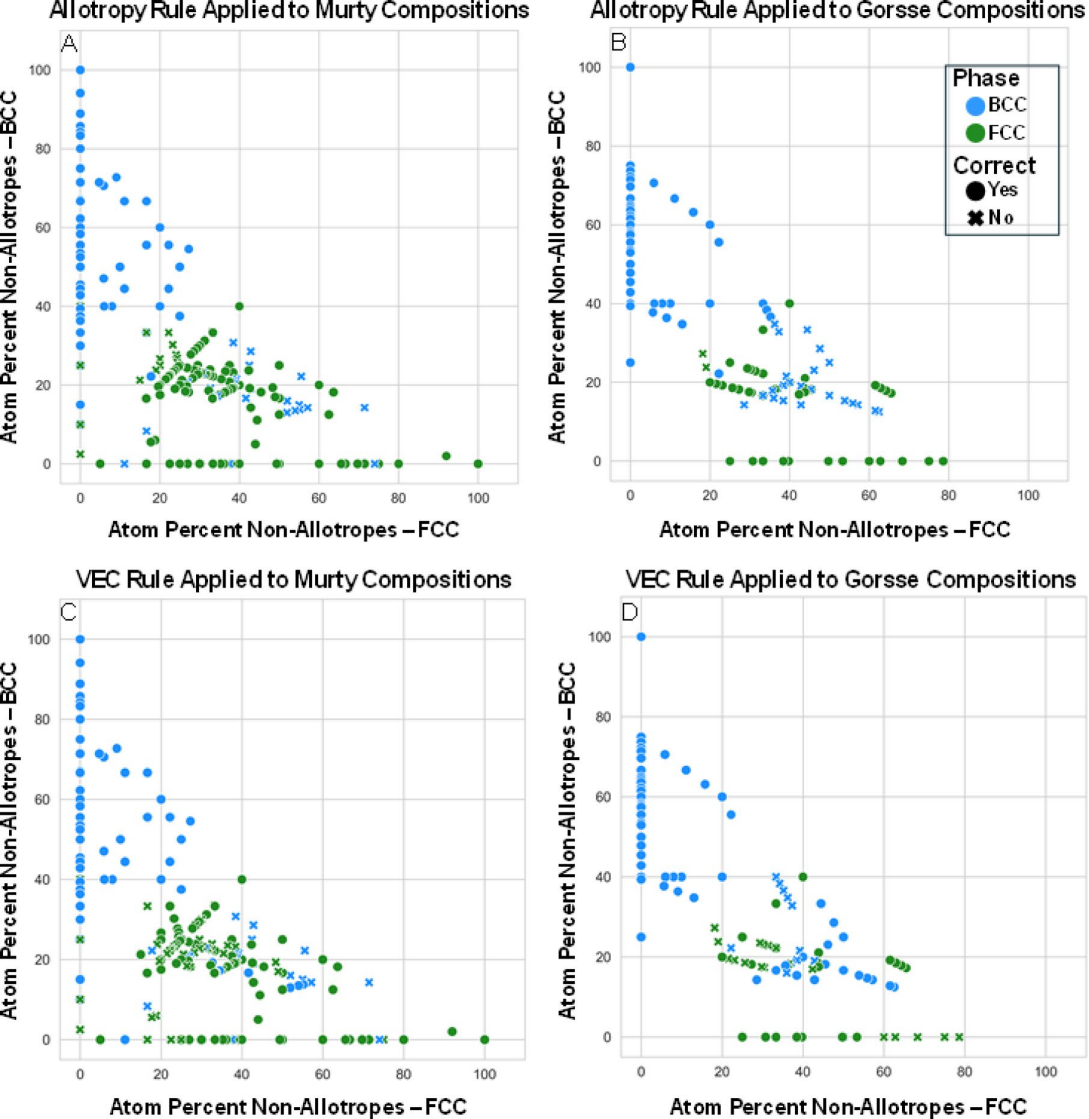

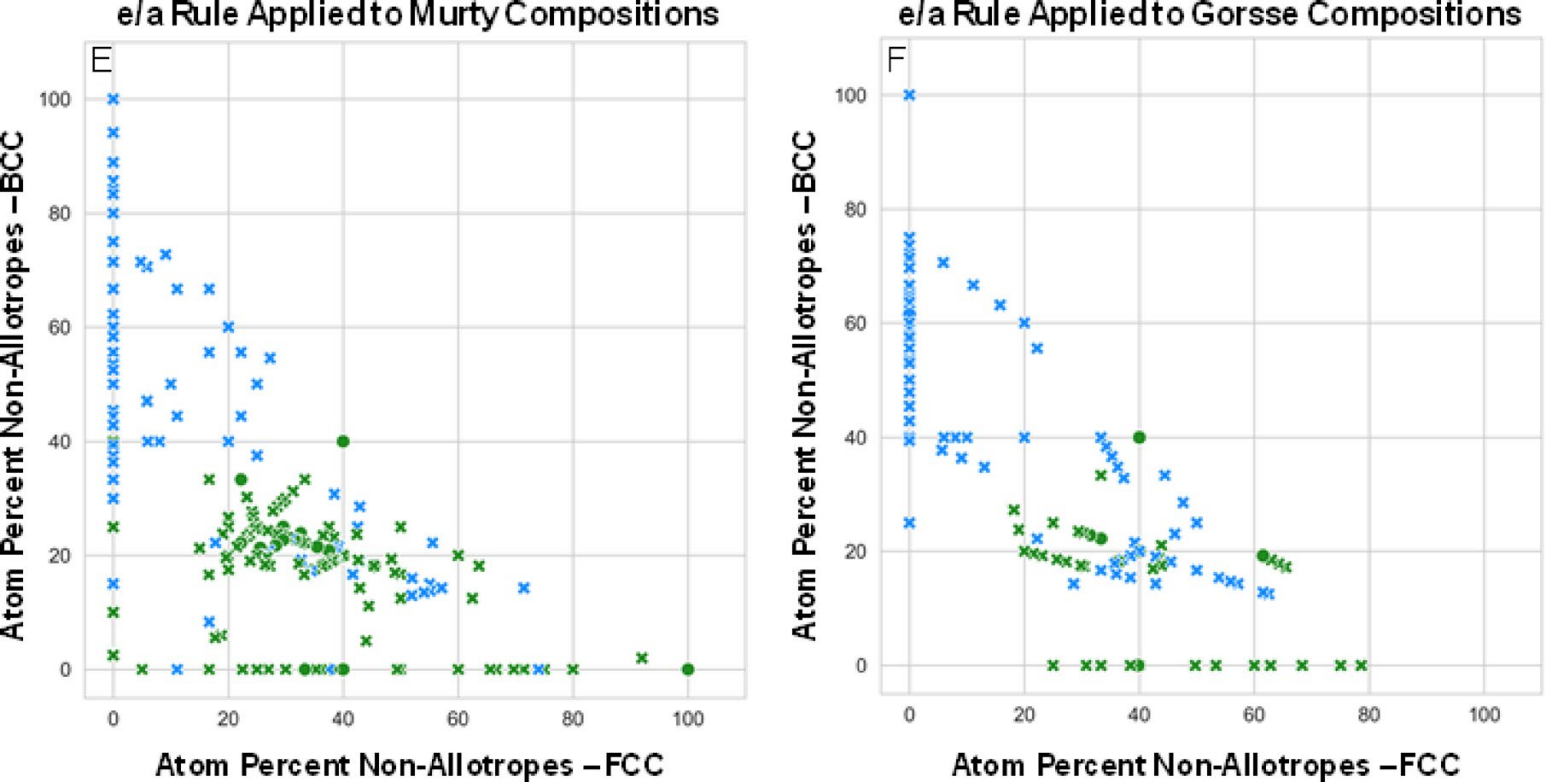
Non-allotrope atom percent compared to phase formation. Each data point details the atom percent of FCC and BCC non-allotrope elements for the HEA compositions in **A**,**C**,**E**) Murty et al.^29^, and **B**,**D**,**F**) Gorsse et al.^22^. The allotropy descriptor is applied for phase prediction in panel A and B. The VEC descriptor is applied for phase prediction in panel C and D. The e/a ratio descriptor is applied for phase prediction in panel E and F. Individual compositions marked with a circle are correctly identified by the respective model, while compositions marked with an “X” are not. The color of each data point corresponds to the experimentally reported phase, BCC (blue) or FCC (green). Data points will not add up to 100 atomic percent if any allotrope forming elements are present in the composition.

The first demonstration of the predictive power of non-allotrope elements is presented in (Table 1). Summary statistics are calculated for the 484 HEAs reported in Murty et al.^29^ and Gorsse et al.^22^. Of the 484 reported HEAs, 434 are of a unique composition; however, the data from each source is reported and analyzed in its entirety. The atomic percent of non-allotrope elements in the reported HEAs ranges from 2.50 to 100%. The number of FCC and BCC alloys is reported along with the percentage of alloys for which the structure was correctly predicted using the proposed allotropy-based descriptor (e.g., the column ‘Accuracy for HEAs Primarily Containing BCC Non-allotropes’). An alloy ‘primarily containing BCC non-allotropes’ means there is a greater amount of BCC non-allotrope elements in the composition. The percentage in Table 1 details the percentage of the BCC HEAs reported for which the BCC non-allotrope atom percent is the majority elemental crystal structure present in the HEA and thus are correctly predicted by the allotropy model (i.e., 90% overall for BCC alloys). To provide an example, AlCoCuFeNiV is composed of 66.7 atom percent non-allotrope elements, and 50 atom percent of the composition is FCC elements, 16.7 atom percent are BCC, and the remaining 33.3 atom percent is allotrope-forming elements. Since the composition is reported as FCC, it counts toward the percentage of alloys that crystallize FCC and FCC is the dominant non-allotrope structure (see Table 1). The macro-averaged precision and recall for the allotropy model are 77% and 76%; respectively. The FCC or BCC crystal structure predictions using traditional descriptors VEC and e/a ratio were also tabulated using the criteria from Ref^36^. The macro-averaged precision and recall for the VEC model are 63% and 47%; respectively. The macro-averaged precision and recall for the e/a ratio descriptor are 66% and 5.6%; respectively. The F1-score, or the harmonic mean of the precision and recall scores, is computed to be 0.76 for allotropism. The F1-score for the VEC descriptor is 0.54 and the F1-score for the e/a ratio is 0.098. Interpreting the F1-score depends on the domain and context of the problem being addressed; however, scores greater than 0.70 generally signify the model is good at identifying both positive and negative cases, which the allotropy descriptor alone achieves. Ultimately, the allotropy descriptor shows at least 10% higher precision, recall, and F1-score than either VEC or the e/a ratio in predicting HEA crystal structure. Refer to Supplementary Table 1 and Supplementary Table 2 for the complete analysis results, including HEAs with crystal structure other than FCC and BCC, the percentage of allotrope forming and non-allotrope elements per composition, and whether the predominant non-allotrope elements are FCC or BCC in each alloy. The data for calculated valence electron concentration (VEC), electron per atom (e/a) ratio, mixing enthalpy ($\:{\varDelta\:H}_{mix}$), and atomic size mismatch (δ) are in Supplementary Table 3 and Supplementary Table 4. Figure 1 visually summarizes this data by plotting the atom percent of non-allotrope FCC and non-allotrope BCC elements for each composition, as well as identifies whether the allotropy (Fig. 1A, B), VEC (Fig. 1C, D), or e/a ratio (Fig. 1E, F) model would correctly identify the resultant crystal structure.

**Table 1:** Allotropism-based analysis of HEAs. Summary statistics for the FCC and BCC HEAs reported by Murty et al. and Gorsse et al. and the combined data in row ‘Total’. The number of single-phase alloys in each work is reported along with the number that are FCC and BCC. For the FCC (or BCC) alloys, the percentage of alloys that crystallize in the FCC (or BCC) structure when the majority non-allotrope crystal structure is also FCC (or BCC) is detailed. For example, Gorrse et al. contains 95 BCC heas. of which 97.9% contain a greater amount of BCC non-allotrope elements than FCC non-allotrope elements (i.e., BCC dominant).

Source	Number of alloys	# of FCC alloys	Accuracy for HEAs primarily containing FCC non-allotropes﻿ (%)	# of BCC alloys	Accuracy for HEAs primarily containing BCC non-allotropes (%)
*Murty*	323	165	67.9	139	84.9
*Gorsse*	161	53	41.5	95	97.9
Total	484	218	61.5	234	90.2

### Thermodynamic modeling data

**Fig. 2:**
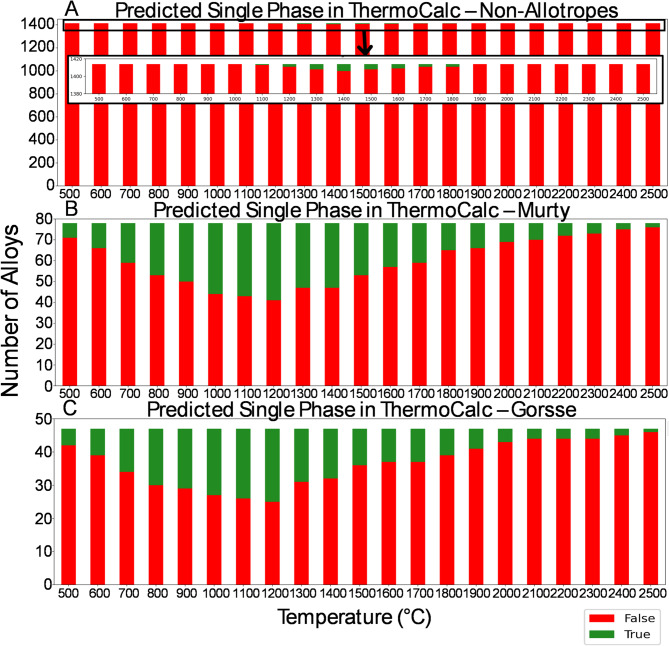
Number of compositions predicted single phase in CALPHAD. Thermodynamic modeling is applied to assess the likelihood of equilibrium single phase formation for (**A**) the previously unreported compositions containing an equal atom percent of five or more FCC and BCC non-allotrope elements, (**B**) the HEAs from Murty et al.^29^, and (**C**) the HEAs from Gorsse et al.^22^. The inset in (**A**) is a magnified view showing the miniscule number of compositions predicted to be single-phase HEAs.

Thermodynamic-based modeling of the HEAs from each of the two databases and the 1,414 new compositions containing only equal atom percent of five or more FCC and BCC non-allotrope elements was performed using the ThermoCalc TCHEA5 database. The number of compositions calculated from the *Murty* and *Gorsse* works was reduced to 78 and 47 unique compositions, owing to the element restrictions of the thermodynamic database. For a given composition and temperature, it was recorded whether the alloy was predicted to be a single-phase solid-solution (True) or not (False) (see Fig. 2). Supplementary Fig. 1 provides a visual summary of the number of phases predicted for each of the compositions studied. In addition to the bar chart summing the number of True and False readings at each temperature, Table 2 details the percentage of alloys that were modeled to be single phase at any temperature. Furthermore, Table 2 also includes the average and standard deviation of the temperature range for which the alloys are predicted to be single phase. Figure 2A; Table 2 highlight the miniscule likelihood of one of the new non-allotrope compositions being a high entropy alloy. Particularly when compared to the results for the HEAs from the *Murty* and *Gorsse* datasets, wherein more than 50% of the modeled HEAs are predicted to be single phase somewhere between 2500 °C and 500 °C and with a wide average single-phase range of ~900 °C ± 500 °C. The accuracy of thermodynamic calculations are, of course, dependent on the modelled and integrated phases and chemical systems that have been evaluated in the thermodynamic TCHEA5 database. However, the magnitude of difference (approximately 1% vs. 55%) in compositions suggested to potentially be single-phase at some temperature when searching nearly 1,500 new compositions suggests the likelihood of fabricating a single-phase material experimentally is unlikely.

**Table 2:** Thermodynamic analysis of HEA phase formation. ThermoCalc single phase predictions for HEAs from each literature source and the equiatomic FCC and BCC non-allotrope compositions. The number and percentage of HEAs predicted to form a single phase at any temperature is reported. Additionally, the average and standard deviation of the temperature range for which those compositions are predicted to be single phase is also presented.

Source	Number of alloys	Single phase at any temperature	Average single phase temperature range (°C)
Equal FCC/BCC non-allotropes	1414	12 (0.8%)	300 °C ± 200
*Murty*	78	45 (57.7%)	900 °C ± 500
*Gorsse*	47	26 (55.3%)	800 °C ± 500

### Characterization of fabricated alloys

**Fig. 3:**
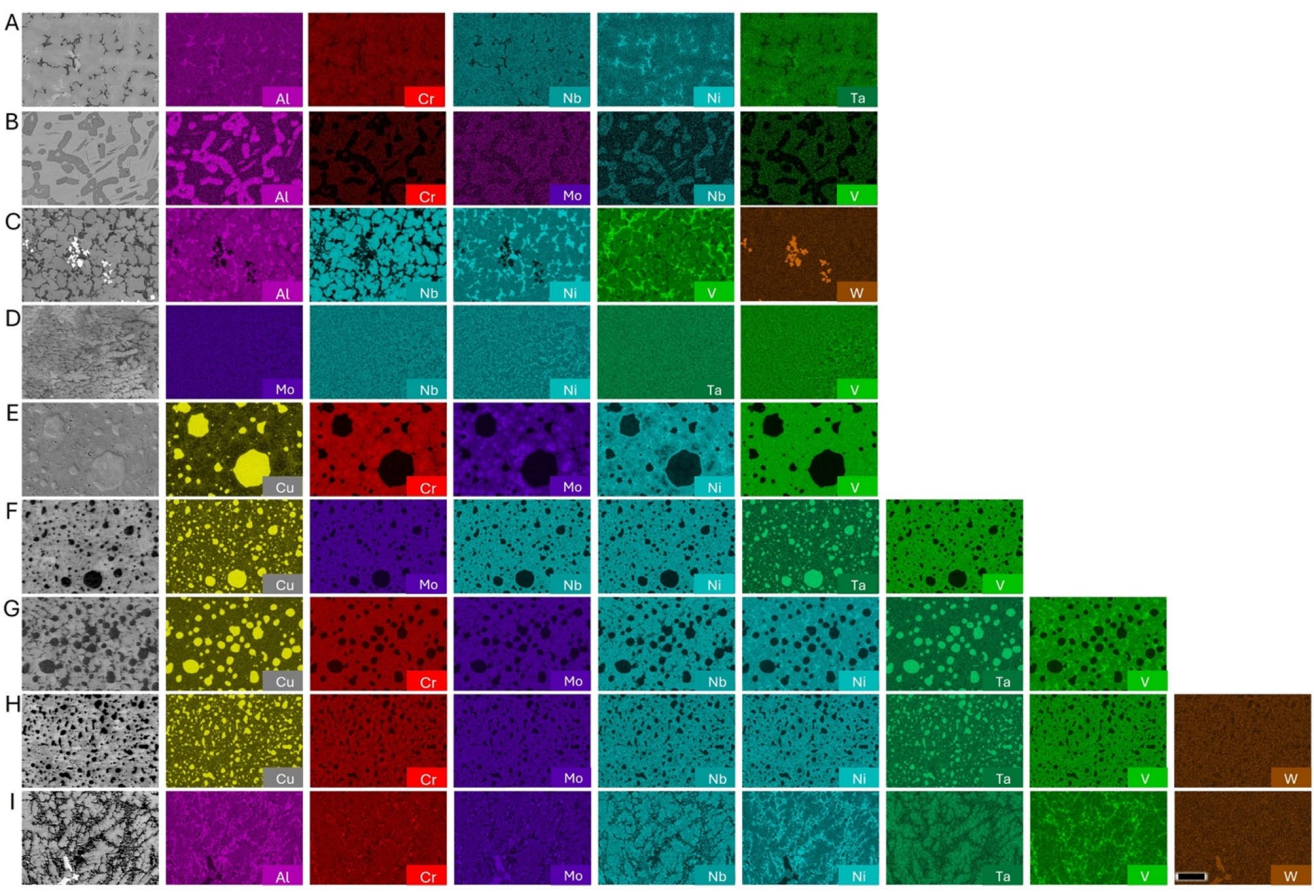
Characterization of fabricated compositions. An electron image is included with the EDS maps for (**A**) Al_8.90_Cr_11.45_Nb_20.45_Ni_19.37_Ta_39.83_ (**B**) Al_27.00_Cr_13.01_Mo_24.00_Nb_23.24_V_12.74_ (**C**) Al_8.87_Nb_20.37_Ni_19.30_V_11.17_W_40.30_ (**D**) Mo_14.64_Nb_14.17_Ni_35.81_Ta_27.60_V_7.77_ (**E**) Cr_13.61_Cu_24.93_Mo_25.10_Ni_23.03_V_13.33_ (**F**) Cu_19.11_Mo_14.42_Nb_13.97_Ni_17.65_Ta_27.20_V_7.66_ (**G**) Cr_6.68_Cu_20.41_Mo_12.33_Nb_11.94_Ni_18.85_Ta_23.25_V_6.54_ (**H**) Cr_5.08_Cu_18.65_Mo_9.37_Nb_9.07_Ni_17.21_Ta_17.68_V_4.98_W_17.96_ and (**I**) Al_8.87_Cr_5.69_Mo_10.50_Nb_10.17_Ni_19.28_Ta_19.80_V_5.57_W_20.12_. Each sample is observed to be multi-phase as arc melted. All compositions are provided in weight percent. Note that Ta and Cu have overlapping characteristic X-ray peaks. The scale bar in the lower right tungsten EDS map is 25 μm.

**Fig. 4:**
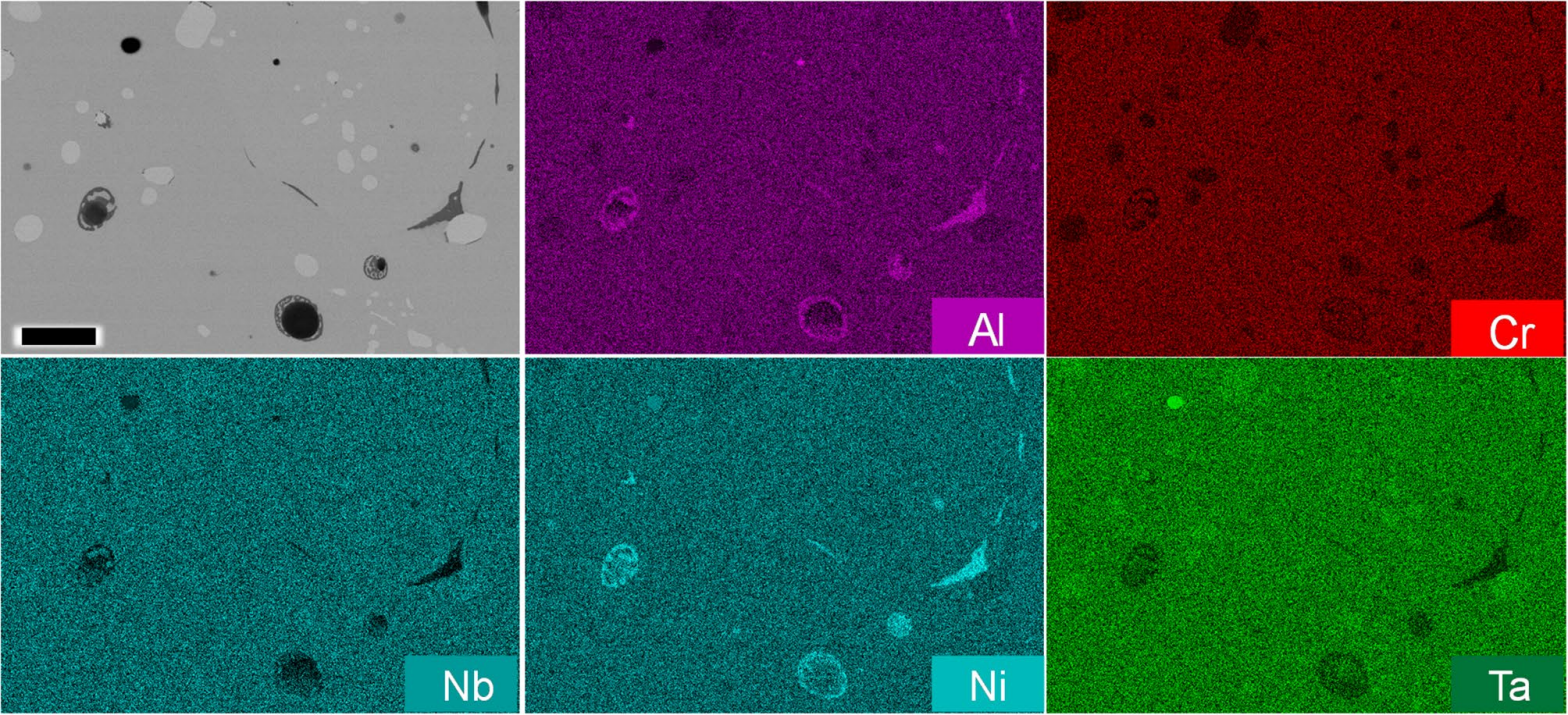
Chemistry maps for annealed Al_8.90_Cr_11.45_Nb_20.45_Ni_19.37_Ta_39.83_. An electron image is included with the EDS maps for Al_8.90_Cr_11.45_Nb_20.45_Ni_19.37_Ta_39.83_ after annealing at 1475 °C in an inert environment. The sample remains multi-phase. The pores are attributed to Kirkendall voids. The scale bar is 25 μm.

From the compositions containing an equal atom percent of BCC and FCC non-allotrope elements, only 12 were predicted to be single phase over the modeled temperature range (Table 3). Of those 12, only 1 composition does not contain any of the prohibitively expensive elements Ir or Rh. The composition, Al_8.90_Cr_11.45_Nb_20.45_Ni_19.37_Ta_39.83_, is suggested to be stable within an approximately 300 °C window between 1300 and 1600 °C. Intriguingly, the single-phase crystal structure predicted to be stable is the HEX C14 Laves phase. This alloy along with eight other compositions that would be an HEA if single phase were fabricated to experimentally test the computationally supported hypothesis that a given composition, without an atom percent majority of non-allotrope elements having a particular crystal structure, and without the presence of allotrope forming elements, is unlikely to be an HEA. Chemistry maps for these nine alloys showing the multi-phase results are shown (Fig. 3). The phase evolution diagrams from CALPHAD are shown in Supplementary Fig. 2; XRD data is provided in Supplementary Fig. 3. Figure 4 and Supplementary Fig. 4 confirm that the composition Al_8.90_Cr_11.45_Nb_20.45_Ni_19.37_Ta_39.83_ remains multi-phase even after annealing at 1475 °C (as suggested by ThermoCalc) for 20 h in a vacuum furnace. The combination of characterization data for these nine compositions highlights the multi-phase result for each sample and the improbability of designing a single-phase alloy with these constraints.

**Table 3:** New alloys predicted to be HEAs by CALPHAD. The twelve compositions predicted to form a thermodynamically stable single phase when modeled from 500 to 2500 °C are listed. Only the temperature range over which any compositions are predicted to be single phase is shown. TRUE denotes only a single phase is predicted to be formed, while FALSE indicates more than one solid phase, or a solid and liquid phase are present. The single phase range column reports the temperature range for which the single phase is thermodynamically predicted to be stable.

Composition (wt%)	1100 °C	1200 °C	1300 °C	1400 °C	1500 °C	1600 °C	1700 °C	1800 °C	Single phase range (°C)
Cr_15.34_Ir_37.83_Ni_11.55_Rh_20.25_V_15.03_	FALSE	FALSE	TRUE	FALSE	FALSE	FALSE	FALSE	FALSE	100
Cr_4.06_Ir_60_Mo_7.49_Ta_14.11_W_14.34_	FALSE	FALSE	FALSE	FALSE	FALSE	TRUE	TRUE	TRUE	300
Cr_4.52_Ir_66.76_Mo_8.33_V_4.42_W_15.96_	TRUE	TRUE	TRUE	TRUE	TRUE	TRUE	TRUE	TRUE	800
Cr_6.55_Mo_12.08_Rh_5.18_V_6.41_W_23.14_	FALSE	TRUE	TRUE	TRUE	TRUE	TRUE	FALSE	FALSE	500
Cr_9.04_Ir_50.11_Mo_16.68_Ni_15.30_V_8.86_	FALSE	FALSE	TRUE	TRUE	FALSE	FALSE	FALSE	FALSE	200
Ir_24.86_Mo_18.60_Ni_7.59_Rh_13.31_W_35.65_	FALSE	FALSE	FALSE	FALSE	FALSE	TRUE	FALSE	FALSE	100
Ir_33.48_Mo_25.06_Ni_10.22_Rh_17.93_V_13.30_	FALSE	TRUE	TRUE	TRUE	TRUE	FALSE	FALSE	FALSE	400
Ir_40.76_Mo_13.57_Ni_12.45_V_7.21_W_26.01_	FALSE	FALSE	FALSE	TRUE	FALSE	FALSE	FALSE	FALSE	100
Ir_60.04_Mo_7.49_Ta_14.13_V_3.98_W_14.36_	FALSE	FALSE	FALSE	FALSE	FALSE	TRUE	TRUE	TRUE	300
Al_10.27_Cr_13.21_Mo_24.37_Rh_39.20_V_12.94_	FALSE	FALSE	FALSE	TRUE	TRUE	FALSE	FALSE	FALSE	200
Al_10.35_Cr_13.31_Nb_23.78_Rh_39.51_V_13.04_	FALSE	FALSE	TRUE	TRUE	TRUE	FALSE	FALSE	FALSE	300
Al_8.90_Cr_11.45_Nb_20.45_Ni_19.37_Ta_39.83_	FALSE	FALSE	FALSE	TRUE	TRUE	FALSE	FALSE	FALSE	200

## Discussion

A new descriptor for phase formation and crystal structure prediction for high entropy alloys is presented and rigorously evaluated against existing knowledge. The singular descriptor, the phase fraction of non-allotrope elements of each crystal structure type, was foremost demonstrated to predict the FCC or BCC phase formation of known HEAs with overall accuracy of 71%. This level of performance is on par with or better than existing methods^10,36,56–59^, does not require threshold values that can vary across alloy systems^60^, and provides a descriptor based in physiochemistry. The ability to predict accurately the resultant FCC or BCC phase for a wide range of HEAs provides convincing validation and is a marked achievement. Additional compositions outside these datasets demonstrate continued success of the allotropy descriptor: CoCuFeNiPt (FCC)^61^, CoCrFeNiPd (FCC)^62^, CoCrFeNiPd_2_ (FCC)^62^, and Al_20_Li_20_Mg_10_Sc_20_Ti_30_ (HEX)^63^. Finding further examples of HEAs discovered since these datasets were compiled proved to be challenging owing to the large number of publications that study properties of well-established HEAs or report to have discovered a new “HEA” and then present multi-phase EDS or XRD data. Most incorrect predictions are for alloys containing at least 5 atomic percent Al and at least one other FCC element for which it is known to form the B2 phase (e.g., Ni). The few predictions that are wrong, when the allotropy model predicts the BCC phase, contain Cr or Nb, which are known Laves phase formers. While phases other than BCC and FCC are possible, they rarely occur as demonstrated previously^7^. Between the two databases of HEAs analyzed in this work, 95% are either FCC or BCC with HCP next most common, appearing in only eight instances. Therefore, elucidating the underlying mechanisms governing FCC and BCC phase formation is expected to play a pivotal role in advancing the design and development of high-entropy alloys. The presence of the light elements Cu, Li, Mg, Sn, and Zn does not reduce the accuracy of the model for FCC and BCC compositions, as it known to using other descriptors^52^. It is noted that the high temperature crystal structure of the allotrope elements (e.g., FCC for Fe) may also play a role in crystal structure determination, particularly for compositions with small amounts of non-allotrope elements or when the processing route involves high temperatures. This may be useful as a second allotropy-based descriptor in future modeling of HEA phase formation. Despite the few incorrect predictions described, allotropy is likely to become an important feature in future modeling approaches and may further improve their predictive performance.

Subsequently, the importance of allotropism on single phase formation was demonstrated via an extensive thermodynamic modeling campaign and fabrication of several alloys with 5 to 8 non-allotrope elements in the ThermoCalc high entropy alloy database. The miniscule subset of non-allotrope compositions predicted to be single phase at any temperature, particularly compared to the known HEAs that could be modeled, suggests none are likely to form a single-phase HEA. The 11 materials not studied, owing to the expense of Ir and Rh, are likely to be incorrect predictions far from the composition space assessed to build the ThermoCalc database. Unfortunately, these elements are prohibitively expensive to study. Ultimately, without a dominant non-allotrope crystal structure, there does not appear to be a driving force toward a particular crystal structure, thus resulting in a multi-phase material. This implies a significant number of complex concentrated alloys can be ruled out as potential single phase HEAs.

One limitation of this study is the sheer number of ways the alloys could be processed in comparison with the methods utilized herein. Differences in processing (e.g., annealing temperature) can result in conflicting reports for the same composition (e.g., some Cantor alloys which are well known to decompose into multiple phases at lower temperatures). For example, the data compiled by Murty et al. lists 43 different processing routes^29^. The caveat to the numerous processing routes is that several, such as sputtering, mechanical alloying, melt spinning, and suction casting, are known to allow for metastable structures. One such example in the dataset is CrNbTiVZn (60% BCC and 20% HEX non-allotropes) processed by 60 h of mechanical alloying and reported to be single phase FCC by Dwivedi et al.^64^. In their own work, the authors recognize the metastable nature of the FCC phase achieved under non-equilibrium conditions. It is likely that other reports in the aggregated datasets are for non-equilibrium results. The number of compositions from the literature that ThermoCalc did not predict to be single phase at equilibrium supports but does not prove this statement. Thus, the allotropy model may achieve better performance than derived from analysis of the existing literature when considering whether the reported result is at equilibrium or not.

In the near term, it is expected that this work will be extended to the analysis of other crystal structures, such as the hexagonal phase HEAs. Additionally, this model provides specific guidance for the design of HEAs by offering a chemical basis for targeting specific crystal structures. Since there is no symbol representing allotropism or non-allotrope forming elements, the categorical descriptor $\:{NAF}_{major}$ for the majority non-allotrope forming crystal structure type (e.g., $\:{NAF}_{major}=\text{F}\text{C}\text{C}$) and quantitative descriptors $\:{NAF}_{fcc}$, $\:{NAF}_{bcc}$, $\:{NAF}_{hex}$, etc. (e.g., $\:{NAF}_{fcc}=43.2\text{\%}$) of the atomic fractions of the non-allotrope forming elements for each crystal structure are proposed. These descriptors and symbols provide a means for standardizing discussion of the role of allotropy and can be utilized in materials informatics applications. In the long term, further study of the incorrect predictions from this allotropy-based approach may shed new light on the physics governing crystal structures for HEAs and other classes of materials.

## Conclusions

The allotropy model for high entropy alloy crystal structure suggests that the non-allotrope elements play a significant and impactful role in the resultant crystal structure in concentrations as little as 5 atomic percent (e.g., the FCC alloy Ni_5_(CoFeMn)_95_). This provides a simple model for addressing two of the seminal questions in complex concentrated alloys: (1) will a single-phase form (therefore a high entropy alloy), and if so (2) which crystal structure will prevail. Future analyses of HEAs, including the allotropy descriptor, may shed further light on why some compositions cannot be explained solely by allotropy. Lastly, we present the challenge to the community to identify a single composition containing equal atomic percentages of two or more crystal structures, and only utilizing non-allotrope elements, that solidifies into a single crystal structure HEA.

## Supplementary Information

Below is the link to the electronic supplementary material.

Supplementary Material 1

## Data Availability

All data generated during and/or analyzed during the current study are available as part of the Main Text, the electronic Supplementary Material, or from the corresponding author upon reasonable request.

## References

[1] Yeh, J. W. et al. Nanostructured High-Entropy alloys with multiple principal elements: novel alloy design concepts and outcomes. *Adv. Eng. Mater.* **6**, 299–303. 10.1002/adem.200300567 (2004).

[2] Cantor, B., Chang, I. T. H., Knight, P. & Vincent, A. J. B. Microstructural development in equiatomic multicomponent alloys. *Mater. Sci. Eng. A* 375–377. 10.1016/J.MSEA.2003.10.257 (2004).

[3] Miracle, D. B. High entropy alloys as a bold step forward in alloy development. *Nat. Commun.* **10**, 1805. 10.1038/s41467-019-09700-1 (2019). 31000724 10.1038/s41467-019-09700-1 PMC6472357

[4] Dippo, O. F. & Vecchio, K. S. A universal configurational entropy metric for high-entropy materials. *Scr. Mater.* **201**, 113974. 10.1016/J.SCRIPTAMAT.2021.113974 (2021).

[5] Rost, C. M. et al. Entropy-stabilized oxides. *Nat. Commun.* **6**, 8485. 10.1038/ncomms9485 (2015). 26415623 10.1038/ncomms9485 PMC4598836

[6] McCormack, S. J. & Navrotsky, A. Thermodynamics of high entropy oxides. *Acta Mater.* **202**, 1–21. 10.1016/j.actamat.2020.10.043 (2021).

[7] Miracle, D. B. & Senkov, O. N. A critical review of high entropy alloys and related concepts. *Acta Mater.* **122**, 448–511. 10.1016/j.actamat.2016.08.081 (2017).

[8] Wu, Y. et al. Phase stability and mechanical properties of AlHfNbTiZr high-entropy alloys. *Mater. Sci. Eng. A*. **724**, 249–259. 10.1016/j.msea.2018.03.071 (2018).

[9] Miracle, D. et al. Exploration and development of high entropy alloys for structural applications. *Entropy* **16**, 494–525. 10.3390/e16010494 (2014).

[10] Sarker, P. et al. High-entropy high-hardness metal carbides discovered by entropy descriptors. *Nat. Commun.* **9**, 4980. 10.1038/s41467-018-07160-7 (2018). 30478375 10.1038/s41467-018-07160-7 PMC6255778

[11] Senkov, O. N., Miller, J. D., Miracle, D. B. & Woodward, C. Accelerated exploration of multi-principal element alloys with solid solution phases. *Nat. Commun.* **6**, 6529. 10.1038/ncomms7529 (2015). 25739749 10.1038/ncomms7529 PMC4366518

[12] Gild, J., Kaufmann, K., Vecchio, K. & Luo, J. Reactive flash spark plasma sintering of high-entropy ultrahigh temperature ceramics. *Scr. Mater.* **170**10.1016/j.scriptamat.2019.05.039 (2019).

[13] Gludovatz, B. et al. A fracture-resistant high-entropy alloy for cryogenic applications. *Sci. (80-)*. **345**, 1153–1158. 10.1126/science.1254581 (2014). 10.1126/science.1254581 25190791

[14] Harrington, T. J. et al. Phase stability and mechanical properties of novel high entropy transition metal carbides. *Acta Mater.* **166**, 271–280. 10.1016/j.actamat.2018.12.054 (2019).

[15] Lim, X. Mixed-up metals make for stronger, tougher, stretchier alloys. *Nature* **533**, 306–307. 10.1038/533306a (2016). 27193659 10.1038/533306a

[16] Li, Z., Tasan, C. C., Springer, H., Gault, B. & Raabe, D. Interstitial atoms enable joint twinning and transformation induced plasticity in strong and ductile high-entropy alloys. *Sci. Rep.* **7**, 40704. 10.1038/srep40704 (2017). 28079175 10.1038/srep40704 PMC5227964

[17] Tsao, T. K. et al. The high temperature tensile and creep behaviors of high entropy Superalloy. *Sci. Rep.* **7**, 12658. 10.1038/s41598-017-13026-7 (2017). 28978946 10.1038/s41598-017-13026-7 PMC5627260

[18] Senkov, O. N., Wilks, G. B., Scott, J. M. & Miracle, D. B. Mechanical properties of Nb25Mo25Ta25W25 and V20Nb20Mo20Ta20W20 refractory high entropy alloys. *Intermetallics* **19**, 698–706. 10.1016/J.INTERMET.2011.01.004 (2011).

[19] Li, Z., Pradeep, K. G., Deng, Y., Raabe, D. & Tasan, C. C. Metastable high-entropy dual-phase alloys overcome the strength–ductility trade-off. *Nature* **534**, 227–230. 10.1038/nature17981 (2016). 27279217 10.1038/nature17981

[20] von Rohr, F., Winiarski, M. J., Tao, J., Klimczuk, T. & Cava, R. J. Effect of electron count and chemical complexity in the Ta-Nb-Hf-Zr-Ti high-entropy alloy superconductor. *Proc. Natl. Acad. Sci. USA* **113** E7144–E7150 10.1073/pnas.1615926113 (2016). 10.1073/pnas.1615926113 PMC5135312 27803330

[21] Yeh, M. B. S. J. W. & Ranganathan, S. B. P. P. High-entropy alloys, second Volume, ASMT Handbook. 10.1016/B978-0-12-816067-1.00002-3 (2019).

[22] Gorsse, S., Nguyen, M. H., Senkov, O. N. & Miracle, D. B. Database on the mechanical properties of high entropy alloys and complex concentrated alloys. *Data Br.* **21**, 2664–2678. 10.1016/J.DIB.2018.11.111 (2018). 10.1016/j.dib.2018.11.111 PMC6290247 30761350

[23] Castle, E., Csanádi, T., Grasso, S., Dusza, J. & Reece, M. Processing and properties of High-Entropy Ultra-High temperature carbides. *Sci. Rep.* **8**, 8609. 10.1038/s41598-018-26827-1 (2018). 29872126 10.1038/s41598-018-26827-1 PMC5988827

[24] Mellor, W. M. et al. Development of ultrahigh-entropy ceramics with tailored oxidation behavior. *J. Eur. Ceram. Soc.* **41**, 5791–5800. 10.1016/J.JEURCERAMSOC.2021.05.010 (2021).

[25] Gild, J. et al. Thermal conductivity and hardness of three single-phase high-entropy metal diborides fabricated by borocarbothermal reduction and spark plasma sintering. *Ceram. Int.* **46**, 6906–6913. 10.1016/j.ceramint.2019.11.186 (2020).

[26] Gild, J. et al. A high-entropy silicide: (Mo0.2Nb0.2Ta0.2Ti0.2W0.2)Si2. *J. Mater.* **5**, 337–343. 10.1016/j.jmat.2019.03.002 (2019).

[27] Wright, A. J. et al. Short-range order and origin of the low thermal conductivity in compositionally complex rare-earth niobates and tantalates. *Acta Mater.* **235**, 118056. 10.1016/j.actamat.2022.118056 (2022).

[28] GUO, S. & LIU, C. T. Phase stability in high entropy alloys: formation of solid-solution phase or amorphous phase. *Prog Nat. Sci. Mater. Int.* **21**, 433–446. 10.1016/S1002-0071(12)60080-X (2011).

[29] Murty, B. S., Yeh, J. W., Ranganathan, S. & Bhattacharjee, P. *High-entropy Alloys* 2nd edn (Elsevier, 2019).

[30] Miracle, D., Majumdar, B., Wertz, K. & Gorsse, S. New strategies and tests to accelerate discovery and development of multi-principal element structural alloys. *Scr. Mater.* **127**, 195–200. 10.1016/j.scriptamat.2016.08.001 (2017).

[31] Gao, M. C. Design of high-entropy alloys, in: High-Entropy alloy. *Fundam. Appl. Springer Int. Publish.* 369–398. 10.1007/978-3-319-27013-5_11 (2016).

[32] Senkov, O. N., Wilks, G. B., Miracle, D. B., Chuang, C. P. & Liaw, P. K. Refractory high-entropy alloys. *Intermetallics* **18**, 1758–1765. 10.1016/j.intermet.2010.05.014 (2010).

[33] Wang, F., Zhang, Y., Chen, G., Davies, H. A. Tensile and compressive mechanical behavior of a CoCrCuFeNiAl0.5 high entropy alloy. **23**, 1254–1259 10.1142/S0217979209060774 (2012).

[34] Huang, H., Shao, L. & Liu, H. Prediction of Single-Phase High-Entropy nitrides from First-Principles calculations. *Phys. Status Solidi*. **258**, 2100140. 10.1002/PSSB.202100140 (2021).

[35] Lederer, Y., Toher, C., Vecchio, K. S. & Curtarolo, S. The search for high entropy alloys: A high-throughput ab-initio approach. *Acta Mater.* **159**, 364–383. 10.1016/j.actamat.2018.07.042 (2018).

[36] Gao, M. C., Yeh, J. W., Liaw, P. K. & Zhang, Y. *High-Entropy Alloys*10.1007/978-3-319-27013-5 (Springer International Publishing, 2016).

[37] Feng, R., Liaw, P. K., Gao, M. C. & Widom, M. First-principles prediction of high-entropy-alloy stability. *Npj Comput. Mater.* **3**10.1038/s41524-017-0049-4 (2017).

[38] Cheney, J. & Vecchio, K. Evaluation of glass-forming ability in metals using multi-model techniques. *J. Alloys Compd.* **471**, 222–240. 10.1016/J.JALLCOM.2008.03.071 (2009).

[39] Cheney, J. Utilizing big data informatics for thermal spray materials design. In *Proc. Int. Therm. Spray Conf.* 430–435. 10.31399/ASM.CP.ITSC2018P0430/23814/UTILIZING-BIG-DATA-INFORMATICS-FOR-THERMAL-SPRAY (2018).

[40] Liu, Z. K. Computational thermodynamics and its applications. *Acta Mater.* **200**, 745–792. 10.1016/J.ACTAMAT.2020.08.008 (2020).

[41] Yi Wang, W., Li, J., Liu, W. & Liu, Z. K. Integrated computational materials engineering for advanced materials: A brief review. *Comput. Mater. Sci.* **158**, 42–48. 10.1016/J.COMMATSCI.2018.11.001 (2019).

[42] Agarwal, A. & Rao, A. K. P. Artificial intelligence predicts Body-Centered-Cubic and Face-Centered-Cubic phases in High-Entropy alloys. *JOM* **71**, 3424–3432. 10.1007/s11837-019-03712-4 (2019).

[43] Kaufmann, K. & Vecchio, K. S. Searching for high entropy alloys: A machine learning approach. *Acta Mater.* **198**, 178–222. 10.1016/j.actamat.2020.07.065 (2020).

[44] Huang, W., Martin, P. & Zhuang, H. L. Machine-learning phase prediction of high-entropy alloys. *Acta Mater.* **169**, 225–236. 10.1016/j.actamat.2019.03.012 (2019).

[45] Vazquez, G., Chakravarty, S., Gurrola, R. & Arróyave, R. A deep neural network regressor for phase constitution estimation in the high entropy alloy system Al-Co-Cr-Fe-Mn-Nb-Ni. *Npj Comput. Mater.* **91** (9), 1–14 10.1038/s41524-023-01021-8 (2023).

[46] Kaufmann, K. et al. Discovery of high-entropy ceramics via machine learning. *Npj Comput. Mater.* **6**, 42. 10.1038/s41524-020-0317-6 (2020).

[47] Liu, Z. K. Ocean of data: integrating First-Principles calculations and CALPHAD modeling with machine learning. *J. Phase Equilib. Diffus.* **39**, 635–649. 10.1007/s11669-018-0654-z (2018).

[48] Ye, Y. F., Wang, Q., Lu, J., Liu, C. T. & Yang, Y. Design of high entropy alloys: A single-parameter thermodynamic rule. *Scr. Mater.* **104**, 53–55. 10.1016/j.scriptamat.2015.03.023 (2015).

[49] Zhang, Y., Zhou, Y. J., Lin, J. P., Chen, G. L. & Liaw, P. K. Solid-Solution phase formation rules for Multi-component alloys. *Adv. Eng. Mater.* **10**, 534–538. 10.1002/adem.200700240 (2008).

[50] Poletti, M. G. & Battezzati, L. Electronic and thermodynamic criteria for the occurrence of high entropy alloys in metallic systems. *Acta Mater.* **75**, 297–306. 10.1016/J.ACTAMAT.2014.04.033 (2014).

[51] Ye, Y. F., Liu, C. T. & Yang, Y. A geometric model for intrinsic residual strain and phase stability in high entropy alloys. *Acta Mater.* **94**, 152–161. 10.1016/J.ACTAMAT.2015.04.051 (2015).

[52] Yang, X., Chen, S. Y., Cotton, J. D. & Zhang, Y. Phase stability of Low-Density, multiprincipal component alloys containing aluminum, magnesium, and lithium. *JOM* **66**, 2009–2020. 10.1007/S11837-014-1059-Z/FIGURES/6 (2014).

[53] Hume-Rothery, W. *Atomic Theory for Students of Metallurgy* (Institute of Metals, 1952).

[54] The pandas development team, pandas-dev/pandas: Pandas (v2.2.0). 10.5281/zenodo.3509134 (2024).

[55] Andersson, J. O., Helander, T., Höglund, L., Shi, P. & Sundman, B. Thermo-Calc & DICTRA, computational tools for materials science. *Calphad Comput. Coupling Phase Diagrams Thermochem*. **26**, 273–312. 10.1016/S0364-5916(02)00037-8 (2002).

[56] Spendlove, J. C. et al. Composition-based phase stability model for multicomponent metal alloys. *AIP Adv.* **14**, 15342. 10.1063/5.0182293/3138264 (2023).

[57] Troparevsky, M. C., Morris, J. R., Kent, P. R. C., Lupini, A. R. & Stocks, G. M. Criteria for predicting the formation of single-phase high-entropy alloys. *Phys. Rev. X*. **5**, 011041. 10.1103/PHYSREVX.5.011041/FIGURES/2/MEDIUM (2015).

[58] Pei, Z., Yin, J., Hawk, J. A., Alman, D. E. & Gao, M. C. Machine-learning informed prediction of high-entropy solid solution formation: beyond the Hume-Rothery rules. *Npj Comput. Mater. 2020*. **61** (6), 1–8. 10.1038/s41524-020-0308-7 (2020).

[59] Wang, C., Zhong, W. & Zhao, J. C. Insights on phase formation from thermodynamic calculations and machine learning of 2436 experimentally measured high entropy alloys. *J. Alloys Compd.* **915**, 165173. 10.1016/J.JALLCOM.2022.165173 (2022).

[60] Guo, S., Ng, C., Lu, J. & Liu, C. T. Effect of Valence electron concentration on stability of Fcc or Bcc phase in high entropy alloys. *J. Appl. Phys.*10.1063/1.3587228 (2011).

[61] Kurniawan, M., Perrin, A., Xu, P., Keylin, V. & McHenry, M. Curie temperature engineering in high entropy alloys for magnetocaloric applications. *IEEE Magn. Lett.* **7**10.1109/LMAG.2016.2592462 (2016).

[62] Lucas, M. S. et al. Magnetic and vibrational properties of high-entropy alloys. *J. Appl. Phys.* **109**10.1063/1.3538936/370378 (2011).

[63] Youssef, K. M., Zaddach, A. J., Niu, C., Irving, D. L. & Koch, C. C. A novel Low-Density, High-Hardness, High-entropy alloy with Close-packed Single-phase nanocrystalline structures. *Mater. Res. Lett.* **3**, 95–99. 10.1080/21663831.2014.985855 (2015).

[64] Dwivedi, A., Koch, C. C. & Rajulapati, K. V. On the single phase Fcc solid solution in nanocrystalline Cr-Nb-Ti-V-Zn high-entropy alloy. *Mater. Lett.* **183**, 44–47. 10.1016/J.MATLET.2016.07.083 (2016).

