# Supplementary material for: The role of allotropy on phase formation in high entropy alloys
Source: Sci Rep. 2025 Aug 26;15:31341. doi: 10.1038/s41598-025-17217-5 (PMC12381287; doi:10.1038/s41598-025-17217-5)
Supplement: Supplementary file 1 — Supplementary Material 1 [file 41598_2025_17217_MOESM1_ESM.docx]

**The Role of Allotropy on Phase Formation in High Entropy Alloys**

Kevin Kaufmann^a,b^, Haoren Wang^a^, Jaskaran Saini^a^, and Kenneth S. Vecchio^a*^

^a^Department of NanoEngineering, UC San Diego, La Jolla, CA 92093, USA

^b^Materials R&D, Oerlikon Metco (US) Inc., San Diego, CA 92127, United States

*Corresponding author, e-mail: kvecchio@ucsd.edu

**Supplementary Table 1. Analysis of HEAs from Gorsse *et al*.** The atomic percent of each composition that is not elements exhibiting allotropism is detailed. This information is subdivided into the atom percent of FCC, BCC, and hex non-allotropes present, which can be compared to the known phase. The columns equal FCC and BCC, more FCC – crystal BCC, and more BCC – crystal FCC are Boolean (i.e., 1 for True). Lastly the aluminum content in the alloy is reported as a separate column. Alloys are in descending order by the percentage of non-allotrope elements column.

| Composition  (atom %) | Phase | Non-Allotropes  (atom %) | FCC  (atom %) | BCC  (atom %) | HEX  (atom %) | Equal  FCC & BCC | More FCC –  Crystal BCC | More BCC –Crystal FCC | Al  (atom %) |
| --- | --- | --- | --- | --- | --- | --- | --- | --- | --- |
| MoNbTaV | BCC | 100 | 0.0 | 100.0 | 0.0 | 0 | 0 | 0 | 0 |
| MoNbTaVW | BCC | 100 | 0.0 | 100.0 | 0.0 | 0 | 0 | 0 | 0 |
| MoNbTaW | BCC | 100 | 0.0 | 100.0 | 0.0 | 0 | 0 | 0 | 0 |
| NbTaVW | BCC | 100 | 0.0 | 100.0 | 0.0 | 0 | 0 | 0 | 0 |
| AlCu0.2Li0.5MgZn0.5 | Im | 84.38 | 37.5 | 0.0 | 46.9 | 0 | 0 | 0 | 31.25 |
| Al0.8CrCuFeNi2 | FCC | 82.76 | 65.5 | 17.2 | 0.0 | 0 | 0 | 0 | 13.79 |
| Al0.6CrCuFeNi2 | FCC | 82.14 | 64.3 | 17.9 | 0.0 | 0 | 0 | 0 | 10.71 |
| Al0.4CrCuFeNi2 | FCC | 81.48 | 63.0 | 18.5 | 0.0 | 0 | 0 | 0 | 7.41 |
| Al0.2CrCuFeNi2 | FCC | 80.77 | 61.5 | 19.2 | 0.0 | 0 | 0 | 0 | 3.85 |
| CrCuFeMoNi | FCC | 80 | 40.0 | 40.0 | 0.0 | 1 | 0 | 0 | 0 |
| AlMoNbTiV | BCC | 80 | 20.0 | 60.0 | 0.0 | 0 | 0 | 0 | 20 |
| AlNbTaTiV | BCC | 80 | 20.0 | 60.0 | 0.0 | 0 | 0 | 0 | 20 |
| Al0.75MoNbTiV | BCC | 78.95 | 15.8 | 63.2 | 0.0 | 0 | 0 | 0 | 15.79 |
| Al22.5Cu20Fe15Ni20Ti2 | FCC | 78.62 | 78.6 | 0.0 | 0.0 | 0 | 0 | 0 | 28.3 |
| AlCu0.5Li0.5MgSn0.2 | Im | 78.12 | 46.9 | 0.0 | 31.3 | 0 | 0 | 0 | 31.25 |
| Al0.5MoNbTiV | BCC | 77.78 | 11.1 | 66.7 | 0.0 | 0 | 0 | 0 | 11.11 |
| Al0.5NbTaTiV | BCC | 77.78 | 11.1 | 66.7 | 0.0 | 0 | 0 | 0 | 11.11 |
| AlCrFeNiMo0.5 | BCC | 77.78 | 44.4 | 33.3 | 0.0 | 0 | 1 | 0 | 22.22 |
| AlCr0.5NbTiV | BCC | 77.78 | 22.2 | 55.6 | 0.0 | 0 | 0 | 0 | 22.22 |
| Al0.25MoNbTiV | BCC | 76.47 | 5.9 | 70.6 | 0.0 | 0 | 0 | 0 | 5.88 |
| Al0.25NbTaTiV | BCC | 76.47 | 5.9 | 70.6 | 0.0 | 0 | 0 | 0 | 5.88 |
| AlCrFeNiMo0.2 | BCC | 76.19 | 47.6 | 28.6 | 0.0 | 0 | 1 | 0 | 23.81 |
| MoNbTiV | BCC | 75 | 0.0 | 75.0 | 0.0 | 0 | 0 | 0 | 0 |
| NbTaTiV | BCC | 75 | 0.0 | 75.0 | 0.0 | 0 | 0 | 0 | 0 |
| Zn25(CuMnNi)75 | FCC | 75 | 50.0 | 0.0 | 25.0 | 0 | 0 | 0 | 0 |
| AlCrFeNi | BCC | 75 | 50.0 | 25.0 | 0.0 | 0 | 1 | 0 | 25 |
| AlCuNiTi | FCC | 75 | 75.0 | 0.0 | 0.0 | 0 | 0 | 0 | 25 |
| Al3CoCrCuFeNi | BCC | 75 | 62.5 | 12.5 | 0.0 | 0 | 1 | 0 | 37.5 |
| Al2.8CoCrCuFeNi | BCC | 74.36 | 61.5 | 12.8 | 0.0 | 0 | 1 | 0 | 35.9 |
| NbTiV0.3Mo1.5 | BCC | 73.68 | 0.0 | 73.7 | 0.0 | 0 | 0 | 0 | 0 |
| Zn20(CuMnNi)80 | FCC | 73.33 | 53.3 | 0.0 | 20.0 | 0 | 0 | 0 | 0 |
| Al0.5CoCrCuFeNiV2.0 | BCC | 73.33 | 33.3 | 40.0 | 0.0 | 0 | 0 | 0 | 6.67 |
| Al0.5CoCrCuFeNiV1.8 | BCC | 72.6 | 34.2 | 38.4 | 0.0 | 0 | 0 | 0 | 6.85 |
| NbTiV0.3Mo1.3 | BCC | 72.22 | 0.0 | 72.2 | 0.0 | 0 | 0 | 0 | 0 |
| Al0.5CoCrCuFeNiV1.6 | BCC | 71.83 | 35.2 | 36.6 | 0.0 | 0 | 0 | 0 | 7.04 |
| MoNbTiV3.0Zr | BCC | 71.43 | 0.0 | 71.4 | 0.0 | 0 | 0 | 0 | 0 |
| Al3CoCrFeNi | BCC | 71.43 | 57.1 | 14.3 | 0.0 | 0 | 1 | 0 | 42.86 |
| Al3.0CoCrCuFe | BCC | 71.43 | 57.1 | 14.3 | 0.0 | 0 | 1 | 0 | 42.86 |
| Al0.5CoCrCuFeNiV1.4 | BCC | 71.01 | 36.2 | 34.8 | 0.0 | 0 | 1 | 0 | 7.25 |
| Al2.8CoCrCuFe | BCC | 70.59 | 55.9 | 14.7 | 0.0 | 0 | 1 | 0 | 41.18 |
| Al0.5CoCrCuFeNiV1.2 | BCC | 70.15 | 37.3 | 32.8 | 0.0 | 0 | 1 | 0 | 7.46 |
| NbTiV0.3Mo | BCC | 69.7 | 0.0 | 69.7 | 0.0 | 0 | 0 | 0 | 0 |
| Al2CoCrFeMo0.5Ni | BCC | 69.23 | 46.2 | 23.1 | 0.0 | 0 | 1 | 0 | 30.77 |
| Al2.5CoCrFeNi | BCC | 69.23 | 53.8 | 15.4 | 0.0 | 0 | 1 | 0 | 38.46 |
| Al5(CuMnNi)95 | FCC | 68.33 | 68.3 | 0.0 | 0.0 | 0 | 0 | 0 | 5 |
| CoCrNi | FCC | 66.67 | 33.3 | 33.3 | 0.0 | 1 | 0 | 0 | 0 |
| Mo2NbTiVZr | BCC | 66.67 | 0.0 | 66.7 | 0.0 | 0 | 0 | 0 | 0 |
| MoNbTiV2.0Zr | BCC | 66.67 | 0.0 | 66.7 | 0.0 | 0 | 0 | 0 | 0 |
| NbTiV0.3Mo0.7 | BCC | 66.67 | 0.0 | 66.7 | 0.0 | 0 | 0 | 0 | 0 |
| AlCoCrCuNiTi | BCC | 66.67 | 50.0 | 16.7 | 0.0 | 0 | 1 | 0 | 16.67 |
| AlCoCuFeNbNi | Im | 66.67 | 50.0 | 16.7 | 0.0 | 0 | 0 | 0 | 16.67 |
| Al2CoCrFeNi | BCC | 66.67 | 50.0 | 16.7 | 0.0 | 0 | 1 | 0 | 33.33 |
| Mo1.7NbTiVZr | BCC | 64.91 | 0.0 | 64.9 | 0.0 | 0 | 0 | 0 | 0 |
| Al0.5CoCrCuFeNiV0.2 | FCC | 64.91 | 43.9 | 21.1 | 0.0 | 0 | 0 | 0 | 8.77 |
| NbTiV0.3Mo0.5 | BCC | 64.29 | 0.0 | 64.3 | 0.0 | 0 | 0 | 0 | 0 |
| Mo1.5NbTiVZr | BCC | 63.64 | 0.0 | 63.6 | 0.0 | 0 | 0 | 0 | 0 |
| MoNbTiV1.5Zr | BCC | 63.64 | 0.0 | 63.6 | 0.0 | 0 | 0 | 0 | 0 |
| Al0.5CoCrCuFeNi | FCC | 63.64 | 45.5 | 18.2 | 0.0 | 0 | 0 | 0 | 9.09 |
| Al1.5CoCrFeNi | BCC | 63.64 | 45.5 | 18.2 | 0.0 | 0 | 1 | 0 | 27.27 |
| Al1.125CuFe0.75NiTi1.1 | FCC | 62.81 | 62.8 | 0.0 | 0.0 | 0 | 0 | 0 | 22.61 |
| Mo1.3NbTiVZr | BCC | 62.26 | 0.0 | 62.3 | 0.0 | 0 | 0 | 0 | 0 |
| Al0.3CoCrCuFeNi | FCC | 62.26 | 43.4 | 18.9 | 0.0 | 0 | 0 | 0 | 5.66 |
| Al1.25CoCrFeNi | BCC | 61.9 | 42.9 | 19.0 | 0.0 | 0 | 1 | 0 | 23.81 |
| NbTiV0.3Mo0.3 | BCC | 61.54 | 0.0 | 61.5 | 0.0 | 0 | 0 | 0 | 0 |
| AlCoCrCuNiTiY0.5 | Im | 61.54 | 46.2 | 15.4 | 0.0 | 0 | 0 | 0 | 15.38 |
| Al0.5B0.2CoCrCuFeNi |  | 61.4 | 43.9 | 17.5 | 0.0 | 0 | 0 | 0 | 8.77 |
| Al0.5CoCrCuFeNiTi0.2 | FCC | 61.4 | 43.9 | 17.5 | 0.0 | 0 | 0 | 0 | 8.77 |
| AlCoCrFeMo0.1Ni | BCC | 60.78 | 39.2 | 21.6 | 0.0 | 0 | 1 | 0 | 19.61 |
| AlCoCrFeNb0.1Ni | BCC | 60.78 | 39.2 | 21.6 | 0.0 | 0 | 1 | 0 | 19.61 |
| CoCrCuFeNi | FCC | 60 | 40.0 | 20.0 | 0.0 | 0 | 0 | 0 | 0 |
| MoNbTiVZr | BCC | 60 | 0.0 | 60.0 | 0.0 | 0 | 0 | 0 | 0 |
| MoNbTiV1.0Zr | BCC | 60 | 0.0 | 60.0 | 0.0 | 0 | 0 | 0 | 0 |
| NbTiV2Zr | BCC | 60 | 0.0 | 60.0 | 0.0 | 0 | 0 | 0 | 0 |
| AlCoCrFeNi | BCC | 60 | 40.0 | 20.0 | 0.0 | 0 | 1 | 0 | 20 |
| AlCuFeNiTi | FCC | 60 | 60.0 | 0.0 | 0.0 | 0 | 0 | 0 | 20 |
| AlMo0.5NbTa0.5TiZr | BCC | 60 | 20.0 | 40.0 | 0.0 | 0 | 0 | 0 | 20 |
| AlNb1.5Ta0.5Ti1.5Zr0.5 | BCC | 60 | 20.0 | 40.0 | 0.0 | 0 | 0 | 0 | 20 |
| AlNBTiV | BCC | 60 | 20.0 | 20.0 | 0.0 | 1 | 0 | 0 | 20 |
| Al20(CoCrCuFeMnNiTiV)80 | BCC | 60 | 40.0 | 20.0 | 0.0 | 0 | 1 | 0 | 20 |
| Al0.5CoCrCuFeNiTi0.4 | FCC | 59.32 | 42.4 | 16.9 | 0.0 | 0 | 0 | 0 | 8.47 |
| AlCoCrCuNiTiY0.8 | Im | 58.82 | 44.1 | 14.7 | 0.0 | 0 | 0 | 0 | 14.71 |
| NbTiV0.3Mo0.1 | BCC | 58.33 | 0.0 | 58.3 | 0.0 | 0 | 0 | 0 | 0 |
| MoNbTiV0.75Zr | BCC | 57.89 | 0.0 | 57.9 | 0.0 | 0 | 0 | 0 | 0 |
| AlCoCrFeNiSi0.2 | BCC | 57.69 | 38.5 | 19.2 | 0.0 | 0 | 1 | 0 | 19.23 |
| Mo0.7NbTiVZr | BCC | 57.45 | 0.0 | 57.4 | 0.0 | 0 | 0 | 0 | 0 |
| Al0.5B0.6CoCrCuFeNi |  | 57.38 | 41.0 | 16.4 | 0.0 | 0 | 0 | 0 | 8.2 |
| AlCoCrCuNiTiY | Im | 57.14 | 42.9 | 14.3 | 0.0 | 0 | 0 | 0 | 14.29 |
| Al2CoCrFeNiTi | BCC | 57.14 | 42.9 | 14.3 | 0.0 | 0 | 1 | 0 | 28.57 |
| CoCrCu0.5FeNi | FCC | 55.56 | 33.3 | 22.2 | 0.0 | 0 | 0 | 0 | 0 |
| Mo0.5NbTiVZr | BCC | 55.56 | 0.0 | 55.6 | 0.0 | 0 | 0 | 0 | 0 |
| MoNbTiV0.50Zr | BCC | 55.56 | 0.0 | 55.6 | 0.0 | 0 | 0 | 0 | 0 |
| Al0.5CoCrCuFe | FCC | 55.56 | 33.3 | 22.2 | 0.0 | 0 | 0 | 0 | 11.11 |
| AlCoCrFeNiSi0.4 | BCC | 55.56 | 37.0 | 18.5 | 0.0 | 0 | 1 | 0 | 18.52 |
| CoCrCuFeNiTi0.5 | FCC | 54.55 | 36.4 | 18.2 | 0.0 | 0 | 0 | 0 | 0 |
| AlCoCrFeNiTi0.5 | FCC | 54.55 | 36.4 | 18.2 | 0.0 | 0 | 0 | 0 | 18.18 |
| Al0.375CoCrFeNi | FCC | 54.29 | 31.4 | 22.9 | 0.0 | 0 | 0 | 0 | 8.57 |
| Al0.5BCoCrCuFeNi |  | 53.85 | 38.5 | 15.4 | 0.0 | 0 | 0 | 0 | 7.69 |
| Al1.5CoCrFeNiTi | BCC | 53.85 | 38.5 | 15.4 | 0.0 | 0 | 1 | 0 | 23.08 |
| AlCoCrFeNiSi0.6 | BCC | 53.57 | 35.7 | 17.9 | 0.0 | 0 | 1 | 0 | 17.86 |
| Mo0.3NbTiVZr | BCC | 53.49 | 0.0 | 53.5 | 0.0 | 0 | 0 | 0 | 0 |
| Al0.3CoCrCuFe | FCC | 53.49 | 30.2 | 23.3 | 0.0 | 0 | 0 | 0 | 6.98 |
| MoNbTiV0.25Zr | BCC | 52.94 | 0.0 | 52.9 | 0.0 | 0 | 0 | 0 | 0 |
| Al0.25CoCrFeNi | FCC | 52.94 | 29.4 | 23.5 | 0.0 | 0 | 0 | 0 | 5.88 |
| Al1.25CoCrFeMnNi | BCC | 52 | 36.0 | 16.0 | 0.0 | 0 | 1 | 0 | 20 |
| CoCrFeNi | FCC | 50 | 25.0 | 25.0 | 0.0 | 1 | 0 | 0 | 0 |
| CoCrMnNi | FCC | 50 | 25.0 | 25.0 | 0.0 | 1 | 0 | 0 | 0 |
| CoCrCuFe | FCC | 50 | 25.0 | 25.0 | 0.0 | 1 | 0 | 0 | 0 |
| CoCrCuFeNiTi | FCC | 50 | 33.3 | 16.7 | 0.0 | 0 | 0 | 0 | 0 |
| CoCuFeNi | FCC | 50 | 50.0 | 0.0 | 0.0 | 0 | 0 | 0 | 0 |
| HfMoNbTaTiZr | BCC | 50 | 0.0 | 50.0 | 0.0 | 0 | 0 | 0 | 0 |
| HfNbTaZr | BCC | 50 | 0.0 | 50.0 | 0.0 | 0 | 0 | 0 | 0 |
| MoNbTiZr | BCC | 50 | 0.0 | 50.0 | 0.0 | 0 | 0 | 0 | 0 |
| NbTiVZr | BCC | 50 | 0.0 | 50.0 | 0.0 | 0 | 0 | 0 | 0 |
| CoCrCuFeNiTiVZr | FCC | 50 | 25.0 | 25.0 | 0.0 | 1 | 0 | 0 | 0 |
| CoCrFeMoNiTiVZr |  | 50 | 12.5 | 37.5 | 0.0 | 0 | 0 | 0 | 0 |
| CoFeNiV | FCC | 50 | 25.0 | 25.0 | 0.0 | 1 | 0 | 0 | 0 |
| CuFeNiTiVZr |  | 50 | 33.3 | 16.7 | 0.0 | 0 | 0 | 0 | 0 |
| Al0.25CoCrCu0.75FeNiTi | FCC | 50 | 33.3 | 16.7 | 0.0 | 0 | 0 | 0 | 4.17 |
| Al0.5NbTa0.8Ti1.5V0.2Zr | BCC | 50 | 10.0 | 40.0 | 0.0 | 0 | 0 | 0 | 10 |
| AlCoCrFeNiSi | BCC | 50 | 33.3 | 16.7 | 0.0 | 0 | 1 | 0 | 16.67 |
| AlCoCrFeNiTi | BCC | 50 | 33.3 | 16.7 | 0.0 | 0 | 1 | 0 | 16.67 |
| AlFeNiTiVZr | BCC | 50 | 33.3 | 16.7 | 0.0 | 0 | 1 | 0 | 16.67 |
| AlCoFeNi | BCC | 50 | 50.0 | 0.0 | 0.0 | 0 | 1 | 0 | 25 |
| CoCuFeNiSn0.02 | FCC | 49.75 | 49.8 | 0.0 | 0.0 | 0 | 0 | 0 | 0 |
| Al0.4Hf0.6NbTaTiZr | BCC | 48 | 8.0 | 40.0 | 0.0 | 0 | 0 | 0 | 8 |
| HfMo0.75NbTaTiZr | BCC | 47.83 | 0.0 | 47.8 | 0.0 | 0 | 0 | 0 | 0 |
| Al0.25CoCrCu0.5FeNiTi | FCC | 47.83 | 30.4 | 17.4 | 0.0 | 0 | 0 | 0 | 4.35 |
| Al0.75HfNbTaTiZr | BCC | 47.83 | 13.0 | 34.8 | 0.0 | 0 | 0 | 0 | 13.04 |
| Al0.2Co1.5CrFeNi1.5Ti0.5 | FCC | 47.37 | 29.8 | 17.5 | 0.0 | 0 | 0 | 0 | 3.51 |
| Al0.3NbTa0.8Ti1.4V0.2Zr1.3 | BCC | 46 | 6.0 | 40.0 | 0.0 | 0 | 0 | 0 | 6 |
| Al0.3NbTaTi1.4Zr1.3 | BCC | 46 | 6.0 | 40.0 | 0.0 | 0 | 0 | 0 | 6 |
| Co1.5CrFeNi1.5Ti0.5 | FCC | 45.45 | 27.3 | 18.2 | 0.0 | 0 | 0 | 0 | 0 |
| CoCrFeMnNiV0.5 | FCC | 45.45 | 18.2 | 27.3 | 0.0 | 0 | 0 | 1 | 0 |
| HfMo0.5NbTaTiZr | BCC | 45.45 | 0.0 | 45.5 | 0.0 | 0 | 0 | 0 | 0 |
| Al0.5HfNbTaTiZr | BCC | 45.45 | 9.1 | 36.4 | 0.0 | 0 | 0 | 0 | 9.09 |
| Al0.5CrFe1.5MnNi0.5 | BCC | 44.44 | 22.2 | 22.2 | 0.0 | 1 | 0 | 0 | 11.11 |
| Al0.38CoCrFeMnNi | FCC | 44.24 | 25.7 | 18.6 | 0.0 | 0 | 0 | 0 | 7.06 |
| Al0.3HfNbTaTiZr | BCC | 43.4 | 5.7 | 37.7 | 0.0 | 0 | 0 | 0 | 5.66 |
| CoCrFeMnNiV0.25 | FCC | 42.86 | 19.0 | 23.8 | 0.0 | 0 | 0 | 1 | 0 |
| HfMo0.25NbTaTiZr | BCC | 42.86 | 0.0 | 42.9 | 0.0 | 0 | 0 | 0 | 0 |
| CoCuFeNiTiVZr |  | 42.86 | 28.6 | 14.3 | 0.0 | 0 | 0 | 0 | 0 |
| CoFeMoNiTiVZr |  | 42.86 | 14.3 | 28.6 | 0.0 | 0 | 0 | 0 | 0 |
| AlCoFeNiTiVZr | BCC | 42.86 | 28.6 | 14.3 | 0.0 | 0 | 1 | 0 | 14.29 |
| Al0.20CoCrFeMnNi | FCC | 42.31 | 23.1 | 19.2 | 0.0 | 0 | 0 | 0 | 3.85 |
| Al0.10CoCrFeMnNi | FCC | 41.18 | 21.6 | 19.6 | 0.0 | 0 | 0 | 0 | 1.96 |
| CoCrFeNiTi | FCC | 40 | 20.0 | 20.0 | 0.0 | 1 | 0 | 0 | 0 |
| CoCrFeMnNi | FCC | 40 | 20.0 | 20.0 | 0.0 | 1 | 0 | 0 | 0 |
| CoCuFeMnNi | FCC | 40 | 40.0 | 0.0 | 0.0 | 0 | 0 | 0 | 0 |
| HfMoTaTiZr | BCC | 40 | 0.0 | 40.0 | 0.0 | 0 | 0 | 0 | 0 |
| HfMoNbZrTi | BCC | 40 | 0.0 | 40.0 | 0.0 | 0 | 0 | 0 | 0 |
| HfNbTaTiZr | BCC | 40 | 0.0 | 40.0 | 0.0 | 0 | 0 | 0 | 0 |
| CoCuFeMnNiSn0.03 | FCC | 39.76 | 39.8 | 0.0 | 0.0 | 0 | 0 | 0 | 0 |
| NbTiV0.3Zr | BCC | 39.39 | 0.0 | 39.4 | 0.0 | 0 | 0 | 0 | 0 |
| Al0.25CoFeNi | FCC | 38.46 | 38.5 | 0.0 | 0.0 | 0 | 0 | 0 | 7.69 |
| CoFeNi | FCC | 33.33 | 33.3 | 0.0 | 0.0 | 0 | 0 | 0 | 0 |
| CoMnNi | FCC | 33.33 | 33.3 | 0.0 | 0.0 | 0 | 0 | 0 | 0 |
| FeMnNi | FCC | 33.33 | 33.3 | 0.0 | 0.0 | 0 | 0 | 0 | 0 |
| CoFeNiSi0.25 | FCC | 30.77 | 30.8 | 0.0 | 0.0 | 0 | 0 | 0 | 0 |
| CoFeMnNi | FCC | 25 | 25.0 | 0.0 | 0.0 | 0 | 0 | 0 | 0 |
| Hf0.5Nb0.5Ta0.5Ti1.5Zr | BCC | 25 | 0.0 | 25.0 | 0.0 | 0 | 0 | 0 | 0 |
| HfNbTiZr | BCC | 25 | 0.0 | 25.0 | 0.0 | 0 | 0 | 0 | 0 |

**Supplementary Table 2. Analysis of HEAs from Murty *et al*.** The atomic percent of each composition that is not elements exhibiting allotropism is detailed. This information is subdivided into the atom percent of FCC, BCC, and hex non-allotropes present, which can be compared to the known phase. The columns equal FCC and BCC, more FCC – crystal BCC, and more BCC – crystal FCC are Boolean (i.e., 1 for True). Lastly the aluminum content in the alloy is reported as a separate column. Alloys are in descending order by the percentage of non-allotrope elements column.

| Composition  (atom %) | Phase | Non-Allotropes  (atom %) | FCC  (atom %) | BCC  (atom %) | HEX  (atom %) | Equal  FCC & BCC | More FCC –  Crystal BCC | More BCC –  Crystal FCC | Al  (atom %) |
| --- | --- | --- | --- | --- | --- | --- | --- | --- | --- |
| MoNbTaW | BCC | 100.0 | 0.0 | 100.0 | 0.0 | 0 | 0 | 0 | 0 |
| NbTaVW | BCC | 100.0 | 0.0 | 100.0 | 0.0 | 0 | 0 | 0 | 0 |
| MoNbTaVW | BCC | 100.0 | 0.0 | 100.0 | 0.0 | 0 | 0 | 0 | 0 |
| CrMoNbReTaVW | BCC | 100.0 | 0.0 | 85.7 | 14.3 | 0 | 0 | 0 | 0 |
| Cr0.5MoNbTaVW | BCC | 100.0 | 0.0 | 100.0 | 0.0 | 0 | 0 | 0 | 0 |
| CrMoNbTaVW | BCC | 100.0 | 0.0 | 100.0 | 0.0 | 0 | 0 | 0 | 0 |
| AgAuPdPt | FCC | 100.0 | 100.0 | 0.0 | 0.0 | 0 | 0 | 0 | 0 |
| AuCuNiPd | FCC | 100.0 | 100.0 | 0.0 | 0.0 | 0 | 0 | 0 | 0 |
| AuCuNiPt | FCC | 100.0 | 100.0 | 0.0 | 0.0 | 0 | 0 | 0 | 0 |
| AuCuPdPt | FCC | 100.0 | 100.0 | 0.0 | 0.0 | 0 | 0 | 0 | 0 |
| AuNiPdPt | FCC | 100.0 | 100.0 | 0.0 | 0.0 | 0 | 0 | 0 | 0 |
| CuNiPdPt | FCC | 100.0 | 100.0 | 0.0 | 0.0 | 0 | 0 | 0 | 0 |
| AuCuNiPdPt | FCC | 100.0 | 100.0 | 0.0 | 0.0 | 0 | 0 | 0 | 0 |
| CuIrNiPdPtRh | FCC | 100.0 | 100.0 | 0.0 | 0.0 | 0 | 0 | 0 | 0 |
| Ir0.26Os0.05Pt0.31Rh0.23Ru0.15 | FCC | 100.0 | 80.0 | 0.0 | 20.0 | 0 | 0 | 0 | 0 |
| Ir0.19Os0.22Re0.21Rh0.20Ru0.19 | HCP | 100.0 | 38.6 | 0.0 | 61.4 | 0 | 0 | 0 | 0 |
| MoNbTaTi0.25W | BCC | 94.1 | 0.0 | 94.1 | 0.0 | 0 | 0 | 0 | 0 |
| Co2Cr2Fe2Mn2Ni92 | FCC | 94.0 | 92.0 | 2.0 | 0.0 | 0 | 0 | 0 | 0 |
| MoNbTaTi0.5W | BCC | 88.9 | 0.0 | 88.9 | 0.0 | 0 | 0 | 0 | 0 |
| Al2CrCuFeNi2 | BCC | 85.7 | 71.4 | 14.3 | 0.0 | 0 | 1 | 0 | 28.57 |
| MoNbTaTi0.75W | BCC | 84.2 | 0.0 | 84.2 | 0.0 | 0 | 0 | 0 | 0 |
| AlCrMoNbTiV | BCC | 83.3 | 16.7 | 66.7 | 0.0 | 0 | 0 | 0 | 16.67 |
| MoNbTaTiVW | BCC | 83.3 | 0.0 | 83.3 | 0.0 | 0 | 0 | 0 | 0 |
| Al1.5MoNbTiV | BCC | 81.8 | 27.3 | 54.5 | 0.0 | 0 | 0 | 0 | 27.27 |
| Al0.5CrMoNbTiV | BCC | 81.8 | 9.1 | 72.7 | 0.0 | 0 | 0 | 0 | 9.09 |
| Al0.5CrCuFeNi2 | FCC | 81.8 | 63.6 | 18.2 | 0.0 | 0 | 0 | 0 | 9.09 |
| AlCrCuFeNi | BCC | 80.0 | 60.0 | 20.0 | 0.0 | 0 | 1 | 0 | 20 |
| AlCrMoNbTi | BCC | 80.0 | 20.0 | 60.0 | 0.0 | 0 | 0 | 0 | 20 |
| MoNbTaTiW | BCC | 80.0 | 0.0 | 80.0 | 0.0 | 0 | 0 | 0 | 0 |
| NbTaTiVW | BCC | 80.0 | 0.0 | 80.0 | 0.0 | 0 | 0 | 0 | 0 |
| AlMoTaTiV | BCC | 80.0 | 20.0 | 60.0 | 0.0 | 0 | 0 | 0 | 20 |
| AlCoCuNiZn | FCC | 80.0 | 60.0 | 0.0 | 20.0 | 0 | 0 | 0 | 20 |
| CrCuFeMoNi | FCC | 80.0 | 40.0 | 40.0 | 0.0 | 1 | 0 | 0 | 0 |
| CrNbTiVZn | FCC | 80.0 | 0.0 | 60.0 | 20.0 | 0 | 0 | 1 | 0 |
| CrCuFeNi2 | FCC | 80.0 | 60.0 | 20.0 | 0.0 | 0 | 0 | 0 | 0 |
| O(CoCuMgNiZn)50 | FCC | 78.4 | 39.2 | 0.0 | 39.2 | 0 | 0 | 0 | 0 |
| AlCoCrCu0.5Ni | BCC | 77.8 | 55.6 | 22.2 | 0.0 | 0 | 1 | 0 | 22.22 |
| Al0.5CrMoNbTi | BCC | 77.8 | 11.1 | 66.7 | 0.0 | 0 | 0 | 0 | 11.11 |
| AlCr0.5NbTiV | BCC | 77.8 | 22.2 | 55.6 | 0.0 | 0 | 0 | 0 | 22.22 |
| Al0.25MoNbTiV | BCC | 76.5 | 5.9 | 70.6 | 0.0 | 0 | 0 | 0 | 5.88 |
| Al0.2MoTaTiV | BCC | 76.2 | 4.8 | 71.4 | 0.0 | 0 | 0 | 0 | 4.76 |
| AlNbTiV | BCC | 75.0 | 25.0 | 50.0 | 0.0 | 0 | 0 | 0 | 25 |
| CrFeMoV | BCC | 75.0 | 0.0 | 75.0 | 0.0 | 0 | 0 | 0 | 0 |
| MoTaTiV | BCC | 75.0 | 0.0 | 75.0 | 0.0 | 0 | 0 | 0 | 0 |
| NbTaTiV | BCC | 75.0 | 0.0 | 75.0 | 0.0 | 0 | 0 | 0 | 0 |
| CrMoNbTaTiVWZr | BCC | 75.0 | 0.0 | 75.0 | 0.0 | 0 | 0 | 0 | 0 |
| AlCuTiNi | FCC | 75.0 | 75.0 | 0.0 | 0.0 | 0 | 0 | 0 | 25 |
| CoCrCuNi | FCC | 75.0 | 50.0 | 25.0 | 0.0 | 0 | 0 | 0 | 0 |
| CoCuNiZn | FCC | 75.0 | 50.0 | 0.0 | 25.0 | 0 | 0 | 0 | 0 |
| Ni50(AlCoCrFe)50 | FCC | 75.0 | 62.5 | 12.5 | 0.0 | 0 | 0 | 0 | 12.5 |
| CrCuFeNi | FCC | 75.0 | 50.0 | 25.0 | 0.0 | 0 | 0 | 0 | 0 |
| AlCrTiV | B2 | 75.0 | 25.0 | 50.0 | 0.0 | 0 | 0 | 0 | 25 |
| Al3CoCrCuFeNi | B2 | 75.0 | 62.5 | 12.5 | 0.0 | 0 | 0 | 0 | 37.5 |
| Al0.85CuFeNi | BCC | 74.0 | 74.0 | 0.0 | 0.0 | 0 | 1 | 0 | 22.08 |
| Al0.6MoTaTi | BCC | 72.2 | 16.7 | 55.6 | 0.0 | 0 | 0 | 0 | 16.67 |
| AlCoCrCuFeNiW | BCC | 71.4 | 42.9 | 28.6 | 0.0 | 0 | 1 | 0 | 14.29 |
| CrMoNbTaTiVZr | BCC | 71.4 | 0.0 | 71.4 | 0.0 | 0 | 0 | 0 | 0 |
| Al2CoCrCuFeNi | BCC | 71.4 | 57.1 | 14.3 | 0.0 | 0 | 1 | 0 | 28.57 |
| Al0.5CuFeNi | FCC | 71.4 | 71.4 | 0.0 | 0.0 | 0 | 0 | 0 | 14.29 |
| Al3CoCrFeNi | B2 | 71.4 | 57.1 | 14.3 | 0.0 | 0 | 0 | 0 | 42.86 |
| Al1.67CoCrCuFeNi | BCC | 70.0 | 55.0 | 15.0 | 0.0 | 0 | 1 | 0 | 25.04 |
| Al0.3CuFeNi | FCC | 69.7 | 69.7 | 0.0 | 0.0 | 0 | 0 | 0 | 9.09 |
| Al0.5CoCrCuFeNiV | BCC | 69.2 | 38.5 | 30.8 | 0.0 | 0 | 1 | 0 | 7.69 |
| Al2.3B0.15CoCrCu0.7FeNiSi0.1 | BCC | 69.0 | 55.2 | 13.8 | 0.0 | 0 | 1 | 0 | 31.72 |
| Al1.25CoCrCuFeNi | BCC | 68.0 | 52.0 | 16.0 | 0.0 | 0 | 1 | 0 | 20 |
| AlCoCrCuFeNiV0.2 | FCC | 67.7 | 48.4 | 19.4 | 0.0 | 0 | 0 | 0 | 16.13 |
| Al2.3B0.3CoCrCu0.7FeNiSi0.1 | BCC | 67.6 | 54.1 | 13.5 | 0.0 | 0 | 1 | 0 | 31.08 |
| Al0.7Co0.3CrFeNi | BCC | 67.5 | 42.5 | 25.0 | 0.0 | 0 | 1 | 0 | 17.5 |
| Hf8Nb33Ta34 Ti11Zr14 | BCC | 67.0 | 0.0 | 67.0 | 0.0 | 0 | 0 | 0 | 0 |
| Cr33.33(CoCuFeNi)66.7 | FCC | 66.8 | 33.2 | 33.6 | 0.0 | 0 | 0 | 1 | 0 |
| CrTiV | BCC | 66.7 | 0.0 | 66.7 | 0.0 | 0 | 0 | 0 | 0 |
| AlCoCrCuFeNi | BCC | 66.7 | 50.0 | 16.7 | 0.0 | 0 | 1 | 0 | 16.67 |
| AlCoCuNiTiZn | BCC | 66.7 | 50.0 | 0.0 | 16.7 | 0 | 1 | 0 | 16.67 |
| AlCrCuFeTiZn | BCC | 66.7 | 33.3 | 16.7 | 16.7 | 0 | 1 | 0 | 16.67 |
| MoNbTaTiVZr | BCC | 66.7 | 0.0 | 66.7 | 0.0 | 0 | 0 | 0 | 0 |
| Al2CoCrFeNi | BCC | 66.7 | 50.0 | 16.7 | 0.0 | 0 | 1 | 0 | 33.33 |
| AlCoCrFeNiV | BCC | 66.7 | 33.3 | 33.3 | 0.0 | 1 | 0 | 0 | 16.67 |
| AlMo0.5NbTa0.5TiZr0.5 | BCC | 66.7 | 22.2 | 44.4 | 0.0 | 0 | 0 | 0 | 22.22 |
| CoCrNi | FCC | 66.7 | 33.3 | 33.3 | 0.0 | 1 | 0 | 0 | 0 |
| CoCuNi | FCC | 66.7 | 66.7 | 0.0 | 0.0 | 0 | 0 | 0 | 0 |
| AlCoCuFeNiV | FCC | 66.7 | 50.0 | 16.7 | 0.0 | 0 | 0 | 0 | 16.67 |
| Al0.4CoCu0.6Ni | FCC | 66.7 | 66.7 | 0.0 | 0.0 | 0 | 0 | 0 | 13.33 |
| Al0.5CoCrCu0.5FeNi2 | FCC | 66.7 | 50.0 | 16.7 | 0.0 | 0 | 0 | 0 | 8.33 |
| AlCoNi | B2 | 66.7 | 66.7 | 0.0 | 0.0 | 0 | 0 | 0 | 33.33 |
| Al0.5CoCrCuFeNiV0.4 | FCC | 66.1 | 42.4 | 23.7 | 0.0 | 0 | 0 | 0 | 8.47 |
| Al0.8824CoCrCuFeNi | FCC | 66.0 | 49.0 | 17.0 | 0.0 | 0 | 0 | 0 | 15 |
| Ag1.2(BiSbTe1.5Se1.5)98.8 | RHOM | 65.9 | 54.5 | 0.0 | 0.0 | 0 | 0 | 0 | 0 |
| Al0.4CoCu0.6NiSi0.05 | FCC | 65.6 | 65.6 | 0.0 | 0.0 | 0 | 0 | 0 | 13.11 |
| Al2.3B0.6CoCrCu0.7FeNiSi0.1 | BCC | 64.9 | 51.9 | 13.0 | 0.0 | 0 | 1 | 0 | 29.87 |
| AlCoCrCu0.5FeNi | BCC | 63.6 | 45.5 | 18.2 | 0.0 | 0 | 1 | 0 | 18.18 |
| CoCrCu1.5FeNi | FCC | 63.6 | 45.5 | 18.2 | 0.0 | 0 | 0 | 0 | 0 |
| Al0.5CoCrCuFeNi | FCC | 63.6 | 45.5 | 18.2 | 0.0 | 0 | 0 | 0 | 9.09 |
| Al1.5CoCrFeNi | B2 | 63.6 | 45.5 | 18.2 | 0.0 | 0 | 0 | 0 | 27.27 |
| Al0.4945CoCrCuFeNi | FCC | 63.6 | 45.4 | 18.2 | 0.0 | 0 | 0 | 0 | 9 |
| AlCoCrCuFeNiWZr | BCC | 62.5 | 37.5 | 25.0 | 0.0 | 0 | 1 | 0 | 12.5 |
| AlNbTa0.5TiZr0.5 | BCC | 62.5 | 25.0 | 37.5 | 0.0 | 0 | 0 | 0 | 25 |
| CoCrFe0.2Ni | FCC | 62.5 | 31.3 | 31.3 | 0.0 | 1 | 0 | 0 | 0 |
| CrCu2Fe2MnNi2 | FCC | 62.5 | 50.0 | 12.5 | 0.0 | 0 | 0 | 0 | 0 |
| CoCrFeMnNi3V | FCC | 62.5 | 37.5 | 25.0 | 0.0 | 0 | 0 | 0 | 0 |
| Mo1.3NbTiVZr | BCC | 62.3 | 0.0 | 62.3 | 0.0 | 0 | 0 | 0 | 0 |
| AlCoCrCu0.25FeNi | BCC | 61.9 | 42.9 | 19.0 | 0.0 | 0 | 1 | 0 | 19.05 |
| CoCrCuFeIn0.2466Ni | FCC | 61.9 | 38.1 | 19.1 | 0.0 | 0 | 0 | 0 | 0 |
| Co19Cr19.2Cu23.5Fe19.2Ni19.1 | FCC | 61.8 | 42.6 | 19.2 | 0.0 | 0 | 0 | 0 | 0 |
| Nb4(CoCrCuFeNi)96 | FCC | 61.6 | 38.4 | 23.2 | 0.0 | 0 | 0 | 0 | 0 |
| AlCoCrFeMo0.1Ni | BCC | 60.8 | 39.2 | 21.6 | 0.0 | 0 | 1 | 0 | 19.61 |
| AlCoCrFeNb0.1Ni | BCC | 60.8 | 39.2 | 21.6 | 0.0 | 0 | 1 | 0 | 19.61 |
| Sc0.03(Al2CoCrFeNi)0.97 | BCC | 60.6 | 39.4 | 19.7 | 1.5 | 0 | 1 | 0 | 19.7 |
| AlCoCrCuFe | BCC | 60.0 | 40.0 | 20.0 | 0.0 | 0 | 1 | 0 | 20 |
| AlCoCrFeNi | BCC | 60.0 | 40.0 | 20.0 | 0.0 | 0 | 1 | 0 | 20 |
| AlCoCrNiSi | BCC | 60.0 | 40.0 | 20.0 | 0.0 | 0 | 1 | 0 | 20 |
| AlCrFeTiZn | BCC | 60.0 | 20.0 | 20.0 | 20.0 | 1 | 0 | 0 | 20 |
| AlCuFeNiTi | BCC | 60.0 | 60.0 | 0.0 | 0.0 | 0 | 1 | 0 | 20 |
| MoTaTiVZr | BCC | 60.0 | 0.0 | 60.0 | 0.0 | 0 | 0 | 0 | 0 |
| NbTaTiVZr | BCC | 60.0 | 0.0 | 60.0 | 0.0 | 0 | 0 | 0 | 0 |
| Al18Co20Cr21Fe20Ni21 | BCC | 60.0 | 39.0 | 21.0 | 0.0 | 0 | 1 | 0 | 18 |
| Al0.5CrNbTi2V0.5 | BCC | 60.0 | 10.0 | 50.0 | 0.0 | 0 | 0 | 0 | 10 |
| AlNb1.5Ta0.5Ti1.5Zr0.5 | BCC | 60.0 | 20.0 | 40.0 | 0.0 | 0 | 0 | 0 | 20 |
| AlMo0.5NbTa0.5TiZr | BCC | 60.0 | 20.0 | 40.0 | 0.0 | 0 | 0 | 0 | 20 |
| Al2CoCrCuFeMnNiTiV | BCC | 60.0 | 40.0 | 20.0 | 0.0 | 0 | 1 | 0 | 20 |
| CoCrCuFeNi | FCC | 60.0 | 40.0 | 20.0 | 0.0 | 0 | 0 | 0 | 0 |
| CoCuFeNiV | FCC | 60.0 | 40.0 | 20.0 | 0.0 | 0 | 0 | 0 | 0 |
| CuFeMnNiPt | FCC | 60.0 | 60.0 | 0.0 | 0.0 | 0 | 0 | 0 | 0 |
| Ni40(CoCrFe)60 | FCC | 60.0 | 40.0 | 20.0 | 0.0 | 0 | 0 | 0 | 0 |
| Al0.3CoCrFeNi1.7 | FCC | 60.0 | 40.0 | 20.0 | 0.0 | 0 | 0 | 0 | 6 |
| Al13Co20Cr23.5Fe20Ni23.5 | FCC | 60.0 | 36.5 | 23.5 | 0.0 | 0 | 0 | 0 | 13 |
| Co4(AlCoCrFeNi)96 | FCC | 60.0 | 40.0 | 20.0 | 0.0 | 0 | 0 | 0 | 20 |
| Co15Cu25Fe15Mn10Ni35 | FCC | 60.0 | 60.0 | 0.0 | 0.0 | 0 | 0 | 0 | 0 |
| Al0.5CoCrCu0.5FeNi | FCC | 60.0 | 40.0 | 20.0 | 0.0 | 0 | 0 | 0 | 10 |
| Al0.5CoCrFeMo0.5Ni | FCC | 60.0 | 30.0 | 30.0 | 0.0 | 1 | 0 | 0 | 10 |
| Al0.9CoCrFeNi | BCC | 59.2 | 38.8 | 20.4 | 0.0 | 0 | 1 | 0 | 18.37 |
| CoCrFe0.4Ni | FCC | 58.8 | 29.4 | 29.4 | 0.0 | 1 | 0 | 0 | 0 |
| Al0.85CoCrFeNi | BCC | 58.8 | 38.1 | 20.6 | 0.0 | 0 | 1 | 0 | 17.53 |
| Mo1.5NbTiV0.3Zr | BCC | 58.3 | 0.0 | 58.3 | 0.0 | 0 | 0 | 0 | 0 |
| Al1.5CoCrFeNiTi0.5 | BCC | 58.3 | 41.7 | 16.7 | 0.0 | 0 | 1 | 0 | 25 |
| Al0.3CoCrCu0.5FeNi | FCC | 58.3 | 37.5 | 20.8 | 0.0 | 0 | 0 | 0 | 6.25 |
| Al0.6CoCrCu0.4FeNiSi0.2 | BCC | 57.7 | 38.5 | 19.2 | 0.0 | 0 | 1 | 0 | 11.54 |
| Al0.8CoCrCu0.2FeNiSi0.2 | BCC | 57.7 | 38.5 | 19.2 | 0.0 | 0 | 1 | 0 | 15.38 |
| Al0.9CoCrCu0.1FeNiSi0.2 | BCC | 57.7 | 38.5 | 19.2 | 0.0 | 0 | 1 | 0 | 17.31 |
| Al0.2CoCrCu0.8FeNiSi0.2 | FCC | 57.7 | 38.5 | 19.2 | 0.0 | 0 | 0 | 0 | 3.85 |
| Al0.4CoCrCu0.6FeNiSi0.2 | FCC | 57.7 | 38.5 | 19.2 | 0.0 | 0 | 0 | 0 | 7.69 |
| Ni42.9(CoCrFeMn)57.1 | FCC | 57.2 | 42.9 | 14.3 | 0.0 | 0 | 0 | 0 | 0 |
| CrCuFeMn2Ni2 | FCC | 57.1 | 42.9 | 14.3 | 0.0 | 0 | 0 | 0 | 0 |
| CoCrFeMnNi2V | FCC | 57.1 | 28.6 | 28.6 | 0.0 | 1 | 0 | 0 | 0 |
| Al0.3B0.15CoCrFeNiCu0.7Si0.1 | FCC | 57.1 | 38.1 | 19.0 | 0.0 | 0 | 0 | 0 | 5.71 |
| Al0.65CoCrFeNi | FCC | 57.0 | 35.5 | 21.5 | 0.0 | 0 | 0 | 0 | 13.98 |
| Al0.5CoCrFeMo0.1Ni | FCC | 56.5 | 32.6 | 23.9 | 0.0 | 0 | 0 | 0 | 10.87 |
| MoNbTiV0.5Zr | BCC | 55.6 | 0.0 | 55.6 | 0.0 | 0 | 0 | 0 | 0 |
| Al0.5Mo0.5NbTa0.5TiZr | BCC | 55.6 | 11.1 | 44.4 | 0.0 | 0 | 0 | 0 | 11.11 |
| CoCrFe0.6Ni | FCC | 55.6 | 27.8 | 27.8 | 0.0 | 1 | 0 | 0 | 0 |
| CoFeNi2W0.5 | FCC | 55.6 | 44.4 | 11.1 | 0.0 | 0 | 0 | 0 | 0 |
| Al0.5CoCrFeNi | FCC | 55.6 | 33.3 | 22.2 | 0.0 | 0 | 0 | 0 | 11.11 |
| CoCrCu0.5FeNi | FCC | 55.6 | 33.3 | 22.2 | 0.0 | 0 | 0 | 0 | 0 |
| Cr2CuFe2Mn2Ni2 | FCC | 55.6 | 33.3 | 22.2 | 0.0 | 0 | 0 | 0 | 0 |
| CoCrFeMo0.5Ni | FCC | 55.6 | 22.2 | 33.3 | 0.0 | 0 | 0 | 1 | 0 |
| Al0.3B0.3CoCrFeNiCu0.7Si0.1 | FCC | 55.6 | 37.0 | 18.5 | 0.0 | 0 | 0 | 0 | 5.56 |
| Al0.45CoCrFeNi | FCC | 55.1 | 32.6 | 22.5 | 0.0 | 0 | 0 | 0 | 10.11 |
| Co20Cr20Fe20Mn5Ni20Zn15 | FCC | 55.0 | 20.0 | 20.0 | 15.0 | 1 | 0 | 0 | 0 |
| Al0.75CoCrCu0.25FeNiTi0.5 | BCC | 54.6 | 36.4 | 18.2 | 0.0 | 0 | 1 | 0 | 13.64 |
| Al0.4CoCrFeNi | FCC | 54.6 | 31.8 | 22.7 | 0.0 | 0 | 0 | 0 | 9.09 |
| Al0.3CoCrFeMo0.1Ni | FCC | 54.6 | 29.5 | 25.0 | 0.0 | 0 | 0 | 0 | 6.82 |
| CoCrCuFeNiTi0.5 | FCC | 54.6 | 36.4 | 18.2 | 0.0 | 0 | 0 | 0 | 0 |
| Al0.25CoCrCu0.75FeNiTi0.5 | FCC | 54.6 | 36.4 | 18.2 | 0.0 | 0 | 0 | 0 | 4.55 |
| Al0.375CoCrFeNi | FCC | 54.3 | 31.4 | 22.9 | 0.0 | 0 | 0 | 0 | 8.57 |
| AlCrFeMo0.5NiSiTi | BCC | 53.9 | 30.8 | 23.1 | 0.0 | 0 | 1 | 0 | 15.38 |
| Mo0.3NbTiVZr | BCC | 53.5 | 0.0 | 53.5 | 0.0 | 0 | 0 | 0 | 0 |
| Al0.3CoCrFeNi | FCC | 53.5 | 30.2 | 23.3 | 0.0 | 0 | 0 | 0 | 6.98 |
| CoCrFeMo0.3Ni | FCC | 53.5 | 23.3 | 30.2 | 0.0 | 0 | 0 | 1 | 0 |
| Al6.64Co23.82Cr23.66Fe23.01Ni22.87 | FCC | 53.2 | 29.5 | 23.7 | 0.0 | 0 | 0 | 0 | 6.64 |
| Al0.25NbTaTiZr | BCC | 52.9 | 5.9 | 47.1 | 0.0 | 0 | 0 | 0 | 5.88 |
| Al0.25CoCrFeNi | FCC | 52.9 | 29.4 | 23.5 | 0.0 | 0 | 0 | 0 | 5.88 |
| Al0.3B0.6CoCrFeNiCu0.7Si0.1 | FCC | 52.6 | 35.1 | 17.5 | 0.0 | 0 | 0 | 0 | 5.26 |
| Mo5(NbTaTiZr)95 | BCC | 52.5 | 0.0 | 52.5 | 0.0 | 0 | 0 | 0 | 0 |
| Al0.2CoCrFeNi | FCC | 52.4 | 28.6 | 23.8 | 0.0 | 0 | 0 | 0 | 4.76 |
| Al0.3CoCrFeMn0.1Ni | FCC | 52.3 | 29.5 | 22.7 | 0.0 | 0 | 0 | 0 | 6.82 |
| Al0.3CoCrFeNiTi0.1 | FCC | 52.3 | 29.5 | 22.7 | 0.0 | 0 | 0 | 0 | 6.82 |
| Al1.2CrFe1.5MnNi0.5 | BCC | 51.9 | 32.7 | 19.2 | 0.0 | 0 | 1 | 0 | 23.08 |
| Co24.1Cr24.1Fe24.1Mo3.6Ni24.1 | FCC | 51.8 | 24.1 | 27.7 | 0.0 | 0 | 0 | 1 | 0 |
| AlCoCrFeNiSi0.8 | BCC | 51.7 | 34.5 | 17.2 | 0.0 | 0 | 1 | 0 | 17.24 |
| Al0.1CoCrFeNi | FCC | 51.2 | 26.8 | 24.4 | 0.0 | 0 | 0 | 0 | 2.44 |
| CoCrFeNiTa0.1 | FCC | 51.2 | 24.4 | 26.8 | 0.0 | 0 | 0 | 1 | 0 |
| Al4.88Co29.53Cr18.58Fe19.62Ni27.39 | FCC | 50.9 | 32.3 | 18.6 | 0.0 | 0 | 0 | 0 | 4.88 |
| CoCrCuFeGe0.9666Ni | FCC | 50.3 | 33.5 | 16.8 | 0.0 | 0 | 0 | 0 | 0 |
| Co25.33Cr25.77Fe24.53Ni24.37 | FCC | 50.1 | 24.4 | 25.8 | 0.0 | 0 | 0 | 1 | 0 |
| AlCoFeNi | BCC | 50.0 | 50.0 | 0.0 | 0.0 | 0 | 1 | 0 | 25 |
| AlCrFeTi | BCC | 50.0 | 25.0 | 25.0 | 0.0 | 1 | 0 | 0 | 25 |
| MoTiVZr | BCC | 50.0 | 0.0 | 50.0 | 0.0 | 0 | 0 | 0 | 0 |
| NbTaTiZr | BCC | 50.0 | 0.0 | 50.0 | 0.0 | 0 | 0 | 0 | 0 |
| NbTiVZr | BCC | 50.0 | 0.0 | 50.0 | 0.0 | 0 | 0 | 0 | 0 |
| AlCoCrFeNiTi | BCC | 50.0 | 33.3 | 16.7 | 0.0 | 0 | 1 | 0 | 16.67 |
| CoCrFeMnNiW | BCC | 50.0 | 16.7 | 33.3 | 0.0 | 0 | 0 | 0 | 0 |
| HfMoNbTaTiZr | BCC | 50.0 | 0.0 | 50.0 | 0.0 | 0 | 0 | 0 | 0 |
| HfNbTaTiVZr | BCC | 50.0 | 0.0 | 50.0 | 0.0 | 0 | 0 | 0 | 0 |
| Ag2Cu2DyGdTbY | BCC | 50.0 | 50.0 | 0.0 | 0.0 | 0 | 1 | 0 | 0 |
| HfMo1NbTaTiZr | BCC | 50.0 | 0.0 | 50.0 | 0.0 | 0 | 0 | 0 | 0 |
| CoCr5Fe5MoNbSiTiW | BCC | 50.0 | 0.0 | 50.0 | 0.0 | 0 | 0 | 0 | 0 |
| CoCrFeNi | FCC | 50.0 | 25.0 | 25.0 | 0.0 | 1 | 0 | 0 | 0 |
| CoCrMnNi | FCC | 50.0 | 25.0 | 25.0 | 0.0 | 1 | 0 | 0 | 0 |
| CoCuFeNi | FCC | 50.0 | 50.0 | 0.0 | 0.0 | 0 | 0 | 0 | 0 |
| CoFeNiV | FCC | 50.0 | 25.0 | 25.0 | 0.0 | 1 | 0 | 0 | 0 |
| CoCrFeMnNiCu | FCC | 50.0 | 33.3 | 16.7 | 0.0 | 0 | 0 | 0 | 0 |
| CoCrFeMnNiNb | FCC | 50.0 | 16.7 | 33.3 | 0.0 | 0 | 0 | 1 | 0 |
| CoCrFeMnNiV | FCC | 50.0 | 16.7 | 33.3 | 0.0 | 0 | 0 | 1 | 0 |
| Al7.5Co25 Cu17.5Fe25 Ni25 | FCC | 50.0 | 50.0 | 0.0 | 0.0 | 0 | 0 | 0 | 7.5 |
| CrFeMnNi | FCC | 50.0 | 25.0 | 25.0 | 0.0 | 1 | 0 | 0 | 0 |
| Al0.3CoCrFeMn0.3Ni | FCC | 50.0 | 28.3 | 21.7 | 0.0 | 0 | 0 | 0 | 6.52 |
| Co10Cr15Fe35Mn5Ni25V10 | FCC | 50.0 | 25.0 | 25.0 | 0.0 | 1 | 0 | 0 | 0 |
| CoFeReRu | HCP | 50.0 | 0.0 | 0.0 | 50.0 | 1 | 0 | 0 | 0 |
| Co33.33(CrCuFeNi)66.7 | FCC | 49.8 | 33.2 | 16.6 | 0.0 | 0 | 0 | 0 | 0 |
| Al16(CoCrFeMnNi)84 | B2 | 49.6 | 32.8 | 16.8 | 0.0 | 0 | 0 | 0 | 16 |
| C0.05CoCrFeNi | FCC | 49.4 | 24.7 | 24.7 | 0.0 | 1 | 0 | 0 | 0 |
| CoCuFeNiSn0.05 | FCC | 49.4 | 49.4 | 0.0 | 0.0 | 0 | 0 | 0 | 0 |
| Al10Co17Fe34Mo5Ni34 | FCC | 49.0 | 44.0 | 5.0 | 0.0 | 0 | 0 | 0 | 10 |
| Al0.4Hf0.6NbTaTiZr | BCC | 48.0 | 8.0 | 40.0 | 0.0 | 0 | 0 | 0 | 8 |
| Al0.8CrFe1.5MnNi0.5 | BCC | 47.9 | 27.1 | 20.8 | 0.0 | 0 | 1 | 0 | 16.67 |
| Al0.3CrFe1.5MnNi | BCC | 47.9 | 27.1 | 20.8 | 0.0 | 0 | 1 | 0 | 6.25 |
| Co5(CrFeMnNi)95 | FCC | 47.5 | 23.8 | 23.8 | 0.0 | 1 | 0 | 0 | 0 |
| Al0.2CoCrFeNiTi0.5 | FCC | 46.8 | 25.5 | 21.3 | 0.0 | 0 | 0 | 0 | 4.26 |
| AlCoCrFeMo0.5NiSiTi | BCC | 46.7 | 26.7 | 20.0 | 0.0 | 0 | 1 | 0 | 13.33 |
| Ni20(CoCrFe)80 | FCC | 46.7 | 20.0 | 26.7 | 0.0 | 0 | 0 | 1 | 0 |
| CoCrFeNiTi0.3 | FCC | 46.5 | 23.3 | 23.3 | 0.0 | 1 | 0 | 0 | 0 |
| Co1.5CrFeMo0.1Ni1.5Ti0.5 | FCC | 46.4 | 26.8 | 19.6 | 0.0 | 0 | 0 | 0 | 0 |
| Al0.3NbTa0.8Ti1.4V0.2Zr1.3 | BCC | 46.0 | 6.0 | 40.0 | 0.0 | 0 | 0 | 0 | 6 |
| HfMoNb1.5TiZr | BCC | 45.5 | 0.0 | 45.5 | 0.0 | 0 | 0 | 0 | 0 |
| HfMo1.5NbTiZr | BCC | 45.5 | 0.0 | 45.5 | 0.0 | 0 | 0 | 0 | 0 |
| Co1.5CrFeNi1.5Ti0.5 | FCC | 45.5 | 27.3 | 18.2 | 0.0 | 0 | 0 | 0 | 0 |
| Al0.5CoCrFeMnNi | FCC | 45.5 | 27.3 | 18.2 | 0.0 | 0 | 0 | 0 | 9.09 |
| C9.302(CoCrFeNi)90.698 | FCC | 45.3 | 22.7 | 22.7 | 0.0 | 1 | 0 | 0 | 0 |
| Co15Cr20Fe20Mn20Ni25 | FCC | 45.0 | 25.0 | 20.0 | 0.0 | 0 | 0 | 0 | 0 |
| Co10(CrFeMnNi)90 | FCC | 45.0 | 22.5 | 22.5 | 0.0 | 1 | 0 | 0 | 0 |
| Co35Cr15Fe20 Mo10Ni20 | FCC | 45.0 | 20.0 | 25.0 | 0.0 | 0 | 0 | 1 | 0 |
| Al8(CoCrFeMnNi)92 | FCC | 44.8 | 26.4 | 18.4 | 0.0 | 0 | 0 | 0 | 8 |
| Al0.5CrFe1.5MnNi0.5 | BCC | 44.4 | 22.2 | 22.2 | 0.0 | 1 | 0 | 0 | 11.11 |
| HfMoNbTi0.5Zr | BCC | 44.4 | 0.0 | 44.4 | 0.0 | 0 | 0 | 0 | 0 |
| HfMoNbTiZr0.5 | BCC | 44.4 | 0.0 | 44.4 | 0.0 | 0 | 0 | 0 | 0 |
| Hf0.5MoNbTiZr | BCC | 44.4 | 0.0 | 44.4 | 0.0 | 0 | 0 | 0 | 0 |
| C11.01(CoCrFeNi)88.99 | FCC | 44.4 | 22.2 | 22.2 | 0.0 | 1 | 0 | 0 | 0 |
| CoCrFeMn0.5Ni | FCC | 44.4 | 22.2 | 22.2 | 0.0 | 1 | 0 | 0 | 0 |
| CoCrFeNiTi0.5 | FCC | 44.4 | 22.2 | 22.2 | 0.0 | 1 | 0 | 0 | 0 |
| Mn14(CoCrFeNi)86 | FCC | 43.0 | 21.5 | 21.5 | 0.0 | 1 | 0 | 0 | 0 |
| HfNb2.0TiVZr2.0 | BCC | 42.9 | 0.0 | 42.9 | 0.0 | 0 | 0 | 0 | 0 |
| CoCrCu0.25FeMnNi | FCC | 42.9 | 23.8 | 19.0 | 0.0 | 0 | 0 | 0 | 0 |
| CoCrFeMnNiV0.25 | FCC | 42.9 | 19.0 | 23.8 | 0.0 | 0 | 0 | 1 | 0 |
| AlCoFeNiTi | BCC | 40.0 | 40.0 | 0.0 | 0.0 | 0 | 1 | 0 | 20 |
| CuNiSiTiZr | BCC | 40.0 | 40.0 | 0.0 | 0.0 | 0 | 1 | 0 | 0 |
| HfMoNbTiZr | BCC | 40.0 | 0.0 | 40.0 | 0.0 | 0 | 0 | 0 | 0 |
| HfMoTaTiZr | BCC | 40.0 | 0.0 | 40.0 | 0.0 | 0 | 0 | 0 | 0 |
| HfNbTaTiZr | BCC | 40.0 | 0.0 | 40.0 | 0.0 | 0 | 0 | 0 | 0 |
| HfNbTiVZr | BCC | 40.0 | 0.0 | 40.0 | 0.0 | 0 | 0 | 0 | 0 |
| Al20Co20Cr20(FeMn)40 | BCC | 40.0 | 20.0 | 20.0 | 0.0 | 1 | 0 | 0 | 20 |
| Al0.3CrFe1.5MnNi0.5Ti0.2 | BCC | 40.0 | 17.8 | 22.2 | 0.0 | 0 | 0 | 0 | 6.67 |
| CoCrFeMn0.5NiTi0.5 | BCC | 40.0 | 20.0 | 20.0 | 0.0 | 1 | 0 | 0 | 0 |
| CoCrFeMnNi | FCC | 40.0 | 20.0 | 20.0 | 0.0 | 1 | 0 | 0 | 0 |
| CoCrFeNiTi | FCC | 40.0 | 20.0 | 20.0 | 0.0 | 1 | 0 | 0 | 0 |
| CoCuFeMnNi | FCC | 40.0 | 40.0 | 0.0 | 0.0 | 0 | 0 | 0 | 0 |
| CoCuFeNiTi | FCC | 40.0 | 40.0 | 0.0 | 0.0 | 0 | 0 | 0 | 0 |
| CrTiVYZr | FCC | 40.0 | 0.0 | 40.0 | 0.0 | 0 | 0 | 1 | 0 |
| Ni40(CoFeMn)60 | FCC | 40.0 | 40.0 | 0.0 | 0.0 | 0 | 0 | 0 | 0 |
| Co20(CrFeMnNi)80 | FCC | 40.0 | 20.0 | 20.0 | 0.0 | 1 | 0 | 0 | 0 |
| Co5Cu15Fe30Mn25Ni25 | FCC | 40.0 | 40.0 | 0.0 | 0.0 | 0 | 0 | 0 | 0 |
| CoCuFe0.25Mn1.75Ni | FCC | 40.0 | 40.0 | 0.0 | 0.0 | 0 | 0 | 0 | 0 |
| Dy20Er20Gd20Ho20Tb20 | HCP | 40.0 | 0.0 | 0.0 | 40.0 | 1 | 0 | 0 | 0 |
| DyGdLuTbTm | HCP | 40.0 | 0.0 | 0.0 | 40.0 | 1 | 0 | 0 | 0 |
| CoCrFeMnV | SIGMA | 40.0 | 0.0 | 40.0 | 0.0 | 0 | 0 | 0 | 0 |
| C0.01CoCrFeMnNi | FCC | 39.9 | 20.0 | 20.0 | 0.0 | 1 | 0 | 0 | 0 |
| NbTiV0.3Zr | BCC | 39.4 | 0.0 | 39.4 | 0.0 | 0 | 0 | 0 | 0 |
| CoCrFeMnNiTi0.1 | FCC | 39.2 | 19.6 | 19.6 | 0.0 | 1 | 0 | 0 | 0 |
| CoCu0.9 Fe1.05Mn1.05Ni | FCC | 38.0 | 38.0 | 0.0 | 0.0 | 0 | 0 | 0 | 0 |
| Al0.5CoFeNiSi0.5 | BCC | 37.5 | 37.5 | 0.0 | 0.0 | 0 | 1 | 0 | 12.5 |
| Hf0.5Mo0.5NbTiZr | BCC | 37.5 | 0.0 | 37.5 | 0.0 | 0 | 0 | 0 | 0 |
| Co42.5Cr12.5Fe20Mo5Ni20 | FCC | 37.5 | 20.0 | 17.5 | 0.0 | 0 | 0 | 0 | 0 |
| HfMoNbTi1.5Zr | BCC | 36.4 | 0.0 | 36.4 | 0.0 | 0 | 0 | 0 | 0 |
| HfMoNbTiZr1.5 | BCC | 36.4 | 0.0 | 36.4 | 0.0 | 0 | 0 | 0 | 0 |
| Hf1.5MoNbTiZr | BCC | 36.4 | 0.0 | 36.4 | 0.0 | 0 | 0 | 0 | 0 |
| Ni15(CoCrFeMn)85 | FCC | 36.3 | 15.0 | 21.3 | 0.0 | 0 | 0 | 1 | 0 |
| Al0.3CoFeNiSi0.3 | FCC | 36.1 | 36.1 | 0.0 | 0.0 | 0 | 0 | 0 | 8.33 |
| Al0.2CoFeNiSi0.2 | FCC | 35.3 | 35.3 | 0.0 | 0.0 | 0 | 0 | 0 | 5.88 |
| AlFeTi | BCC | 33.3 | 33.3 | 0.0 | 0.0 | 0 | 1 | 0 | 33.33 |
| HfNbZr | BCC | 33.3 | 0.0 | 33.3 | 0.0 | 0 | 0 | 0 | 0 |
| CoCuHfPdTiZr | BCC | 33.3 | 33.3 | 0.0 | 0.0 | 0 | 1 | 0 | 0 |
| HfMoNb0.5TiZr | BCC | 33.3 | 0.0 | 33.3 | 0.0 | 0 | 0 | 0 | 0 |
| Co0.5Fe0.5MgNi0.5TiZr | BCC | 33.3 | 11.1 | 0.0 | 22.2 | 0 | 1 | 0 | 0 |
| CoFeNi | FCC | 33.3 | 33.3 | 0.0 | 0.0 | 0 | 0 | 0 | 0 |
| CoMnNi | FCC | 33.3 | 33.3 | 0.0 | 0.0 | 0 | 0 | 0 | 0 |
| FeMnNi | FCC | 33.3 | 33.3 | 0.0 | 0.0 | 0 | 0 | 0 | 0 |
| CoFeNi(AlCu)0.2 | FCC | 33.3 | 33.3 | 0.0 | 0.0 | 0 | 0 | 0 | 0 |
| CoFeNi(AlCu)0.4 | FCC | 33.3 | 33.3 | 0.0 | 0.0 | 0 | 0 | 0 | 0 |
| CoFeNi(AlCu)0.6 | FCC | 33.3 | 33.3 | 0.0 | 0.0 | 0 | 0 | 0 | 0 |
| CoFeNi(AlCu)0.7 | FCC | 33.3 | 33.3 | 0.0 | 0.0 | 0 | 0 | 0 | 0 |
| CoFeNi(AlCu)0.8 | FCC | 33.3 | 33.3 | 0.0 | 0.0 | 0 | 0 | 0 | 0 |
| Co33.33(CrFeMnNi)66.7 | FCC | 33.2 | 16.6 | 16.6 | 0.0 | 1 | 0 | 0 | 0 |
| Al0.3CoFeNiSi | BCC | 30.2 | 30.2 | 0.0 | 0.0 | 0 | 1 | 0 | 6.98 |
| Hf15Nb20Ta10Ti30Zr25 | BCC | 30.0 | 0.0 | 30.0 | 0.0 | 0 | 0 | 0 | 0 |
| Co30Fe30Mn10Ni30 | FCC | 30.0 | 30.0 | 0.0 | 0.0 | 0 | 0 | 0 | 0 |
| Co30Fe30Ni30Ti10 | FCC | 30.0 | 30.0 | 0.0 | 0.0 | 0 | 0 | 0 | 0 |
| Co26Fe27Mn10Ni27Ti10 | FCC | 27.0 | 27.0 | 0.0 | 0.0 | 0 | 0 | 0 | 0 |
| CoFeMnTi2.5V3Zr3 | Laves C14 | 26.1 | 0.0 | 26.1 | 0.0 | 0 | 0 | 0 | 0 |
| HfNbTiZr | BCC | 25.0 | 0.0 | 25.0 | 0.0 | 0 | 0 | 0 | 0 |
| HfMo0.5Nb0.5TiZr | BCC | 25.0 | 0.0 | 25.0 | 0.0 | 0 | 0 | 0 | 0 |
| HfNb0.5Ta0.5TiZr | BCC | 25.0 | 0.0 | 25.0 | 0.0 | 0 | 0 | 0 | 0 |
| HfNb0.5TiV0.5Zr | BCC | 25.0 | 0.0 | 25.0 | 0.0 | 0 | 0 | 0 | 0 |
| AlCoCrFe6NiSiTi | BCC | 25.0 | 16.7 | 8.3 | 0.0 | 0 | 1 | 0 | 8.33 |
| CoFeMnNi | FCC | 25.0 | 25.0 | 0.0 | 0.0 | 0 | 0 | 0 | 0 |
| PbSnTeSe | FCC | 25.0 | 25.0 | 0.0 | 0.0 | 0 | 0 | 0 | 0 |
| Co25Cr25Fe25Mn25 | FCC | 25.0 | 0.0 | 25.0 | 0.0 | 0 | 0 | 1 | 0 |
| Al7.5Cr6Fe40.4Mn34.8Ni11.3 | FCC | 24.8 | 18.8 | 6.0 | 0.0 | 0 | 0 | 0 | 7.5 |
| Al7.4C1.1Cr5.55Fe39.93Mn35.67Ni10.35 | FCC | 23.3 | 17.8 | 5.6 | 0.0 | 0 | 0 | 0 | 7.4 |
| Pb0.9SnTeSeLa0.1 | FCC | 22.5 | 22.5 | 0.0 | 0.0 | 0 | 0 | 0 | 0 |
| DyGdHoTbY | HCP | 20.0 | 0.0 | 0.0 | 20.0 | 1 | 0 | 0 | 0 |
| DyGdLuTbY | HCP | 20.0 | 0.0 | 0.0 | 20.0 | 1 | 0 | 0 | 0 |
| GdHoLaTbY | HCP | 20.0 | 0.0 | 0.0 | 20.0 | 1 | 0 | 0 | 0 |
| BiSbTe1.5Se1.5 | RHOM | 20.0 | 0.0 | 0.0 | 0.0 | 1 | 0 | 0 | 0 |
| CoCuFeTiZrHf | FCC | 16.7 | 16.7 | 0.0 | 0.0 | 0 | 0 | 0 | 0 |
| HfTa0.53TiZr | BCC | 15.0 | 0.0 | 15.0 | 0.0 | 0 | 0 | 0 | 0 |
| B2(HfTaTiVZr)1 | HCP | 13.3 | 0.0 | 13.3 | 0.0 | 0 | 0 | 0 | 0 |
| Hf27.5Nb5Ta5Ti35Zr27.5 | BCC | 10.0 | 0.0 | 10.0 | 0.0 | 0 | 0 | 0 | 0 |
| Co10Cr10Fe40Mn40 | FCC | 10.0 | 0.0 | 10.0 | 0.0 | 0 | 0 | 1 | 0 |
| CoFeMnTi0.5V0.4Zr0.4 | Laves C14 | 9.3 | 0.0 | 9.3 | 0.0 | 0 | 0 | 0 | 0 |
| Ni5(CoFeMn)95 | FCC | 5.0 | 5.0 | 0.0 | 0.0 | 0 | 0 | 0 | 0 |
| C69.23(Co10Cr10Fe40Mn40)30.77 | FCC | 3.2 | 0.0 | 3.2 | 0.0 | 0 | 0 | 1 | 0 |
| C77.34(Co10Cr10Fe40Mn40)22.66 | FCC | 2.9 | 0.0 | 2.9 | 0.0 | 0 | 0 | 1 | 0 |
| C82.15(Co10Cr10Fe40Mn40)17.85 | FCC | 2.7 | 0.0 | 2.7 | 0.0 | 0 | 0 | 1 | 0 |
| C87.60(Co10Cr10Fe40Mn40)12.4 | FCC | 2.6 | 0.0 | 2.6 | 0.0 | 0 | 0 | 1 | 0 |
| C90.71(Co10Cr10Fe40Mn40)9.29 | FCC | 2.5 | 0.0 | 2.5 | 0.0 | 0 | 0 | 1 | 0 |

**Supplementary Table 3. Analysis of HEAs using traditional descriptors from Gorsse *et al*.** The experimentally observed phase, valence electron concentration (VEC), electron per atom (e/a) ratio, mixing enthalpy (${\Delta H}_{mix}$), atomic size mismatch (δ), and the atom percent of non-allotrope FCC and BCC elements is detailed for each composition. The phase predicted by VEC and e/a ratio are reported for comparison with the experimental crystal structure.

| Composition  (atom %) | Phase | VEC | VEC Predicted Phase | e/a Ratio | e/a Ratio  Predicted Phase | $\boldsymbol{\Delta}\boldsymbol{H}_{\boldsymbol{mix}}$  (kJ/mol) | Atomic Size Mismatch δ (%) | FCC  (atom %) | BCC  (atom %) |
| --- | --- | --- | --- | --- | --- | --- | --- | --- | --- |
| MoNbTaV | BCC | 5.25 | BCC | 1.31 | Uncertain | -6.6 | 6.04 | 0.0 | 100.0 |
| MoNbTaVW | BCC | 5.40 | BCC | 1.08 | Uncertain | -13.6 | 5.42 | 0.0 | 100.0 |
| MoNbTaW | BCC | 5.50 | BCC | 1.38 | Uncertain | -16.1 | 2.03 | 0.0 | 100.0 |
| NbTaVW | BCC | 5.25 | BCC | 1.31 | Uncertain | -9.6 | 6.06 | 0.0 | 100.0 |
| Al0.8CrCuFeNi2 | FCC | 8.17 | FCC | 1.41 | Uncertain | -26.9 | 9.37 | 65.5 | 17.2 |
| Al0.6CrCuFeNi2 | FCC | 8.36 | FCC | 1.49 | Uncertain | -23.7 | 8.64 | 64.3 | 17.9 |
| Al0.4CrCuFeNi2 | FCC | 8.56 | FCC | 1.58 | Uncertain | -19.0 | 7.72 | 63.0 | 18.5 |
| Al0.2CrCuFeNi2 | FCC | 8.77 | FCC | 1.69 | FCC | -11.9 | 6.53 | 61.5 | 19.2 |
| CrCuFeMoNi | FCC | 8.20 | FCC | 1.64 | FCC | 5.5 | 9.97 | 40.0 | 40.0 |
| AlMoNbTiV | BCC | 4.60 | BCC | 0.92 | Uncertain | -48.8 | 16.42 | 20.0 | 60.0 |
| AlNbTaTiV | BCC | 4.40 | BCC | 0.88 | Uncertain | -50.7 | 17.17 | 20.0 | 60.0 |
| Al0.75MoNbTiV | BCC | 4.68 | BCC | 0.99 | Uncertain | -45.3 | 14.96 | 15.8 | 63.2 |
| Al22.5Cu20Fe15Ni20Ti2 | FCC | 7.74 | Uncertain | 0.10 | Uncertain | -35.1 | 11.04 | 78.6 | 0.0 |
| Al0.5MoNbTiV | BCC | 4.78 | BCC | 1.06 | Uncertain | -42.5 | 13.05 | 11.1 | 66.7 |
| Al0.5NbTaTiV | BCC | 4.56 | BCC | 1.01 | Uncertain | -44.0 | 13.80 | 11.1 | 66.7 |
| AlCrFeNiMo0.5 | BCC | 6.67 | BCC | 1.48 | Uncertain | -50.2 | 14.21 | 44.4 | 33.3 |
| AlCr0.5NbTiV | BCC | 4.44 | BCC | 0.99 | Uncertain | -52.9 | 16.71 | 22.2 | 55.6 |
| Al0.25MoNbTiV | BCC | 4.88 | BCC | 1.15 | Uncertain | -31.6 | 10.38 | 5.9 | 70.6 |
| Al0.25NbTaTiV | BCC | 4.65 | BCC | 1.09 | Uncertain | -27.8 | 11.17 | 5.9 | 70.6 |
| AlCrFeNiMo0.2 | BCC | 6.71 | BCC | 1.60 | Uncertain | -49.1 | 13.22 | 47.6 | 28.6 |
| MoNbTiV | BCC | 5.00 | BCC | 1.25 | Uncertain | -4.8 | 5.87 | 0.0 | 75.0 |
| NbTaTiV | BCC | 4.75 | BCC | 1.19 | Uncertain | 1.2 | 6.92 | 0.0 | 75.0 |
| Zn25(CuMnNi)75 | FCC | 11.71 | FCC | 0.42 | Uncertain | -16.5 | 2.61 | 50.0 | 0.0 |
| AlCrFeNi | BCC | 6.75 | BCC | 1.69 | Uncertain | -46.5 | 12.18 | 50.0 | 25.0 |
| AlCuNiTi | FCC | 7.00 | Uncertain | 1.75 | Uncertain | -63.2 | 13.98 | 75.0 | 0.0 |
| Al3CoCrCuFeNi | BCC | 6.63 | BCC | 0.83 | Uncertain | -33.8 | 12.94 | 62.5 | 12.5 |
| Al2.8CoCrCuFeNi | BCC | 6.72 | BCC | 0.86 | Uncertain | -34.2 | 12.79 | 61.5 | 12.8 |
| NbTiV0.3Mo1.5 | BCC | 5.13 | BCC | 1.35 | Uncertain | -6.7 | 5.00 | 0.0 | 73.7 |
| Zn20(CuMnNi)80 | FCC | 11.65 | FCC | 0.51 | Uncertain | -15.1 | 2.85 | 53.3 | 0.0 |
| Al0.5CoCrCuFeNiV2.0 | BCC | 7.40 | Uncertain | 0.99 | Uncertain | -39.9 | 8.95 | 33.3 | 40.0 |
| Al0.5CoCrCuFeNiV1.8 | BCC | 7.47 | Uncertain | 1.02 | Uncertain | -40.5 | 8.94 | 34.2 | 38.4 |
| NbTiV0.3Mo1.3 | BCC | 5.08 | BCC | 1.41 | Uncertain | -6.3 | 5.13 | 0.0 | 72.2 |
| Al0.5CoCrCuFeNiV1.6 | BCC | 7.54 | Uncertain | 1.06 | Uncertain | -41.1 | 8.93 | 35.2 | 36.6 |
| MoNbTiV3.0Zr | BCC | 4.86 | BCC | 0.69 | Uncertain | -2.5 | 7.39 | 0.0 | 71.4 |
| Al3CoCrFeNi | BCC | 6.00 | BCC | 0.86 | Uncertain | -44.1 | 13.83 | 57.1 | 14.3 |
| Al3.0CoCrCuFe | BCC | 6.14 | BCC | 0.88 | Uncertain | -23.9 | 13.72 | 57.1 | 14.3 |
| Al0.5CoCrCuFeNiV1.4 | BCC | 7.61 | Uncertain | 1.10 | Uncertain | -41.5 | 8.91 | 36.2 | 34.8 |
| Al2.8CoCrCuFe | BCC | 6.24 | BCC | 0.92 | Uncertain | -24.2 | 13.61 | 55.9 | 14.7 |
| Al0.5CoCrCuFeNiV1.2 | BCC | 7.69 | Uncertain | 1.15 | Uncertain | -41.8 | 8.88 | 37.3 | 32.8 |
| NbTiV0.3Mo | BCC | 5.00 | BCC | 1.52 | Uncertain | -5.6 | 5.34 | 0.0 | 69.7 |
| Al2CoCrFeMo0.5Ni | BCC | 6.46 | BCC | 0.99 | Uncertain | -53.3 | 14.83 | 46.2 | 23.1 |
| Al2.5CoCrFeNi | BCC | 6.23 | BCC | 0.96 | Uncertain | -46.0 | 13.48 | 53.8 | 15.4 |
| Al5(CuMnNi)95 | FCC | 5.38 | BCC | 0.67 | Uncertain | -12.7 | 12.88 | 68.3 | 0.0 |
| CoCrNi | FCC | 8.33 | FCC | 2.78 | Uncertain | -10.8 | 4.76 | 33.3 | 33.3 |
| Mo2NbTiVZr | BCC | 5.00 | BCC | 0.83 | Uncertain | -6.3 | 6.36 | 0.0 | 66.7 |
| MoNbTiV2.0Zr | BCC | 4.83 | BCC | 0.81 | Uncertain | -3.3 | 7.33 | 0.0 | 66.7 |
| NbTiV0.3Mo0.7 | BCC | 4.90 | BCC | 1.63 | Uncertain | -4.2 | 5.57 | 0.0 | 66.7 |
| AlCoCrCuNiTi | BCC | 7.17 | Uncertain | 1.19 | Uncertain | -70.8 | 12.03 | 50.0 | 16.7 |
| Al2CoCrFeNi | BCC | 6.50 | BCC | 1.08 | Uncertain | -48.7 | 12.96 | 50.0 | 16.7 |
| Mo1.7NbTiVZr | BCC | 4.95 | BCC | 0.87 | Uncertain | -5.8 | 6.53 | 0.0 | 64.9 |
| Al0.5CoCrCuFeNiV0.2 | FCC | 8.16 | FCC | 1.43 | Uncertain | -33.4 | 8.39 | 43.9 | 21.1 |
| NbTiV0.3Mo0.5 | BCC | 4.82 | BCC | 1.72 | Uncertain | -3.0 | 5.75 | 0.0 | 64.3 |
| Mo1.5NbTiVZr | BCC | 4.91 | BCC | 0.89 | Uncertain | -5.4 | 6.65 | 0.0 | 63.6 |
| MoNbTiV1.5Zr | BCC | 4.82 | BCC | 0.88 | Uncertain | -3.7 | 7.21 | 0.0 | 63.6 |
| Al0.5CoCrCuFeNi | FCC | 8.27 | FCC | 1.50 | Uncertain | -26.3 | 8.19 | 45.5 | 18.2 |
| Al1.5CoCrFeNi | BCC | 6.82 | BCC | 1.24 | Uncertain | -51.0 | 12.16 | 45.5 | 18.2 |
| Al1.125CuFe0.75NiTi1.1 | FCC | 6.99 | Uncertain | 1.41 | Uncertain | -68.3 | 13.30 | 62.8 | 0.0 |
| Mo1.3NbTiVZr | BCC | 4.87 | BCC | 0.92 | Uncertain | -4.9 | 6.77 | 0.0 | 62.3 |
| Al0.3CoCrCuFeNi | FCC | 8.47 | FCC | 1.60 | Uncertain | -19.6 | 7.11 | 43.4 | 18.9 |
| Al1.25CoCrFeNi | BCC | 7.00 | Uncertain | 1.33 | Uncertain | -51.6 | 11.60 | 42.9 | 19.0 |
| NbTiV0.3Mo0.3 | BCC | 4.73 | BCC | 1.82 | BCC | -1.8 | 5.94 | 0.0 | 61.5 |
| Al0.5CoCrCuFeNiTi0.2 | FCC | 8.12 | FCC | 1.43 | Uncertain | -42.7 | 8.58 | 43.9 | 17.5 |
| AlCoCrFeMo0.1Ni | BCC | 7.18 | Uncertain | 1.41 | Uncertain | -52.8 | 11.42 | 39.2 | 21.6 |
| AlCoCrFeNb0.1Ni | BCC | 7.16 | Uncertain | 1.40 | Uncertain | -58.9 | 11.68 | 39.2 | 21.6 |
| CoCrCuFeNi | FCC | 8.80 | FCC | 1.76 | FCC | 0.5 | 4.67 | 40.0 | 20.0 |
| MoNbTiVZr | BCC | 4.80 | BCC | 0.96 | Uncertain | -3.9 | 6.97 | 0.0 | 60.0 |
| MoNbTiV1.0Zr | BCC | 4.80 | BCC | 0.96 | Uncertain | -3.9 | 6.97 | 0.0 | 60.0 |
| NbTiV2Zr | BCC | 4.60 | BCC | 0.92 | Uncertain | 2.7 | 7.98 | 0.0 | 60.0 |
| AlCoCrFeNi | BCC | 7.20 | Uncertain | 1.44 | Uncertain | -51.2 | 10.90 | 40.0 | 20.0 |
| AlCuFeNiTi | FCC | 7.20 | Uncertain | 1.44 | Uncertain | -66.7 | 12.59 | 60.0 | 0.0 |
| AlMo0.5NbTa0.5TiZr | BCC | 4.30 | BCC | 0.86 | Uncertain | -65.6 | 17.88 | 20.0 | 40.0 |
| AlNb1.5Ta0.5Ti1.5Zr0.5 | BCC | 4.20 | BCC | 0.84 | Uncertain | -57.4 | 17.61 | 20.0 | 40.0 |
| AlNBTiV | BCC | 4.00 | BCC | 0.80 | Uncertain | 17.3 | 38.42 | 20.0 | 20.0 |
| Al20(CoCrCuFeMnNiTiV)80 | BCC | 4.29 | BCC | 0.15 | Uncertain | -57.9 | 15.04 | 40.0 | 20.0 |
| Al0.5CoCrCuFeNiTi0.4 | FCC | 7.98 | Uncertain | 1.35 | Uncertain | -51.9 | 8.89 | 42.4 | 16.9 |
| NbTiV0.3Mo0.1 | BCC | 4.63 | BCC | 1.93 | BCC | 0.0 | 6.15 | 0.0 | 58.3 |
| MoNbTiV0.75Zr | BCC | 4.79 | BCC | 1.01 | Uncertain | -4.0 | 6.79 | 0.0 | 57.9 |
| AlCoCrFeNiSi0.2 | BCC | 7.08 | Uncertain | 1.36 | Uncertain | -59.0 | 11.84 | 38.5 | 19.2 |
| Mo0.7NbTiVZr | BCC | 4.72 | BCC | 1.01 | Uncertain | -3.0 | 7.19 | 0.0 | 57.4 |
| Al2CoCrFeNiTi | BCC | 6.14 | BCC | 0.88 | Uncertain | -87.5 | 13.98 | 42.9 | 14.3 |
| CoCrCu0.5FeNi | FCC | 8.56 | FCC | 1.90 | Uncertain | -4.4 | 4.48 | 33.3 | 22.2 |
| Mo0.5NbTiVZr | BCC | 4.67 | BCC | 1.04 | Uncertain | -2.1 | 7.35 | 0.0 | 55.6 |
| MoNbTiV0.50Zr | BCC | 4.78 | BCC | 1.06 | Uncertain | -3.8 | 6.54 | 0.0 | 55.6 |
| Al0.5CoCrCuFe | FCC | 7.89 | Uncertain | 1.75 | FCC | -20.2 | 9.02 | 33.3 | 22.2 |
| AlCoCrFeNiSi0.4 | BCC | 6.96 | Uncertain | 1.29 | Uncertain | -61.0 | 12.61 | 37.0 | 18.5 |
| CoCrCuFeNiTi0.5 | FCC | 8.36 | FCC | 1.52 | Uncertain | -24.4 | 6.04 | 36.4 | 18.2 |
| AlCoCrFeNiTi0.5 | FCC | 6.91 | Uncertain | 1.26 | Uncertain | -80.7 | 11.51 | 36.4 | 18.2 |
| Al0.375CoCrFeNi | FCC | 7.80 | Uncertain | 1.78 | FCC | -40.6 | 8.01 | 31.4 | 22.9 |
| Al1.5CoCrFeNiTi | BCC | 6.39 | BCC | 0.98 | Uncertain | -89.6 | 13.08 | 38.5 | 15.4 |
| AlCoCrFeNiSi0.6 | BCC | 6.86 | BCC | 1.22 | Uncertain | -59.8 | 13.26 | 35.7 | 17.9 |
| Mo0.3NbTiVZr | BCC | 4.61 | BCC | 1.07 | Uncertain | -0.7 | 7.52 | 0.0 | 53.5 |
| Al0.3CoCrCuFe | FCC | 8.12 | FCC | 1.89 | Uncertain | -14.0 | 7.81 | 30.2 | 23.3 |
| MoNbTiV0.25Zr | BCC | 4.77 | BCC | 1.12 | Uncertain | -3.3 | 6.21 | 0.0 | 52.9 |
| Al0.25CoCrFeNi | FCC | 7.94 | Uncertain | 1.87 | Uncertain | -35.0 | 7.07 | 29.4 | 23.5 |
| Al1.25CoCrFeMnNi | BCC | 7.00 | Uncertain | 1.12 | Uncertain | -61.6 | 11.04 | 36.0 | 16.0 |
| CoCrFeNi | FCC | 8.25 | FCC | 2.06 | Uncertain | -15.0 | 4.12 | 25.0 | 25.0 |
| CoCrMnNi | FCC | 8.00 | FCC | 2.00 | Uncertain | -17.0 | 4.34 | 25.0 | 25.0 |
| CoCrCuFe | FCC | 8.50 | FCC | 2.13 | Uncertain | 3.8 | 4.91 | 25.0 | 25.0 |
| CoCrCuFeNiTi | FCC | 8.00 | Uncertain | 1.33 | Uncertain | -31.8 | 6.74 | 33.3 | 16.7 |
| CoCuFeNi | FCC | 9.50 | FCC | 2.38 | Uncertain | 4.4 | 2.68 | 50.0 | 0.0 |
| HfMoNbTaTiZr | BCC | 4.67 | BCC | 0.78 | Uncertain | -0.8 | 5.50 | 0.0 | 50.0 |
| HfNbTaZr | BCC | 4.50 | BCC | 1.13 | Uncertain | 8.4 | 2.03 | 0.0 | 50.0 |
| MoNbTiZr | BCC | 4.75 | BCC | 1.19 | Uncertain | -2.4 | 5.76 | 0.0 | 50.0 |
| NbTiVZr | BCC | 4.50 | BCC | 1.13 | Uncertain | 2.8 | 7.80 | 0.0 | 50.0 |
| CoCrCuFeNiTiVZr | FCC | 7.13 | Uncertain | 0.89 | Uncertain | -65.8 | 11.22 | 25.0 | 25.0 |
| CoFeNiV | FCC | 8.00 | FCC | 2.00 | Uncertain | -27.8 | 5.39 | 25.0 | 25.0 |
| Al0.25CoCrCu0.75FeNiTi | FCC | 7.67 | Uncertain | 1.28 | Uncertain | -54.3 | 8.33 | 33.3 | 16.7 |
| Al0.5NbTa0.8Ti1.5V0.2Zr | BCC | 4.30 | BCC | 0.86 | Uncertain | -48.3 | 13.72 | 10.0 | 40.0 |
| AlCoCrFeNiSi | BCC | 6.67 | BCC | 1.11 | Uncertain | -53.0 | 14.25 | 33.3 | 16.7 |
| AlCoCrFeNiTi | BCC | 6.67 | BCC | 1.11 | Uncertain | -88.2 | 11.79 | 33.3 | 16.7 |
| AlFeNiTiVZr | BCC | 5.67 | BCC | 0.94 | Uncertain | -113.0 | 16.56 | 33.3 | 16.7 |
| AlCoFeNi | BCC | 7.50 | Uncertain | 1.88 | Uncertain | -42.8 | 10.49 | 50.0 | 0.0 |
| CoCuFeNiSn0.02 | FCC | 9.47 | FCC | 2.36 | Uncertain | 4.0 | 2.69 | 49.8 | 0.0 |
| Al0.4Hf0.6NbTaTiZr | BCC | 4.32 | BCC | 0.86 | Uncertain | -43.4 | 12.61 | 8.0 | 40.0 |
| HfMo0.75NbTaTiZr | BCC | 4.61 | BCC | 0.80 | Uncertain | 0.2 | 5.57 | 0.0 | 47.8 |
| Al0.25CoCrCu0.5FeNiTi | FCC | 7.52 | Uncertain | 1.31 | Uncertain | -57.3 | 8.35 | 30.4 | 17.4 |
| Al0.75HfNbTaTiZr | BCC | 4.22 | BCC | 0.73 | Uncertain | -64.3 | 15.41 | 13.0 | 34.8 |
| Al0.2Co1.5CrFeNi1.5Ti0.5 | FCC | 7.91 | Uncertain | 1.39 | Uncertain | -53.1 | 7.05 | 29.8 | 17.5 |
| Al0.3NbTa0.8Ti1.4V0.2Zr1.3 | BCC | 4.34 | BCC | 0.87 | Uncertain | -30.6 | 11.55 | 6.0 | 40.0 |
| Al0.3NbTaTi1.4Zr1.3 | BCC | 4.34 | BCC | 0.87 | Uncertain | -27.3 | 11.38 | 6.0 | 40.0 |
| Co1.5CrFeNi1.5Ti0.5 | FCC | 8.09 | FCC | 1.47 | Uncertain | -36.6 | 5.41 | 27.3 | 18.2 |
| CoCrFeMnNiV0.5 | FCC | 7.73 | Uncertain | 1.41 | Uncertain | -26.0 | 4.50 | 18.2 | 27.3 |
| HfMo0.5NbTaTiZr | BCC | 4.55 | BCC | 0.83 | Uncertain | 1.7 | 5.64 | 0.0 | 45.5 |
| Al0.5HfNbTaTiZr | BCC | 4.27 | BCC | 0.78 | Uncertain | -48.8 | 13.31 | 9.1 | 36.4 |
| Al0.5CrFe1.5MnNi0.5 | BCC | 7.00 | Uncertain | 1.56 | Uncertain | -45.6 | 8.95 | 22.2 | 22.2 |
| Al0.38CoCrFeMnNi | FCC | 7.65 | Uncertain | 1.42 | Uncertain | -46.7 | 7.50 | 25.7 | 18.6 |
| Al0.3HfNbTaTiZr | BCC | 4.32 | BCC | 0.82 | Uncertain | -31.1 | 11.12 | 5.7 | 37.7 |
| CoCrFeMnNiV0.25 | FCC | 7.86 | Uncertain | 1.50 | Uncertain | -23.7 | 4.25 | 19.0 | 23.8 |
| HfMo0.25NbTaTiZr | BCC | 4.48 | BCC | 0.85 | Uncertain | 4.1 | 5.71 | 0.0 | 42.9 |
| AlCoFeNiTiVZr | BCC | 6.14 | BCC | 0.88 | Uncertain | -130.0 | 15.65 | 28.6 | 14.3 |
| Al0.20CoCrFeMnNi | FCC | 7.81 | Uncertain | 1.50 | Uncertain | -36.7 | 6.16 | 23.1 | 19.2 |
| Al0.10CoCrFeMnNi | FCC | 7.90 | Uncertain | 1.55 | Uncertain | -28.9 | 5.19 | 21.6 | 19.6 |
| CoCrFeNiTi | FCC | 7.40 | Uncertain | 1.48 | Uncertain | -42.6 | 6.21 | 20.0 | 20.0 |
| CoCrFeMnNi | FCC | 8.00 | FCC | 1.60 | Uncertain | -19.0 | 3.90 | 20.0 | 20.0 |
| CoCuFeMnNi | FCC | 9.00 | FCC | 1.80 | Uncertain | -5.5 | 3.63 | 40.0 | 0.0 |
| HfMoTaTiZr | BCC | 4.60 | BCC | 0.92 | Uncertain | -2.3 | 6.02 | 0.0 | 40.0 |
| HfMoNbZrTi | BCC | 4.60 | BCC | 0.92 | Uncertain | -1.5 | 5.98 | 0.0 | 40.0 |
| HfNbTaTiZr | BCC | 4.40 | BCC | 0.88 | Uncertain | 8.0 | 5.78 | 0.0 | 40.0 |
| CoCuFeMnNiSn0.03 | FCC | 8.97 | FCC | 1.78 | FCC | -6.7 | 3.64 | 39.8 | 0.0 |
| NbTiV0.3Zr | BCC | 4.39 | BCC | 1.33 | Uncertain | 4.1 | 7.16 | 0.0 | 39.4 |
| Al0.25CoFeNi | FCC | 8.54 | FCC | 2.63 | Uncertain | -29.1 | 6.38 | 38.5 | 0.0 |
| CoFeNi | FCC | 9.00 | FCC | 3.00 | Uncertain | -10.4 | 1.88 | 33.3 | 0.0 |
| CoMnNi | FCC | 8.67 | FCC | 2.89 | Uncertain | -17.0 | 3.31 | 33.3 | 0.0 |
| FeMnNi | FCC | 8.33 | FCC | 2.78 | Uncertain | -20.8 | 3.17 | 33.3 | 0.0 |
| CoFeNiSi0.25 | FCC | 8.62 | FCC | 2.65 | Uncertain | -23.8 | 7.61 | 30.8 | 0.0 |
| CoFeMnNi | FCC | 8.50 | FCC | 2.13 | Uncertain | -19.5 | 2.91 | 25.0 | 0.0 |
| Hf0.5Nb0.5Ta0.5Ti1.5Zr | BCC | 4.25 | BCC | 1.06 | Uncertain | 6.7 | 7.09 | 0.0 | 25.0 |
| HfNbTiZr | BCC | 4.25 | BCC | 1.06 | Uncertain | 6.5 | 6.44 | 0.0 | 25.0 |

**Supplementary Table 4. Analysis of HEAs using traditional descriptors from Murty *et al*.** The experimentally observed phase, valence electron concentration (VEC), electron per atom (e/a) ratio, mixing enthalpy (${\Delta H}_{mix}$), atomic size mismatch (δ), and the atom percent of non-allotrope FCC and BCC elements is detailed for each composition. The phase predicted by VEC and e/a ratio are reported for comparison with the experimental crystal structure.

| Composition  (atom %) | Phase | VEC | VEC Predicted Phase | e/a Ratio | e/a Ratio  Predicted Phase | $\boldsymbol{\Delta}\boldsymbol{H}_{\boldsymbol{mix}}$  (kJ/mol) | Atomic Size Mismatch δ (%) | FCC  (atom %) | BCC  (atom %) |
| --- | --- | --- | --- | --- | --- | --- | --- | --- | --- |
| MoNbTaW | BCC | 5.50 | BCC | 1.38 | Uncertain | -16.1 | 2.03 | 0.0 | 100.0 |
| NbTaVW | BCC | 5.25 | BCC | 1.31 | Uncertain | -9.6 | 6.06 | 0.0 | 100.0 |
| MoNbTaVW | BCC | 5.40 | BCC | 1.08 | Uncertain | -13.6 | 5.42 | 0.0 | 100.0 |
| CrMoNbReTaVW | BCC | 5.71 | BCC | 0.82 | Uncertain | -36.4 | 6.51 | 0.0 | 85.7 |
| Cr0.5MoNbTaVW | BCC | 5.46 | BCC | 0.99 | Uncertain | -15.9 | 6.42 | 0.0 | 100.0 |
| CrMoNbTaVW | BCC | 5.50 | BCC | 0.92 | Uncertain | -16.1 | 7.03 | 0.0 | 100.0 |
| AgAuPdPt | FCC | 10.50 | FCC | 2.63 | Uncertain | -3.2 | 2.69 | 100.0 | 0.0 |
| AuCuNiPd | FCC | 10.50 | FCC | 2.63 | Uncertain | -3.1 | 7.82 | 100.0 | 0.0 |
| AuCuNiPt | FCC | 10.50 | FCC | 2.63 | Uncertain | -2.2 | 8.91 | 100.0 | 0.0 |
| AuCuPdPt | FCC | 10.50 | FCC | 2.63 | Uncertain | -12.5 | 7.58 | 100.0 | 0.0 |
| AuNiPdPt | FCC | 10.25 | FCC | 2.56 | Uncertain | 7.1 | 6.53 | 100.0 | 0.0 |
| CuNiPdPt | FCC | 10.25 | FCC | 2.56 | Uncertain | -10.3 | 8.36 | 100.0 | 0.0 |
| AuCuNiPdPt | FCC | 10.40 | FCC | 2.08 | Uncertain | -6.8 | 8.12 | 100.0 | 0.0 |
| CuIrNiPdPtRh | FCC | 9.83 | FCC | 1.64 | FCC | -6.2 | 8.19 | 100.0 | 0.0 |
| Ir0.26Os0.05Pt0.31Rh0.23Ru0.15 | FCC | 9.11 | FCC | 9.11 | Uncertain | -1.1 | 1.71 | 80.0 | 0.0 |
| MoNbTaTi0.25W | BCC | 5.41 | BCC | 1.27 | Uncertain | -16.1 | 3.06 | 0.0 | 94.1 |
| Co2Cr2Fe2Mn2Ni92 | FCC | 9.80 | FCC | 0.10 | Uncertain | -3.0 | 2.03 | 92.0 | 2.0 |
| MoNbTaTi0.5W | BCC | 5.33 | BCC | 1.19 | Uncertain | -15.8 | 3.68 | 0.0 | 88.9 |
| Al2CrCuFeNi2 | BCC | 7.29 | Uncertain | 1.04 | Uncertain | -32.0 | 11.89 | 71.4 | 14.3 |
| MoNbTaTi0.75W | BCC | 5.26 | BCC | 1.11 | Uncertain | -15.2 | 4.11 | 0.0 | 84.2 |
| AlCrMoNbTiV | BCC | 4.83 | BCC | 0.81 | Uncertain | -51.9 | 15.09 | 16.7 | 66.7 |
| MoNbTaTiVW | BCC | 5.17 | BCC | 0.86 | Uncertain | -13.4 | 5.77 | 0.0 | 83.3 |
| Al1.5MoNbTiV | BCC | 4.46 | BCC | 0.81 | Uncertain | -52.6 | 18.51 | 27.3 | 54.5 |
| Al0.5CrMoNbTiV | BCC | 5.00 | BCC | 0.91 | Uncertain | -44.6 | 12.15 | 9.1 | 72.7 |
| Al0.5CrCuFeNi2 | FCC | 8.46 | FCC | 1.54 | Uncertain | -21.6 | 8.20 | 63.6 | 18.2 |
| AlCrCuFeNi | BCC | 7.60 | Uncertain | 1.52 | Uncertain | -29.4 | 10.95 | 60.0 | 20.0 |
| AlCrMoNbTi | BCC | 4.80 | BCC | 0.96 | Uncertain | -49.4 | 16.55 | 20.0 | 60.0 |
| MoNbTaTiW | BCC | 5.20 | BCC | 1.04 | Uncertain | -14.7 | 4.43 | 0.0 | 80.0 |
| NbTaTiVW | BCC | 5.00 | BCC | 1.00 | Uncertain | -8.9 | 6.31 | 0.0 | 80.0 |
| AlMoTaTiV | BCC | 4.60 | BCC | 0.92 | Uncertain | -49.0 | 16.62 | 20.0 | 60.0 |
| AlCoCuNiZn | FCC | 9.00 | FCC | 1.80 | Uncertain | -36.5 | 8.56 | 60.0 | 0.0 |
| CrCuFeMoNi | FCC | 8.20 | FCC | 1.64 | FCC | 5.5 | 9.97 | 40.0 | 40.0 |
| CrNbTiVZn | FCC | 6.40 | BCC | 1.28 | Uncertain | -22.0 | 10.55 | 0.0 | 60.0 |
| CrCuFeNi2 | FCC | 9.00 | FCC | 1.80 | Uncertain | -1.0 | 4.84 | 60.0 | 20.0 |
| O(CoCuMgNiZn)50 | FCC | 8.33 | FCC | 1.39 | Uncertain | 0.0 | 28.34 | 39.2 | 0.0 |
| AlCoCrCu0.5Ni | BCC | 7.44 | Uncertain | 1.65 | Uncertain | -36.5 | 11.31 | 55.6 | 22.2 |
| Al0.5CrMoNbTi | BCC | 5.00 | BCC | 1.11 | Uncertain | -41.1 | 13.34 | 11.1 | 66.7 |
| AlCr0.5NbTiV | BCC | 4.44 | BCC | 0.99 | Uncertain | -52.9 | 16.71 | 22.2 | 55.6 |
| Al0.25MoNbTiV | BCC | 4.88 | BCC | 1.15 | Uncertain | -31.6 | 10.38 | 5.9 | 70.6 |
| Al0.2MoTaTiV | BCC | 4.91 | BCC | 1.17 | Uncertain | -27.1 | 9.94 | 4.8 | 71.4 |
| AlNbTiV | BCC | 4.25 | BCC | 1.06 | Uncertain | -48.1 | 17.73 | 25.0 | 50.0 |
| CrFeMoV | BCC | 6.25 | BCC | 1.56 | Uncertain | -6.0 | 7.24 | 0.0 | 75.0 |
| MoTaTiV | BCC | 5.00 | BCC | 1.25 | Uncertain | -4.6 | 6.22 | 0.0 | 75.0 |
| NbTaTiV | BCC | 4.75 | BCC | 1.19 | Uncertain | 1.2 | 6.92 | 0.0 | 75.0 |
| CrMoNbTaTiVWZr | BCC | 5.13 | BCC | 0.64 | Uncertain | -20.6 | 7.33 | 0.0 | 75.0 |
| AlCuTiNi | FCC | 7.00 | Uncertain | 1.75 | Uncertain | -63.2 | 13.98 | 75.0 | 0.0 |
| CoCrCuNi | FCC | 9.00 | FCC | 2.25 | Uncertain | 1.7 | 5.17 | 50.0 | 25.0 |
| CoCuNiZn | FCC | 10.50 | FCC | 2.63 | Uncertain | -10.5 | 2.59 | 50.0 | 0.0 |
| Ni50(AlCoCrFe)50 | FCC | 9.74 | FCC | 0.18 | Uncertain | -51.2 | 3.30 | 62.5 | 12.5 |
| CrCuFeNi | FCC | 8.75 | FCC | 2.19 | Uncertain | 0.3 | 5.17 | 50.0 | 25.0 |
| Al0.85CuFeNi | BCC | 8.20 | FCC | 2.13 | Uncertain | -24.9 | 9.70 | 74.0 | 0.0 |
| Al0.6MoTaTi | BCC | 4.67 | BCC | 1.30 | Uncertain | -39.9 | 15.73 | 16.7 | 55.6 |
| AlCoCrCuFeNiW | BCC | 7.57 | Uncertain | 1.08 | Uncertain | -29.5 | 13.60 | 42.9 | 28.6 |
| CrMoNbTaTiVZr | BCC | 5.00 | BCC | 0.71 | Uncertain | -13.7 | 7.78 | 0.0 | 71.4 |
| Al2CoCrCuFeNi | BCC | 7.14 | Uncertain | 1.02 | Uncertain | -35.3 | 11.98 | 57.1 | 14.3 |
| Al0.5CuFeNi | FCC | 8.71 | FCC | 2.49 | Uncertain | -20.1 | 8.23 | 71.4 | 0.0 |
| Al1.67CoCrCuFeNi | BCC | 7.35 | Uncertain | 1.10 | Uncertain | -35.9 | 11.49 | 55.0 | 15.0 |
| Al0.3CuFeNi | FCC | 9.06 | FCC | 2.75 | Uncertain | -14.3 | 6.91 | 69.7 | 0.0 |
| Al0.5CoCrCuFeNiV | BCC | 7.77 | Uncertain | 1.20 | Uncertain | -41.7 | 8.83 | 38.5 | 30.8 |
| Al2.3B0.15CoCrCu0.7FeNiSi0.1 | BCC | 6.68 | BCC | 0.92 | Uncertain | -45.3 | 13.94 | 55.2 | 13.8 |
| Al1.25CoCrCuFeNi | BCC | 7.64 | Uncertain | 1.22 | Uncertain | -35.4 | 10.66 | 52.0 | 16.0 |
| AlCoCrCuFeNiV0.2 | FCC | 7.74 | Uncertain | 1.25 | Uncertain | -40.6 | 10.19 | 48.4 | 19.4 |
| Al2.3B0.3CoCrCu0.7FeNiSi0.1 | BCC | 6.61 | BCC | 0.89 | Uncertain | -47.9 | 14.93 | 54.1 | 13.5 |
| Al0.7Co0.3CrFeNi | BCC | 7.20 | Uncertain | 1.80 | Uncertain | -49.4 | 10.61 | 42.5 | 25.0 |
| Hf8Nb33Ta34 Ti11Zr14 | BCC | 4.67 | BCC | 0.05 | Uncertain | 6.5 | 4.27 | 0.0 | 67.0 |
| Cr33.33(CoCuFeNi)66.7 | FCC | 6.38 | BCC | 0.17 | Uncertain | 1.9 | 3.03 | 33.2 | 33.6 |
| CrTiV | BCC | 5.00 | BCC | 1.67 | Uncertain | -5.5 | 2.39 | 0.0 | 66.7 |
| AlCoCrCuFeNi | BCC | 7.83 | Uncertain | 1.31 | Uncertain | -34.2 | 10.02 | 50.0 | 16.7 |
| AlCoCuNiTiZn | BCC | 8.17 | FCC | 1.36 | Uncertain | -75.9 | 11.59 | 50.0 | 0.0 |
| AlCrCuFeTiZn | BCC | 7.33 | Uncertain | 1.22 | Uncertain | -52.0 | 12.37 | 33.3 | 16.7 |
| MoNbTaTiVZr | BCC | 4.83 | BCC | 0.81 | Uncertain | -4.1 | 6.71 | 0.0 | 66.7 |
| Al2CoCrFeNi | BCC | 6.50 | BCC | 1.08 | Uncertain | -48.7 | 12.96 | 50.0 | 16.7 |
| AlCoCrFeNiV | BCC | 6.83 | BCC | 1.14 | Uncertain | -68.3 | 11.20 | 33.3 | 33.3 |
| AlMo0.5NbTa0.5TiZr0.5 | BCC | 4.33 | BCC | 0.96 | Uncertain | -63.7 | 18.38 | 22.2 | 44.4 |
| CoCrNi | FCC | 8.33 | FCC | 2.78 | Uncertain | -10.8 | 4.76 | 33.3 | 33.3 |
| CoCuNi | FCC | 10.00 | FCC | 3.33 | Uncertain | 6.6 | 1.93 | 66.7 | 0.0 |
| AlCoCuFeNiV | FCC | 7.67 | Uncertain | 1.28 | Uncertain | -49.0 | 10.71 | 50.0 | 16.7 |
| Al0.4CoCu0.6Ni | FCC | 8.93 | FCC | 2.98 | Uncertain | -22.0 | 7.52 | 66.7 | 0.0 |
| Al0.5CoCrCu0.5FeNi2 | FCC | 8.33 | FCC | 1.39 | Uncertain | -30.1 | 7.77 | 50.0 | 16.7 |
| Al0.5CoCrCuFeNiV0.4 | FCC | 8.05 | FCC | 1.37 | Uncertain | -37.2 | 8.54 | 42.4 | 23.7 |
| Al0.8824CoCrCuFeNi | FCC | 7.93 | Uncertain | 1.35 | Uncertain | -33.1 | 9.67 | 49.0 | 17.0 |
| Al0.4CoCu0.6NiSi0.05 | FCC | 8.85 | FCC | 2.90 | Uncertain | -24.7 | 8.06 | 65.6 | 0.0 |
| Al2.3B0.6CoCrCu0.7FeNiSi0.1 | BCC | 6.47 | BCC | 0.84 | Uncertain | -48.7 | 16.59 | 51.9 | 13.0 |
| AlCoCrCu0.5FeNi | BCC | 7.55 | Uncertain | 1.37 | Uncertain | -40.4 | 10.43 | 45.5 | 18.2 |
| CoCrCu1.5FeNi | FCC | 9.00 | FCC | 1.64 | FCC | 2.8 | 4.76 | 45.5 | 18.2 |
| Al0.5CoCrCuFeNi | FCC | 8.27 | FCC | 1.50 | Uncertain | -26.3 | 8.19 | 45.5 | 18.2 |
| Al0.4945CoCrCuFeNi | FCC | 8.28 | FCC | 1.51 | Uncertain | -26.2 | 8.16 | 45.4 | 18.2 |
| AlCoCrCuFeNiWZr | BCC | 7.13 | Uncertain | 0.89 | Uncertain | -79.9 | 16.22 | 37.5 | 25.0 |
| AlNbTa0.5TiZr0.5 | BCC | 4.13 | BCC | 1.03 | Uncertain | -60.4 | 19.45 | 25.0 | 37.5 |
| CoCrFe0.2Ni | FCC | 8.31 | FCC | 2.60 | Uncertain | -12.2 | 4.61 | 31.3 | 31.3 |
| CrCu2Fe2MnNi2 | FCC | 8.88 | FCC | 1.11 | Uncertain | -2.8 | 4.67 | 50.0 | 12.5 |
| CoCrFeMnNi3V | FCC | 8.13 | FCC | 1.02 | Uncertain | -28.8 | 5.10 | 37.5 | 25.0 |
| Mo1.3NbTiVZr | BCC | 4.87 | BCC | 0.92 | Uncertain | -4.9 | 6.77 | 0.0 | 62.3 |
| AlCoCrCu0.25FeNi | BCC | 7.38 | Uncertain | 1.41 | Uncertain | -45.0 | 10.66 | 42.9 | 19.0 |
| CoCrCuFeIn0.2466Ni | FCC | 8.53 | FCC | 1.63 | FCC | 2.5 | 4.57 | 38.1 | 19.1 |
| Co19Cr19.2Cu23.5Fe19.2Ni19.1 | FCC | 8.89 | FCC | 0.09 | Uncertain | 1.7 | 4.72 | 42.6 | 19.2 |
| Nb4(CoCrCuFeNi)96 | FCC | 7.11 | Uncertain | 0.79 | Uncertain | -2.2 | 13.10 | 38.4 | 23.2 |
| AlCoCrFeMo0.1Ni | BCC | 7.18 | Uncertain | 1.41 | Uncertain | -52.8 | 11.42 | 39.2 | 21.6 |
| AlCoCrFeNb0.1Ni | BCC | 7.16 | Uncertain | 1.40 | Uncertain | -58.9 | 11.68 | 39.2 | 21.6 |
| Sc0.03(Al2CoCrFeNi)0.97 | BCC | 6.48 | BCC | 1.08 | Uncertain | -51.4 | 13.06 | 39.4 | 19.7 |
| AlCoCrCuFe | BCC | 7.40 | Uncertain | 1.48 | Uncertain | -25.9 | 10.99 | 40.0 | 20.0 |
| AlCoCrFeNi | BCC | 7.20 | Uncertain | 1.44 | Uncertain | -51.2 | 10.90 | 40.0 | 20.0 |
| AlCoCrNiSi | BCC | 6.40 | BCC | 1.28 | Uncertain | -48.6 | 15.15 | 40.0 | 20.0 |
| AlCrFeTiZn | BCC | 6.60 | BCC | 1.32 | Uncertain | -61.9 | 13.33 | 20.0 | 20.0 |
| AlCuFeNiTi | BCC | 7.20 | Uncertain | 1.44 | Uncertain | -66.7 | 12.59 | 60.0 | 0.0 |
| MoTaTiVZr | BCC | 4.80 | BCC | 0.96 | Uncertain | -4.2 | 7.13 | 0.0 | 60.0 |
| NbTaTiVZr | BCC | 4.60 | BCC | 0.92 | Uncertain | 3.1 | 7.35 | 0.0 | 60.0 |
| Al18Co20Cr21Fe20Ni21 | BCC | 7.30 | Uncertain | 0.07 | Uncertain | -50.7 | 10.51 | 39.0 | 21.0 |
| Al0.5CrNbTi2V0.5 | BCC | 4.60 | BCC | 0.92 | Uncertain | -44.1 | 12.20 | 10.0 | 50.0 |
| AlNb1.5Ta0.5Ti1.5Zr0.5 | BCC | 4.20 | BCC | 0.84 | Uncertain | -57.4 | 17.61 | 20.0 | 40.0 |
| AlMo0.5NbTa0.5TiZr | BCC | 4.30 | BCC | 0.86 | Uncertain | -65.6 | 17.88 | 20.0 | 40.0 |
| Al2CoCrCuFeMnNiTiV | BCC | 6.60 | BCC | 0.66 | Uncertain | -88.3 | 12.54 | 40.0 | 20.0 |
| CoCrCuFeNi | FCC | 8.80 | FCC | 1.76 | FCC | 0.5 | 4.67 | 40.0 | 20.0 |
| CoCuFeNiV | FCC | 8.60 | FCC | 1.72 | FCC | -13.9 | 5.79 | 40.0 | 20.0 |
| CuFeMnNiPt | FCC | 9.20 | FCC | 1.84 | Uncertain | -27.6 | 7.08 | 60.0 | 0.0 |
| Ni40(CoCrFe)60 | FCC | 9.84 | FCC | 0.23 | Uncertain | -14.5 | 1.85 | 40.0 | 20.0 |
| Al0.3CoCrFeNi1.7 | FCC | 8.18 | FCC | 1.64 | FCC | -34.3 | 7.02 | 40.0 | 20.0 |
| Al13Co20Cr23.5Fe20Ni23.5 | FCC | 7.55 | Uncertain | 0.08 | Uncertain | -46.9 | 9.38 | 36.5 | 23.5 |
| Co4(AlCoCrFeNi)96 | FCC | 7.20 | Uncertain | 1.44 | Uncertain | -51.1 | 10.90 | 40.0 | 20.0 |
| Co15Cu25Fe15Mn10Ni35 | FCC | 9.50 | FCC | 0.10 | Uncertain | 0.0 | 3.27 | 60.0 | 0.0 |
| Al0.5CoCrCu0.5FeNi | FCC | 8.00 | FCC | 1.60 | Uncertain | -33.3 | 8.47 | 40.0 | 20.0 |
| Al0.5CoCrFeMo0.5Ni | FCC | 7.50 | Uncertain | 1.50 | Uncertain | -47.5 | 11.01 | 30.0 | 30.0 |
| Al0.9CoCrFeNi | BCC | 7.29 | Uncertain | 1.49 | Uncertain | -50.8 | 10.57 | 38.8 | 20.4 |
| CoCrFe0.4Ni | FCC | 8.29 | FCC | 2.44 | Uncertain | -13.1 | 4.47 | 29.4 | 29.4 |
| Al0.85CoCrFeNi | BCC | 7.33 | Uncertain | 1.51 | Uncertain | -50.5 | 10.39 | 38.1 | 20.6 |
| Mo1.5NbTiV0.3Zr | BCC | 4.90 | BCC | 1.02 | Uncertain | -5.0 | 5.96 | 0.0 | 58.3 |
| Al1.5CoCrFeNiTi0.5 | BCC | 6.58 | BCC | 1.10 | Uncertain | -80.0 | 12.78 | 41.7 | 16.7 |
| Al0.3CoCrCu0.5FeNi | FCC | 8.21 | FCC | 1.71 | FCC | -26.0 | 7.29 | 37.5 | 20.8 |
| Al0.6CoCrCu0.4FeNiSi0.2 | BCC | 7.69 | Uncertain | 1.48 | Uncertain | -45.6 | 10.29 | 38.5 | 19.2 |
| Al0.8CoCrCu0.2FeNiSi0.2 | BCC | 7.39 | Uncertain | 1.42 | Uncertain | -52.2 | 11.10 | 38.5 | 19.2 |
| Al0.9CoCrCu0.1FeNiSi0.2 | BCC | 7.23 | Uncertain | 1.39 | Uncertain | -55.1 | 11.48 | 38.5 | 19.2 |
| Al0.2CoCrCu0.8FeNiSi0.2 | FCC | 8.31 | FCC | 1.60 | Uncertain | -26.1 | 8.33 | 38.5 | 19.2 |
| Al0.4CoCrCu0.6FeNiSi0.2 | FCC | 8.00 | Uncertain | 1.54 | Uncertain | -37.2 | 9.37 | 38.5 | 19.2 |
| Ni42.9(CoCrFeMn)57.1 | FCC | 9.79 | FCC | 0.21 | Uncertain | -18.0 | 2.09 | 42.9 | 14.3 |
| CrCuFeMn2Ni2 | FCC | 8.43 | FCC | 1.20 | Uncertain | -8.6 | 4.65 | 42.9 | 14.3 |
| CoCrFeMnNi2V | FCC | 7.86 | Uncertain | 1.12 | Uncertain | -29.0 | 5.05 | 28.6 | 28.6 |
| Al0.3B0.15CoCrFeNiCu0.7Si0.1 | FCC | 8.09 | FCC | 1.54 | Uncertain | -34.3 | 10.80 | 38.1 | 19.0 |
| Al0.65CoCrFeNi | FCC | 7.52 | Uncertain | 1.62 | FCC | -48.0 | 9.56 | 35.5 | 21.5 |
| Al0.5CoCrFeMo0.1Ni | FCC | 7.63 | Uncertain | 1.66 | FCC | -46.1 | 9.39 | 32.6 | 23.9 |
| MoNbTiV0.5Zr | BCC | 4.78 | BCC | 1.06 | Uncertain | -3.8 | 6.54 | 0.0 | 55.6 |
| Al0.5Mo0.5NbTa0.5TiZr | BCC | 4.44 | BCC | 0.99 | Uncertain | -56.4 | 14.07 | 11.1 | 44.4 |
| CoCrFe0.6Ni | FCC | 8.28 | FCC | 2.30 | Uncertain | -13.9 | 4.34 | 27.8 | 27.8 |
| CoFeNi2W0.5 | FCC | 8.89 | FCC | 1.98 | Uncertain | -8.5 | 8.53 | 44.4 | 11.1 |
| Al0.5CoCrFeNi | FCC | 7.67 | Uncertain | 1.70 | FCC | -44.6 | 8.79 | 33.3 | 22.2 |
| CoCrCu0.5FeNi | FCC | 8.56 | FCC | 1.90 | Uncertain | -4.4 | 4.48 | 33.3 | 22.2 |
| Cr2CuFe2Mn2Ni2 | FCC | 8.11 | FCC | 0.90 | Uncertain | -7.9 | 4.60 | 33.3 | 22.2 |
| CoCrFeMo0.5Ni | FCC | 8.00 | FCC | 1.78 | FCC | -16.1 | 7.74 | 22.2 | 33.3 |
| Al0.3B0.3CoCrFeNiCu0.7Si0.1 | FCC | 7.94 | Uncertain | 1.47 | Uncertain | -37.4 | 12.82 | 37.0 | 18.5 |
| Al0.45CoCrFeNi | FCC | 7.72 | Uncertain | 1.74 | FCC | -43.1 | 8.49 | 32.6 | 22.5 |
| Co20Cr20Fe20Mn5Ni20Zn15 | FCC | 8.75 | FCC | 0.09 | Uncertain | -28.3 | 5.01 | 20.0 | 20.0 |
| Al0.75CoCrCu0.25FeNiTi0.5 | BCC | 7.27 | Uncertain | 1.32 | Uncertain | -72.7 | 10.46 | 36.4 | 18.2 |
| Al0.4CoCrFeNi | FCC | 7.77 | Uncertain | 1.77 | FCC | -41.5 | 8.18 | 31.8 | 22.7 |
| Al0.3CoCrFeMo0.1Ni | FCC | 7.84 | Uncertain | 1.78 | FCC | -39.1 | 8.17 | 29.5 | 25.0 |
| CoCrCuFeNiTi0.5 | FCC | 8.36 | FCC | 1.52 | Uncertain | -24.4 | 6.04 | 36.4 | 18.2 |
| Al0.25CoCrCu0.75FeNiTi0.5 | FCC | 8.00 | FCC | 1.46 | Uncertain | -48.2 | 7.83 | 36.4 | 18.2 |
| Al0.375CoCrFeNi | FCC | 7.80 | Uncertain | 1.78 | FCC | -40.6 | 8.01 | 31.4 | 22.9 |
| AlCrFeMo0.5NiSiTi | BCC | 5.85 | BCC | 0.90 | Uncertain | -91.5 | 17.24 | 30.8 | 23.1 |
| Mo0.3NbTiVZr | BCC | 4.61 | BCC | 1.07 | Uncertain | -0.7 | 7.52 | 0.0 | 53.5 |
| Al0.3CoCrFeNi | FCC | 7.88 | Uncertain | 1.83 | Uncertain | -37.5 | 7.47 | 30.2 | 23.3 |
| CoCrFeMo0.3Ni | FCC | 8.09 | FCC | 1.88 | Uncertain | -16.2 | 6.77 | 23.3 | 30.2 |
| Al6.64Co23.82Cr23.66Fe23.01Ni22.87 | FCC | 7.89 | Uncertain | 0.08 | Uncertain | -36.8 | 7.36 | 29.5 | 23.7 |
| Al0.25NbTaTiZr | BCC | 4.41 | BCC | 1.04 | Uncertain | -27.0 | 11.13 | 5.9 | 47.1 |
| Al0.25CoCrFeNi | FCC | 7.94 | Uncertain | 1.87 | Uncertain | -35.0 | 7.07 | 29.4 | 23.5 |
| Al0.3B0.6CoCrFeNiCu0.7Si0.1 | FCC | 7.68 | Uncertain | 1.35 | Uncertain | -38.1 | 15.83 | 35.1 | 17.5 |
| Mo5(NbTaTiZr)95 | BCC | 5.33 | BCC | 0.59 | Uncertain | 5.3 | 4.15 | 0.0 | 52.5 |
| Al0.2CoCrFeNi | FCC | 8.00 | FCC | 1.91 | Uncertain | -32.1 | 6.62 | 28.6 | 23.8 |
| Al0.3CoCrFeMn0.1Ni | FCC | 7.86 | Uncertain | 1.79 | FCC | -39.9 | 7.42 | 29.5 | 22.7 |
| Al0.3CoCrFeNiTi0.1 | FCC | 7.80 | Uncertain | 1.77 | FCC | -47.5 | 7.69 | 29.5 | 22.7 |
| Al1.2CrFe1.5MnNi0.5 | BCC | 6.46 | BCC | 1.24 | Uncertain | -54.0 | 11.95 | 32.7 | 19.2 |
| Co24.1Cr24.1Fe24.1Mo3.6Ni24.1 | FCC | 8.17 | FCC | 0.08 | Uncertain | -15.9 | 5.71 | 24.1 | 27.7 |
| AlCoCrFeNiSi0.8 | BCC | 6.76 | BCC | 1.17 | Uncertain | -56.9 | 13.80 | 34.5 | 17.2 |
| Al0.1CoCrFeNi | FCC | 8.12 | FCC | 1.98 | Uncertain | -24.7 | 5.56 | 26.8 | 24.4 |
| CoCrFeNiTa0.1 | FCC | 8.17 | FCC | 1.99 | Uncertain | -21.4 | 5.94 | 24.4 | 26.8 |
| Al4.88Co29.53Cr18.58Fe19.62Ni27.39 | FCC | 8.23 | FCC | 0.08 | Uncertain | -30.9 | 6.46 | 32.3 | 18.6 |
| CoCrCuFeGe0.9666Ni | FCC | 8.02 | FCC | 1.35 | Uncertain | -2.3 | 8.34 | 33.5 | 16.8 |
| Co25.33Cr25.77Fe24.53Ni24.37 | FCC | 8.23 | FCC | 0.08 | Uncertain | -14.9 | 4.15 | 24.4 | 25.8 |
| AlCoFeNi | BCC | 7.50 | Uncertain | 1.88 | Uncertain | -42.8 | 10.49 | 50.0 | 0.0 |
| AlCrFeTi | BCC | 5.25 | BCC | 1.31 | Uncertain | -59.1 | 14.26 | 25.0 | 25.0 |
| MoTiVZr | BCC | 4.75 | BCC | 1.19 | Uncertain | -3.9 | 7.33 | 0.0 | 50.0 |
| NbTaTiZr | BCC | 4.50 | BCC | 1.13 | Uncertain | 6.3 | 5.83 | 0.0 | 50.0 |
| NbTiVZr | BCC | 4.50 | BCC | 1.13 | Uncertain | 2.8 | 7.80 | 0.0 | 50.0 |
| AlCoCrFeNiTi | BCC | 6.67 | BCC | 1.11 | Uncertain | -88.2 | 11.79 | 33.3 | 16.7 |
| CoCrFeMnNiW | BCC | 7.67 | Uncertain | 1.28 | Uncertain | -11.3 | 8.97 | 16.7 | 33.3 |
| HfMoNbTaTiZr | BCC | 4.67 | BCC | 0.78 | Uncertain | -0.8 | 5.50 | 0.0 | 50.0 |
| HfNbTaTiVZr | BCC | 4.50 | BCC | 0.75 | Uncertain | 4.4 | 7.45 | 0.0 | 50.0 |
| Ag2Cu2DyGdTbY | BCC | 10.00 | FCC | 1.25 | Uncertain | -67.4 | 18.91 | 50.0 | 0.0 |
| HfMo1NbTaTiZr | BCC | 4.67 | BCC | 0.78 | Uncertain | -0.8 | 5.50 | 0.0 | 50.0 |
| CoCr5Fe5MoNbSiTiW | BCC | 6.50 | BCC | 0.41 | Uncertain | -45.9 | 11.89 | 0.0 | 50.0 |
| CoCrFeNi | FCC | 8.25 | FCC | 2.06 | Uncertain | -15.0 | 4.12 | 25.0 | 25.0 |
| CoCrMnNi | FCC | 8.00 | FCC | 2.00 | Uncertain | -17.0 | 4.34 | 25.0 | 25.0 |
| CoCuFeNi | FCC | 9.50 | FCC | 2.38 | Uncertain | 4.4 | 2.68 | 50.0 | 0.0 |
| CoFeNiV | FCC | 8.00 | FCC | 2.00 | Uncertain | -27.8 | 5.39 | 25.0 | 25.0 |
| CoCrFeMnNiCu | FCC | 8.50 | FCC | 1.42 | Uncertain | -5.5 | 4.59 | 33.3 | 16.7 |
| CoCrFeMnNiNb | FCC | 7.50 | Uncertain | 1.25 | Uncertain | -38.1 | 9.98 | 16.7 | 33.3 |
| CoCrFeMnNiV | FCC | 7.50 | Uncertain | 1.25 | Uncertain | -26.8 | 4.83 | 16.7 | 33.3 |
| Al7.5Co25 Cu17.5Fe25 Ni25 | FCC | 8.90 | FCC | 0.09 | Uncertain | -18.4 | 6.36 | 50.0 | 0.0 |
| CrFeMnNi | FCC | 7.75 | Uncertain | 1.94 | Uncertain | -16.6 | 3.98 | 25.0 | 25.0 |
| Al0.3CoCrFeMn0.3Ni | FCC | 7.83 | Uncertain | 1.70 | FCC | -42.4 | 7.31 | 28.3 | 21.7 |
| Co10Cr15Fe35Mn5Ni25V10 | FCC | 7.95 | Uncertain | 0.08 | Uncertain | -27.3 | 4.56 | 25.0 | 25.0 |
| Co33.33(CrCuFeNi)66.7 | FCC | 8.97 | FCC | 0.24 | Uncertain | 0.5 | 1.76 | 33.2 | 16.6 |
| C0.05CoCrFeNi | FCC | 8.20 | FCC | 2.02 | Uncertain | -14.9 | 7.56 | 24.7 | 24.7 |
| CoCuFeNiSn0.05 | FCC | 9.43 | FCC | 2.33 | Uncertain | 3.3 | 2.69 | 49.4 | 0.0 |
| Al10Co17Fe34Mo5Ni34 | FCC | 8.25 | FCC | 0.08 | Uncertain | -37.8 | 9.26 | 44.0 | 5.0 |
| Al0.4Hf0.6NbTaTiZr | BCC | 4.32 | BCC | 0.86 | Uncertain | -43.4 | 12.61 | 8.0 | 40.0 |
| Al0.8CrFe1.5MnNi0.5 | BCC | 6.75 | BCC | 1.41 | Uncertain | -51.8 | 10.53 | 27.1 | 20.8 |
| Al0.3CrFe1.5MnNi | BCC | 7.48 | Uncertain | 1.56 | Uncertain | -39.1 | 7.23 | 27.1 | 20.8 |
| Co5(CrFeMnNi)95 | FCC | 8.44 | FCC | 0.94 | Uncertain | -17.0 | 3.33 | 23.8 | 23.8 |
| Al0.2CoCrFeNiTi0.5 | FCC | 7.57 | Uncertain | 1.61 | FCC | -58.5 | 7.54 | 25.5 | 21.3 |
| AlCoCrFeMo0.5NiSiTi | BCC | 6.27 | BCC | 0.84 | Uncertain | -101.0 | 16.02 | 26.7 | 20.0 |
| Ni20(CoCrFe)80 | FCC | 9.70 | FCC | 0.42 | Uncertain | -12.3 | 2.47 | 20.0 | 26.7 |
| CoCrFeNiTi0.3 | FCC | 7.95 | Uncertain | 1.85 | Uncertain | -33.0 | 5.13 | 23.3 | 23.3 |
| Co1.5CrFeMo0.1Ni1.5Ti0.5 | FCC | 8.05 | FCC | 1.44 | Uncertain | -37.0 | 6.04 | 26.8 | 19.6 |
| Al0.3NbTa0.8Ti1.4V0.2Zr1.3 | BCC | 4.34 | BCC | 0.87 | Uncertain | -30.6 | 11.55 | 6.0 | 40.0 |
| HfMoNb1.5TiZr | BCC | 4.64 | BCC | 0.84 | Uncertain | -1.2 | 5.70 | 0.0 | 45.5 |
| HfMo1.5NbTiZr | BCC | 4.73 | BCC | 0.86 | Uncertain | -2.4 | 5.77 | 0.0 | 45.5 |
| Co1.5CrFeNi1.5Ti0.5 | FCC | 8.09 | FCC | 1.47 | Uncertain | -36.6 | 5.41 | 27.3 | 18.2 |
| Al0.5CoCrFeMnNi | FCC | 7.55 | Uncertain | 1.37 | Uncertain | -51.4 | 8.21 | 27.3 | 18.2 |
| C9.302(CoCrFeNi)90.698 | FCC | 5.28 | BCC | 0.40 | Uncertain | -14.0 | 43.60 | 22.7 | 22.7 |
| Co15Cr20Fe20Mn20Ni25 | FCC | 8.05 | FCC | 0.08 | Uncertain | -19.5 | 4.00 | 25.0 | 20.0 |
| Co10(CrFeMnNi)90 | FCC | 8.64 | FCC | 0.62 | Uncertain | -17.4 | 2.81 | 22.5 | 22.5 |
| Co35Cr15Fe20 Mo10Ni20 | FCC | 8.25 | FCC | 0.08 | Uncertain | -15.1 | 7.53 | 20.0 | 25.0 |
| Al8(CoCrFeMnNi)92 | FCC | 4.92 | BCC | 0.38 | Uncertain | -27.8 | 14.48 | 26.4 | 18.4 |
| Al0.5CrFe1.5MnNi0.5 | BCC | 7.00 | Uncertain | 1.56 | Uncertain | -45.6 | 8.95 | 22.2 | 22.2 |
| HfMoNbTi0.5Zr | BCC | 4.67 | BCC | 1.04 | Uncertain | -1.4 | 5.17 | 0.0 | 44.4 |
| HfMoNbTiZr0.5 | BCC | 4.67 | BCC | 1.04 | Uncertain | -1.7 | 6.05 | 0.0 | 44.4 |
| Hf0.5MoNbTiZr | BCC | 4.67 | BCC | 1.04 | Uncertain | -2.1 | 5.93 | 0.0 | 44.4 |
| C11.01(CoCrFeNi)88.99 | FCC | 5.13 | BCC | 0.34 | Uncertain | -13.5 | 43.44 | 22.2 | 22.2 |
| CoCrFeMn0.5Ni | FCC | 8.11 | FCC | 1.80 | Uncertain | -18.0 | 4.01 | 22.2 | 22.2 |
| CoCrFeNiTi0.5 | FCC | 7.78 | Uncertain | 1.73 | FCC | -38.2 | 5.56 | 22.2 | 22.2 |
| Mn14(CoCrFeNi)86 | FCC | 7.28 | Uncertain | 0.40 | Uncertain | -16.4 | 2.33 | 21.5 | 21.5 |
| HfNb2.0TiVZr2.0 | BCC | 4.43 | BCC | 0.63 | Uncertain | 4.1 | 7.17 | 0.0 | 42.9 |
| CoCrCu0.25FeMnNi | FCC | 8.14 | FCC | 1.55 | Uncertain | -13.0 | 4.14 | 23.8 | 19.0 |
| CoCrFeMnNiV0.25 | FCC | 7.86 | Uncertain | 1.50 | Uncertain | -23.7 | 4.25 | 19.0 | 23.8 |
| AlCoFeNiTi | BCC | 6.80 | BCC | 1.36 | Uncertain | -88.7 | 12.42 | 40.0 | 0.0 |
| CuNiSiTiZr | BCC | 6.60 | BCC | 1.32 | Uncertain | -95.9 | 20.26 | 40.0 | 0.0 |
| HfMoNbTiZr | BCC | 4.60 | BCC | 0.92 | Uncertain | -1.5 | 5.98 | 0.0 | 40.0 |
| HfMoTaTiZr | BCC | 4.60 | BCC | 0.92 | Uncertain | -2.3 | 6.02 | 0.0 | 40.0 |
| HfNbTaTiZr | BCC | 4.40 | BCC | 0.88 | Uncertain | 8.0 | 5.78 | 0.0 | 40.0 |
| HfNbTiVZr | BCC | 4.40 | BCC | 0.88 | Uncertain | 3.3 | 8.03 | 0.0 | 40.0 |
| Al20Co20Cr20(FeMn)40 | BCC | 6.05 | BCC | 0.10 | Uncertain | -48.1 | 13.70 | 20.0 | 20.0 |
| Al0.3CrFe1.5MnNi0.5Ti0.2 | BCC | 7.04 | Uncertain | 1.57 | Uncertain | -49.3 | 7.71 | 17.8 | 22.2 |
| CoCrFeMn0.5NiTi0.5 | BCC | 7.70 | Uncertain | 1.54 | Uncertain | -39.9 | 5.29 | 20.0 | 20.0 |
| CoCrFeMnNi | FCC | 8.00 | FCC | 1.60 | Uncertain | -19.0 | 3.90 | 20.0 | 20.0 |
| CoCrFeNiTi | FCC | 7.40 | Uncertain | 1.48 | Uncertain | -42.6 | 6.21 | 20.0 | 20.0 |
| CoCuFeMnNi | FCC | 9.00 | FCC | 1.80 | Uncertain | -5.5 | 3.63 | 40.0 | 0.0 |
| CoCuFeNiTi | FCC | 8.40 | FCC | 1.68 | FCC | -35.6 | 6.95 | 40.0 | 0.0 |
| CrTiVYZr | FCC | 4.40 | BCC | 0.88 | Uncertain | 18.1 | 10.19 | 0.0 | 40.0 |
| Ni40(CoFeMn)60 | FCC | 9.86 | FCC | 0.23 | Uncertain | -19.2 | 1.41 | 40.0 | 0.0 |
| Co20(CrFeMnNi)80 | FCC | 8.79 | FCC | 0.37 | Uncertain | -18.0 | 2.22 | 20.0 | 20.0 |
| Co5Cu15Fe30Mn25Ni25 | FCC | 8.75 | FCC | 0.09 | Uncertain | -10.1 | 3.68 | 40.0 | 0.0 |
| CoCuFe0.25Mn1.75Ni | FCC | 8.85 | FCC | 1.77 | FCC | -8.8 | 4.07 | 40.0 | 0.0 |
| C0.01CoCrFeMnNi | FCC | 7.99 | Uncertain | 1.60 | Uncertain | -19.2 | 4.66 | 20.0 | 20.0 |
| NbTiV0.3Zr | BCC | 4.39 | BCC | 1.33 | Uncertain | 4.1 | 7.16 | 0.0 | 39.4 |
| CoCrFeMnNiTi0.1 | FCC | 7.92 | Uncertain | 1.55 | Uncertain | -25.6 | 4.21 | 19.6 | 19.6 |
| CoCu0.9 Fe1.05Mn1.05Ni | FCC | 8.93 | FCC | 1.79 | FCC | -6.5 | 3.60 | 38.0 | 0.0 |
| Al0.5CoFeNiSi0.5 | BCC | 7.63 | Uncertain | 1.91 | Uncertain | -46.0 | 11.66 | 37.5 | 0.0 |
| Hf0.5Mo0.5NbTiZr | BCC | 4.50 | BCC | 1.13 | Uncertain | 0.1 | 6.22 | 0.0 | 37.5 |
| Co42.5Cr12.5Fe20Mo5Ni20 | FCC | 8.48 | FCC | 0.09 | Uncertain | -14.1 | 5.98 | 20.0 | 17.5 |
| HfMoNbTi1.5Zr | BCC | 4.55 | BCC | 0.83 | Uncertain | -1.5 | 6.44 | 0.0 | 36.4 |
| HfMoNbTiZr1.5 | BCC | 4.55 | BCC | 0.83 | Uncertain | -1.3 | 5.87 | 0.0 | 36.4 |
| Hf1.5MoNbTiZr | BCC | 4.55 | BCC | 0.83 | Uncertain | -1.1 | 5.95 | 0.0 | 36.4 |
| Ni15(CoCrFeMn)85 | FCC | 9.47 | FCC | 0.50 | Uncertain | -13.4 | 3.08 | 15.0 | 21.3 |
| Al0.3CoFeNiSi0.3 | FCC | 8.08 | FCC | 2.25 | Uncertain | -40.8 | 9.87 | 36.1 | 0.0 |
| Al0.2CoFeNiSi0.2 | FCC | 8.35 | FCC | 2.46 | Uncertain | -34.9 | 8.48 | 35.3 | 0.0 |
| AlFeTi | BCC | 5.00 | BCC | 1.67 | Uncertain | -62.4 | 16.04 | 33.3 | 0.0 |
| HfNbZr | BCC | 4.33 | BCC | 1.44 | Uncertain | 6.9 | 2.12 | 0.0 | 33.3 |
| CoCuHfPdTiZr | BCC | 7.00 | Uncertain | 1.17 | Uncertain | -128.6 | 13.74 | 33.3 | 0.0 |
| HfMoNb0.5TiZr | BCC | 4.56 | BCC | 1.01 | Uncertain | -2.2 | 6.29 | 0.0 | 33.3 |
| Co0.5Fe0.5MgNi0.5TiZr | BCC | 5.22 | BCC | 1.16 | Uncertain | -50.1 | 13.81 | 11.1 | 0.0 |
| CoFeNi | FCC | 9.00 | FCC | 3.00 | Uncertain | -10.4 | 1.88 | 33.3 | 0.0 |
| CoMnNi | FCC | 8.67 | FCC | 2.89 | Uncertain | -17.0 | 3.31 | 33.3 | 0.0 |
| FeMnNi | FCC | 8.33 | FCC | 2.78 | Uncertain | -20.8 | 3.17 | 33.3 | 0.0 |
| CoFeNi(AlCu)0.2 | FCC | 8.20 | FCC | 1.64 | FCC | -21.6 | 9.37 | 33.3 | 0.0 |
| CoFeNi(AlCu)0.4 | FCC | 8.20 | FCC | 1.64 | FCC | -26.6 | 9.37 | 33.3 | 0.0 |
| CoFeNi(AlCu)0.6 | FCC | 8.20 | FCC | 1.64 | FCC | -28.7 | 9.37 | 33.3 | 0.0 |
| CoFeNi(AlCu)0.7 | FCC | 8.20 | FCC | 1.64 | FCC | -29.0 | 9.37 | 33.3 | 0.0 |
| CoFeNi(AlCu)0.8 | FCC | 8.20 | FCC | 1.64 | FCC | -29.3 | 9.37 | 33.3 | 0.0 |
| Co33.33(CrFeMnNi)66.7 | FCC | 8.87 | FCC | 0.24 | Uncertain | -18.7 | 1.82 | 16.6 | 16.6 |
| Al0.3CoFeNiSi | BCC | 7.42 | Uncertain | 1.73 | Uncertain | -32.1 | 13.16 | 30.2 | 0.0 |
| Hf15Nb20Ta10Ti30Zr25 | BCC | 4.30 | BCC | 0.04 | Uncertain | 7.3 | 6.65 | 0.0 | 30.0 |
| Co30Fe30Mn10Ni30 | FCC | 8.80 | FCC | 0.09 | Uncertain | -15.3 | 2.46 | 30.0 | 0.0 |
| Co30Fe30Ni30Ti10 | FCC | 8.50 | FCC | 0.09 | Uncertain | -35.7 | 4.92 | 30.0 | 0.0 |
| Co26Fe27Mn10Ni27Ti10 | FCC | 8.30 | FCC | 0.08 | Uncertain | -40.5 | 4.97 | 27.0 | 0.0 |
| HfNbTiZr | BCC | 4.25 | BCC | 1.06 | Uncertain | 6.5 | 6.44 | 0.0 | 25.0 |
| HfMo0.5Nb0.5TiZr | BCC | 4.38 | BCC | 1.09 | Uncertain | -0.5 | 6.57 | 0.0 | 25.0 |
| HfNb0.5Ta0.5TiZr | BCC | 4.25 | BCC | 1.06 | Uncertain | 6.9 | 6.45 | 0.0 | 25.0 |
| HfNb0.5TiV0.5Zr | BCC | 4.25 | BCC | 1.06 | Uncertain | 3.0 | 7.90 | 0.0 | 25.0 |
| AlCoCrFe6NiSiTi | BCC | 7.00 | Uncertain | 0.58 | Uncertain | -76.7 | 11.64 | 16.7 | 8.3 |
| CoFeMnNi | FCC | 8.50 | FCC | 2.13 | Uncertain | -19.5 | 2.91 | 25.0 | 0.0 |
| PbSnTeSe | FCC | 5.00 | BCC | 1.25 | Uncertain | 0.0 | 15.11 | 25.0 | 0.0 |
| Co25Cr25Fe25Mn25 | FCC | 7.50 | Uncertain | 0.08 | Uncertain | -10.7 | 3.32 | 0.0 | 25.0 |
| Al7.5Cr6Fe40.4Mn34.8Ni11.3 | FCC | 7.38 | Uncertain | 0.07 | Uncertain | -40.6 | 7.28 | 18.8 | 6.0 |
| Al7.4C1.1Cr5.55Fe39.93Mn35.67Ni10.35 | FCC | 7.33 | Uncertain | 0.07 | Uncertain | -39.5 | 9.39 | 17.8 | 5.6 |
| Pb0.9SnTeSeLa0.1 | FCC | 4.98 | BCC | 1.24 | Uncertain | 0.0 | 2.03 | 22.5 | 0.0 |
| CoCuFeTiZrHf | FCC | 6.67 | BCC | 1.11 | Uncertain | -59.3 | 6.06 | 16.7 | 0.0 |
| HfTa0.53TiZr | BCC | 4.15 | BCC | 1.18 | Uncertain | 4.4 | 5.42 | 0.0 | 15.0 |
| Hf27.5Nb5Ta5Ti35Zr27.5 | BCC | 4.10 | BCC | 0.04 | Uncertain | 4.6 | 6.51 | 0.0 | 10.0 |
| Co10Cr10Fe40Mn40 | FCC | 7.50 | Uncertain | 0.08 | Uncertain | -13.5 | 6.42 | 0.0 | 10.0 |
| Ni5(CoFeMn)95 | FCC | 9.25 | FCC | 1.16 | Uncertain | -16.4 | 7.03 | 5.0 | 0.0 |
| C69.23(Co10Cr10Fe40Mn40)30.77 | FCC | 6.07 | BCC | 0.04 | Uncertain | -14.6 | 2.69 | 0.0 | 3.2 |
| C77.34(Co10Cr10Fe40Mn40)22.66 | FCC | 5.97 | BCC | 0.03 | Uncertain | -14.2 | 7.82 | 0.0 | 2.9 |
| C82.15(Co10Cr10Fe40Mn40)17.85 | FCC | 5.92 | BCC | 0.03 | Uncertain | -13.1 | 8.91 | 0.0 | 2.7 |
| C87.60(Co10Cr10Fe40Mn40)12.4 | FCC | 5.87 | BCC | 0.03 | Uncertain | -9.6 | 7.58 | 0.0 | 2.6 |
| C90.71(Co10Cr10Fe40Mn40)9.29 | FCC | 5.84 | BCC | 0.03 | Uncertain | -3.8 | 6.53 | 0.0 | 2.5 |

**Supplementary Fig. 1.** **Thermodynamically predicted number of phases.** For each composition, the number of phases predicted by thermodynamics for a given temperature is recorded. Graphs A) and B) are for the non-allotrope compositions, C) and D) are for the Murty HEAs dataset, and E) and F) are for the Gorsse HEAs dataset. Panels A), C), and E) count the presence of the liquid phase (i.e., having only liquid counts as 1 phase and solid plus liquid is 2 phases) while panels B), D), and F) only count the solid phases present (i.e., having only liquid counts as 0 phases and solid plus liquid is 1 phase). The legend on the right corresponds with the number of phases.

**Supplementary Fig. 2.** **Phase evolution diagrams for fabricated materials.** Equilibrium phase diagrams from 2500°C to 500°C for the experimental samples A) Al_8.90_Cr_11.45_Nb_20.45_Ni_19.37_Ta_39.83_ B) Al_27.00_Cr_13.01_Mo_24.00_Nb_23.24_V_12.74_ C) Al_8.87_Nb_20.37_Ni_19.30_V_11.17_W_40.30_ D) Mo_14.64_Nb_14.17_Ni_35.81_Ta_27.60_V_7.77_ E) Cr_13.61_Cu_24.93_Mo_25.10_Ni_23.03_V_13.33_ F) Cu_19.11_Mo_14.42_Nb_13.97_Ni_17.65_Ta_27.20_V_7.66_ G) Cr_6.68_Cu_20.41_Mo_12.33_Nb_11.94_Ni_18.85_Ta_23.25_V_6.54_ H) Cr_5.08_Cu_18.65_Mo_9.37_Nb_9.07_Ni_17.21_Ta_17.68_V_4.98_W_17.96_ and I) Al_8.87_Cr_5.69_Mo_10.50_Nb_10.17_Ni_19.28_Ta_19.80_V_5.57_W_20.12_. All compositions are provided in weight percent.

**Supplementary Fig. 3.** **XRD patterns for fabricated materials.** XRD patterns for the experimental samples A) Al_8.90_Cr_11.45_Nb_20.45_Ni_19.37_Ta_39.83_ B) Al_27.00_Cr_13.01_Mo_24.00_Nb_23.24_V_12.74_ C) Al_8.87_Nb_20.37_Ni_19.30_V_11.17_W_40.30_ D) Mo_14.64_Nb_14.17_Ni_35.81_Ta_27.60_V_7.77_ E) Cr_13.61_Cu_24.93_Mo_25.10_Ni_23.03_V_13.33_ F) Cu_19.11_Mo_14.42_Nb_13.97_Ni_17.65_Ta_27.20_V_7.66_ G) Cr_6.68_Cu_20.41_Mo_12.33_Nb_11.94_Ni_18.85_Ta_23.25_V_6.54_ H) Cr_5.08_Cu_18.65_Mo_9.37_Nb_9.07_Ni_17.21_Ta_17.68_V_4.98_W_17.96_ and I) Al_8.87_Cr_5.69_Mo_10.50_Nb_10.17_Ni_19.28_Ta_19.80_V_5.57_W_20.12_. All compositions are provided in weight percent.

**
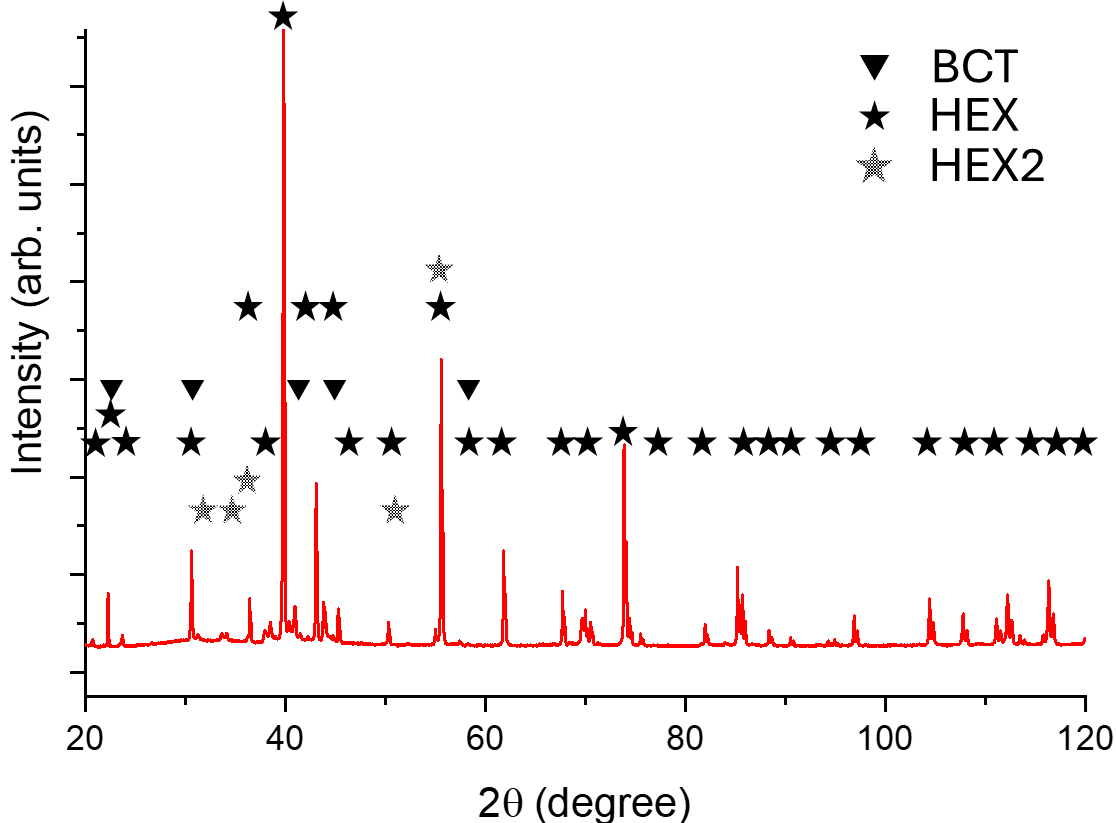
**

**Supplementary Fig. 4.** **XRD patterns for annealed Al_8.90_Cr_11.45_Nb_20.45_Ni_19.37_Ta_39.83_.** XRD pattern for the experimental sample Al_8.90_Cr_11.45_Nb_20.45_Ni_19.37_Ta_39.83_ post heat treatment at 1475°C for 20 hours.
